# Recombination-independent rapid convergent evolution of the gastric pathogen *Helicobacter pylori*

**DOI:** 10.1186/s12864-018-5231-7

**Published:** 2018-11-21

**Authors:** Sujay Chattopadhyay, Peter B. Chi, Vladimir N. Minin, Douglas E. Berg, Evgeni V. Sokurenko

**Affiliations:** 1 School of Biotechnology, Amrita Vishwa Vidyapeetham, Kollam, Kerala India; 2 Department of Mathematics and Statistics, Villanova University, Villanova, PA USA; 3 Department of Statistics, University of California, Irvine, California, USA; 4 Division of Infectious Diseases, Department of Medicine, University of California, San Diego, La Jolla, California, USA; 5 Department of Microbiology, University of Washington, Seattle, Washington, USA

**Keywords:** *Helicobacter pylori*, Gastric pathogen, Protein-coding genes, Convergent mutations, Adaptive evolution

## Abstract

**Background:**

*Helicobacter pylori* is a human stomach pathogen, naturally-competent for DNA uptake, and prone to homologous recombination. Extensive homoplasy (i.e., phylogenetically-unlinked identical variations) observed in *H. pylori* genes is considered a hallmark of such recombination. However, *H. pylori* also exhibits a high mutation rate. The relative adaptive role of homologous recombination and mutation in species diversity is a highly-debated issue in biology. Recombination results in homoplasy. While convergent mutation can also account for homoplasy, its contribution is thought to be minor. We demonstrate here that, contrary to dogma, convergent mutation is a key contributor to *Helicobacter pylori* homoplasy, potentially driven by adaptive evolution of proteins.

**Results:**

Our present genome-wide analysis shows that homoplastic nonsynonymous (amino acid replacement) changes are not typically accompanied by homoplastic synonymous (silent) variations. Moreover, the majority of the codon positions with homoplastic nonsynonymous changes also contain different (i.e. non-homoplastic) nonsynonymous changes arising from mutation only. This indicates that, to a considerable extent, nonsynonymous homoplasy is due to convergent mutations. High mutation rate or limited availability of evolvable sites cannot explain this excessive convergence, as suggested by our simulation studies. Rather, the genes with convergent mutations are overrepresented in distinct functional categories, suggesting possible selective responses to conditions such as distinct micro-niches in single hosts, and to differences in host genotype, physiology, habitat and diet.

**Conclusions:**

We propose that mutational convergence is a key player in *H. pylori*’s adaptation and extraordinary persistence in human hosts. High frequency of mutational convergence could be due to saturation of evolvable sites capable of responding to selection pressures, while the number of mutable residues is far from saturation. We anticipate a similar scenario of mutational vs. recombinational genome dynamics or plasticity for other naturally competent microbes where strong positive selection could favor frequent convergent mutations in adaptive protein evolution.

**Electronic supplementary material:**

The online version of this article (10.1186/s12864-018-5231-7) contains supplementary material, which is available to authorized users.

## Background

*Helicobacter pylori* is a human-adapted bacterial species that infects about half of the world’s population and is associated with chronic gastritis, stomach and duodenal ulcers, and gastric cancer [1]. It is particularly well adapted to the gastric mucosa, a highly variable environment that is hostile to virtually all other bacterial species, where it can persist for decades [2, 3]. *H. pylori*’s high adaptability to different gastric mucosal environments has been attributed to frequent homologous recombination between different strains, given that this species is highly competent for DNA uptake (transformation) [4–14]. Indeed, recombination allows for the fast emergence, spread and shuffling of different adaptive genetic changes within species. Recombination also leads to so-called genetic homoplasy – identical allelic variations in organisms with different overall ancestries. Homoplasy is extremely frequent across the genome of *H. pylori* (and other naturally competent bacteria) and is assumed to be both the result of and main evidence for rampant genetic recombination.

Besides homologous recombination, genetic homoplasy can also arise by convergent identical mutations occurring independently in different lineages [15–18]. We have shown previously that convergent mutations that change sequences and functions of encoded proteins contribute to adaptive evolution and often increased pathogenicity of *Escherichia coli* [19, 20], *Salmonella enterica* [20] and *Bartonella bacilliformis* [21]. However these species, unlike *H. pylori*, do not seem to be readily transformable. Untangling the contributions of recombination vs. convergent mutation in bacterial pathogens will contribute importantly to understanding the nature of homoplasy and thereby mechanisms of adaptive evolution [16, 22–25]. Previous *H. pylori* genome analysis identified many positively selected genes revealing global footprints of adaptive events [26]. However, to date, no genome-wide studies have been reported of the role of convergent mutation in genetic homoplasy and adaptive evolution of *H. pylori* or other naturally-competent bacterial species. We show here that convergent mutations accumulate at high rates across *H. pylori* genomes under strong positive selection and independently from recombination, thereby constituting the major contributor to genetic homoplasy. Importantly, we find that the homoplastic convergence is extensively overlapped by the non-homoplastic convergent mutations (repeated non-identical changes at the same position), indicating convergent mutations as a significant driver of *H. pylori* adaptive evolution.

## Results

### High rate of homoplastic nonsynonymous substitution in *H. pylori* core genes

**Fig. 1:**
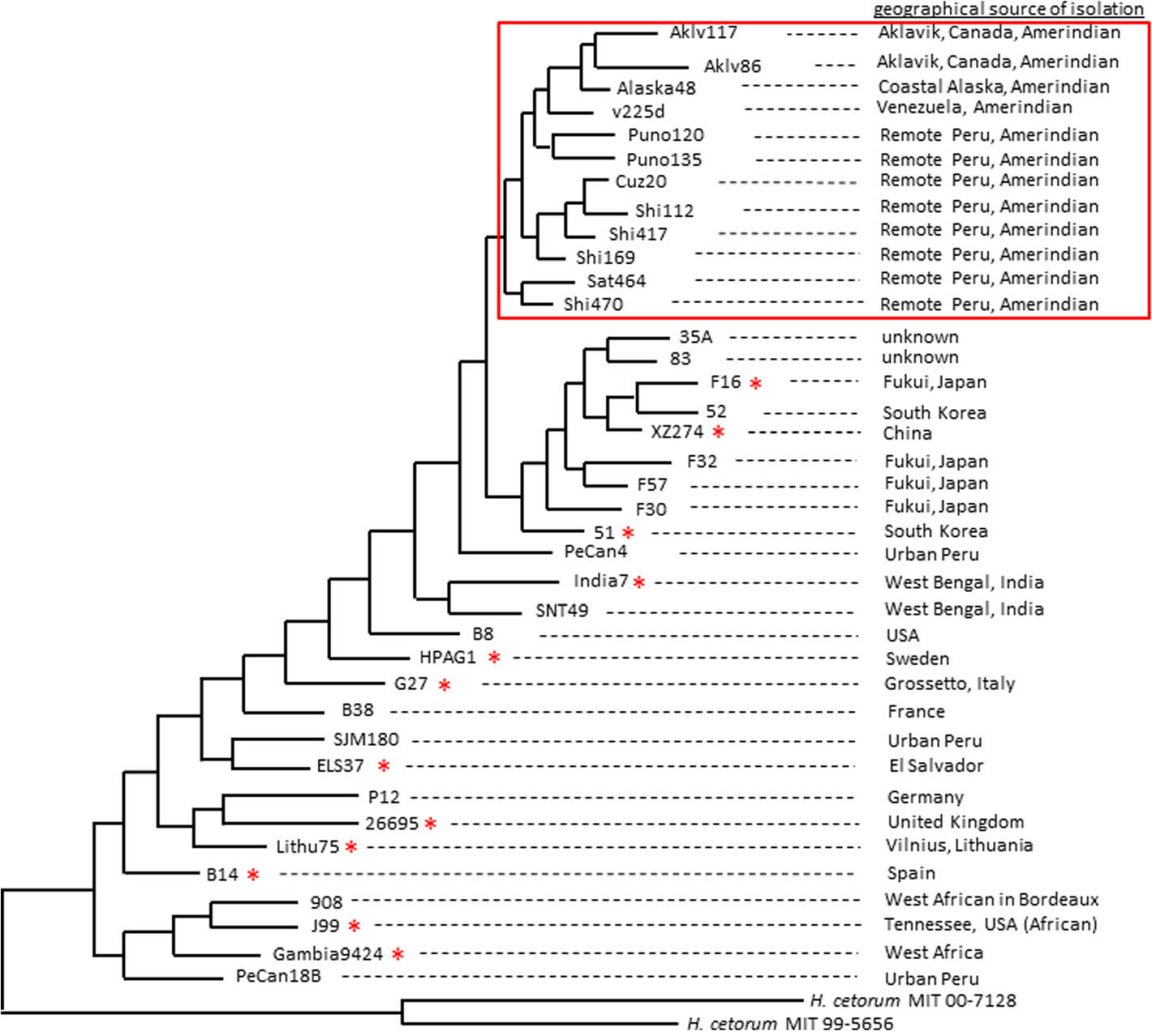
Phylogram of concatenated sequences of 7 housekeeping genes (*atpA*, *efp*, *mutY*, *ppa*, *trpC*, *ureI*, *yphC*) for 38 *H. pylori* strains analyzed, along with two *H. cetorum* strains used as outgroups. The red renctangle includes the strains from Amerindian population (representating the ‘local’ subset analyzed in Additional file 2: Figure S1). The red asterisks (*) beside the strain-names denote the representatives of the ‘global’ subset (analyzed in Additional file 2: Figure S1)

We analyzed protein-coding genes from 38 *H. pylori* strains that were chosen based on multi-locus sequence typing (MLST; http://pubmlst.org/helicobacter/) profiles (Fig. 1). Two *Helicobacter cetorum* strains (MIT 00–7128 and MIT 99–5656) were used as outgroups in reconstructing the phylogeny. Of 1566 protein-coding genes annotated in the genome of reference strain 26,695, 992 genes (63%) were present in all 38 strains, based on ≥90% threshold of nucleotide sequence-identity and gene length-coverage (Table 1). The average pairwise nucleotide diversity (π) of these core *H. pylori* genes was 3.81 ± 0.03%, with about 10-fold more synonymous (silent) than nonsynonymous (amino acid replacement) substitutions on average (Table 1). Using extracted protein sequences of these 992 core genes, we determined the extent of homoplasy in amino acid sequence differences, i.e. identical changes repeatedly found in the same protein positions in different strains that were not linked by common ancestry. Almost every core gene (98%, Table 1) showed homoplasy in their encoded proteins, with 8% positions per protein on average being homoplastic.

**Table 1:** Comparison of nucleotide diversity in core genes and homoplasy in the encoded proteins of *H. pylori*

	Complete set (38 strains)	‘Local’ (Amerindian) subset (12 strains)	‘Global’ subset (12 strains)
core genes analyzed	992	1106	1200
diversity, π	3.81 ± 0.03%	2.25 ± 0.02%	4.21 ± 0.03%
dN	1.44 ± 0.03%	0.91 ± 0.02%	1.74 ± 0.04%
dS	14.14 ± 0.11%	7.79 ± 0.08%	15.23 ± 0.13%
genes showing homoplasy in encoded proteins	971	963	1126
amino acid positions with repeated changes	7.85 ± 0.15%	2.33 ± 0.06%	5.10 ± 0.11%
recombinant genes	488	258	490
amino acid positions with repeated changes in encoded proteins of recombinant genes	9.03 ± 0.20%	2.83 ± 0.12%	6.02 ± 0.18%
non-recombinant genes	504	848	710
amino acid positions with repeated changes in encoded proteins of non-recombinant genes	6.66 ± 0.21%	2.15 ± 0.07%	4.40 ± 0.13%

The mean values are denoted in percentages along with their standard error values

Our dataset included 12 strains from Amerindians (both North and South America) and 26 strains from diverse other human populations worldwide. As expected, among the closely related Amerindian strains (boxed in red rectangle, Fig. 1), the average pairwise nucleotide diversity (π) of the core *H. pylori* genes was significantly lower than the diversity of the entire strains set, at both nonsynonymous and synonymous level (‘local’ subset, Table 1). For comparison, we selected from the remaining strains another subset of 12 non-Amerindian strains showing worldwide distribution (marked by asterisks, Fig. 1). The diversity of core genes in this subset (‘global’ subset, Table 1) was higher than the complete set, and almost twice that of the local dataset. Despite the difference in nucleotide diversity between the subsets, the vast majority of genes were affected by nonsynonymous homoplasy either in local or in global 12 strain subsets, and also in the complete 38 strain set. As expected, the average number of amino acid positions affected by homoplasy in the two 12 strain subsets was directly correlated with the overall nucleotide diversity level. The global subset showed two-fold more repeated identical amino acid substitutions than the local Amerindian subset. A selection of 5 additional sets of 12 strains from our non-Amerindian worldwide strain group showed an identical trend of global subset values relative to the local Amerinidian subset (Additional file 1: Table S1). However, the complete set had the highest frequency of amino acid homoplasy, in accord with its much larger size and higher diversity (Table 1). Thus, vast majority of genes in *H. pylori* is affected by high frequency nonsynonymous homoplasy, evident in either closely related or phylogenetically diverse collections, correlated with overall nucleotide diversity.

### Effect of recombination on the rate of nonsynonymous homoplasy

Homoplasy could result from homologous recombination between divergent strains, a traditional interpretation, given *H. pylori*’s natural competence, and possibilities for infection by multiple strains, at least transiently [8, 27, 28]. We applied PhiPack [29] to detect intra-genic recombination events across all core genes. This detected recombination in 49.2% of the genes with nonsynonymous homoplasy in the entire dataset, 40.8% in the 12 strains from global datasets, and 23.3% in the generally more closely related local, Amerindian dataset. However, when PhiPack analysis was applied to continuous regions of 2, 3, 5 and 10 genes (rather than just to individual genes) the frequency of recombination events detected increased exponentially and, at a certain point the frequency became higher in the local set than in the global one (Additional file 2: Figure S1). But, by extending the analysis from single to multiple genes the likelihood of detecting recombination could be higher. To verify if this was the situation at least for a continuous region of 2 or 3 genes, we chose a bin of 1000-1999 bp long sequences for single gene, 2-gene and 3-gene datasets containing 347, 252 and 65 sequences respectively. Also, the nucleotide diversity for the single gene set (π = 0.039 ± 0.0004) was almost the same for either the 2-gene dataset (π = 0.039 ± 0.0005) or 3-gene dataset (π = 0.037 ± 0.001). We detected 243 genes (70%) as recombinant in the single gene set of higher lengths, which was higher than the overall frequency of genes with intra-genic recombination (49%). However, in comparison, the 2- and 3-gene datasets of similar lengths and diversity values showed 194 genes (77%) and 55 genes (85%) respectively as recombinants. Thus, intra-genic recombination appears to be less frequent than inter-genic recombination in *H. pylori*, while the members of Amerindian subpopulations might be exposed to increased opportunities for recombination owing to geographical structure as suggested previously [30].

The rates of nonsynonymous homoplasy were re-evaluated after PhiPack analysis-based separation of genes into recombinant and non-recombinant. The average number of amino acid positions affected by repeated identical changes was 1.4-fold higher in recombinant than non-recombinant genes (9.03 ± 0.20% vs. 6.66 ± 0.21% positions, respectively, *P* < 0.0001) (Table 1). The same pattern of rather modestly high rates of nonsynonymous homoplasy in recombinant vs. non-recombinant genes were also observed in the local and global datasets (Table 1). We infer that nonsynonymous homoplasy is only partially due to intra-genic recombination events.

### Most repeated nonsynonymous changes are not linked to repeated synonymous changes

We used a recently developed statistic tool synDss [31] to assess linkage between repeated nonsynonymous and synonymous changes, an expected result of genetic recombination. Unlike the alignment score-based PhiPack, synDss separately computes synonymous and nonsynonymous codon distances within each sliding window for a given gene (by computing two types of phylogenetic incongruence based on synonymous-only and nonsynonymous-only substitution information) to test for recombination in intra-genic regions. Because of this tool’s computationally-intensive nature, we focused on randomly selected 25 recombinant and 25 non-recombinant genes as defined by PhiPack (Additional file 1: Table S2 and Additional file 2: Figure S2).

Overall, in all 50 genes combined, synDss detected 27 genes that crossed respective 95% bootstrap significance thresholds for phylogenetic incongruence, thereby suggesting clusters of repeated phylogenetically-unlinked changes – 17 in recombinant and 10 in non-recombinant sets (Fisher’s exact test two-tailed *P* = 0.088). Only 6 genes crossed the significance thresholds for both nonsynonymous and synonymous changes, and all of them represented PhiPack-defined recombinant set (Fisher’s exact test two-tailed *P* = 0.03). Interestingly, 17 genes showed significance due to nonsynonymous substitutions only, which was much higher than genes showing significant values for both nonsynonymous and synonymous substitutions (6 genes; Fisher’s exact test two-tailed *P* = 0.016) and, especially, for synonymous substitutions only (4 genes; Fisher’s exact test two-tailed *P* = 0.003). However, between the PhiPack-based recombinant and non-recombinant gene sets, there was no statistical difference in the number of genes showing significance for nonsynonymous changes only (10 and 7 genes, respectively, Fisher’s exact test two-tailed *P* = 0.55) or for synonymous changes only (1 and 3 genes, respectively, Fisher’s exact test two-tailed *P* = 0.61). Multiple testing correction using the Benjamini-Hochberg method [32] showed that the significant *P* values fell below the 5% false discovery rate.

One possible explanation for a large number of cases showing significant phylogenetic incongruence for nonsynonymous homoplasy only could be a lack of recombination detection power in the synonymous substitution analysis. However, such an explanation is unlikely since the observed rate of synonymous substitutions is way higher than the nonsynonymous rate in *H. pylori* (Table 1). Importantly, in gene phylogenies the corresponding amino acid homoplastic sites were not clustered in any single allele-pairs, thereby ruling out the possibility that recombination happened in an extreme situation free of synonymous changes. Therefore, although synDss partially validated that some of *H. pylori*’s nonsynonymous homoplasy is of recombinant origin, the majority of repeated nonsynonymous changes are not linked to repeated synonymous changes, and thus are not likely to stem from intra-genic recombination.

### Prevalence of convergent mutations in proteins with homoplastic changes

Homoplastic (i.e. repeated identical) changes constitute a type of convergent change that, taken alone, could be attributed to recombination. Another type involves repeated non-identical (i.e. non-homoplastic) changes at the same positions, and represents unambiguous evidence of convergence by mutation. We examined whether amino acid positions with homoplasy were also targeted by non-homoplastic convergence. For this further homoplasy analysis, we concentrated on the PhiPack-defined set of 504 non-recombinant genes, to reduce possible ambiguity in the results.

**Fig. 2:**
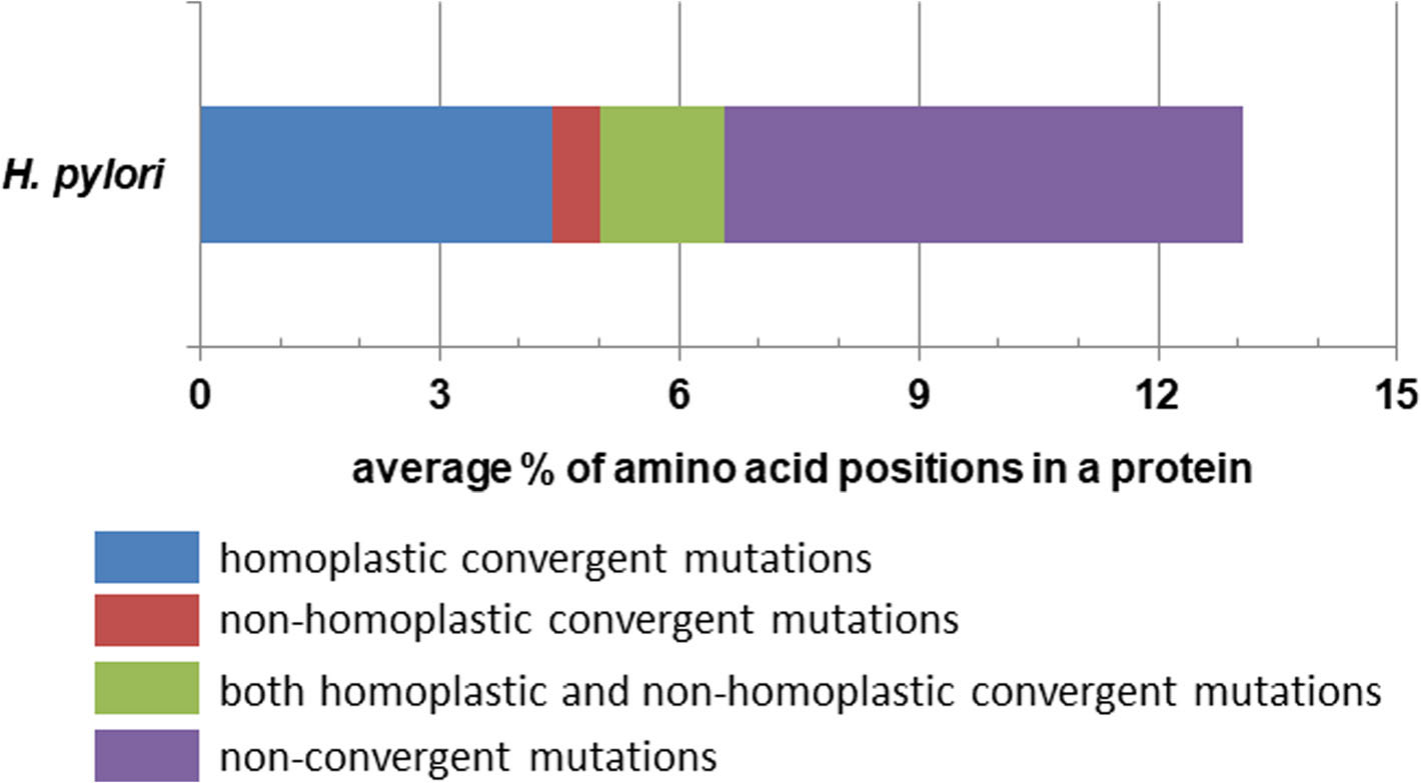
Average distribution of amino acid positions with different kinds of point mutations in encoded proteins of non-recombinant core genes with convergent changes in *H. pylori*

**Fig. 3:**
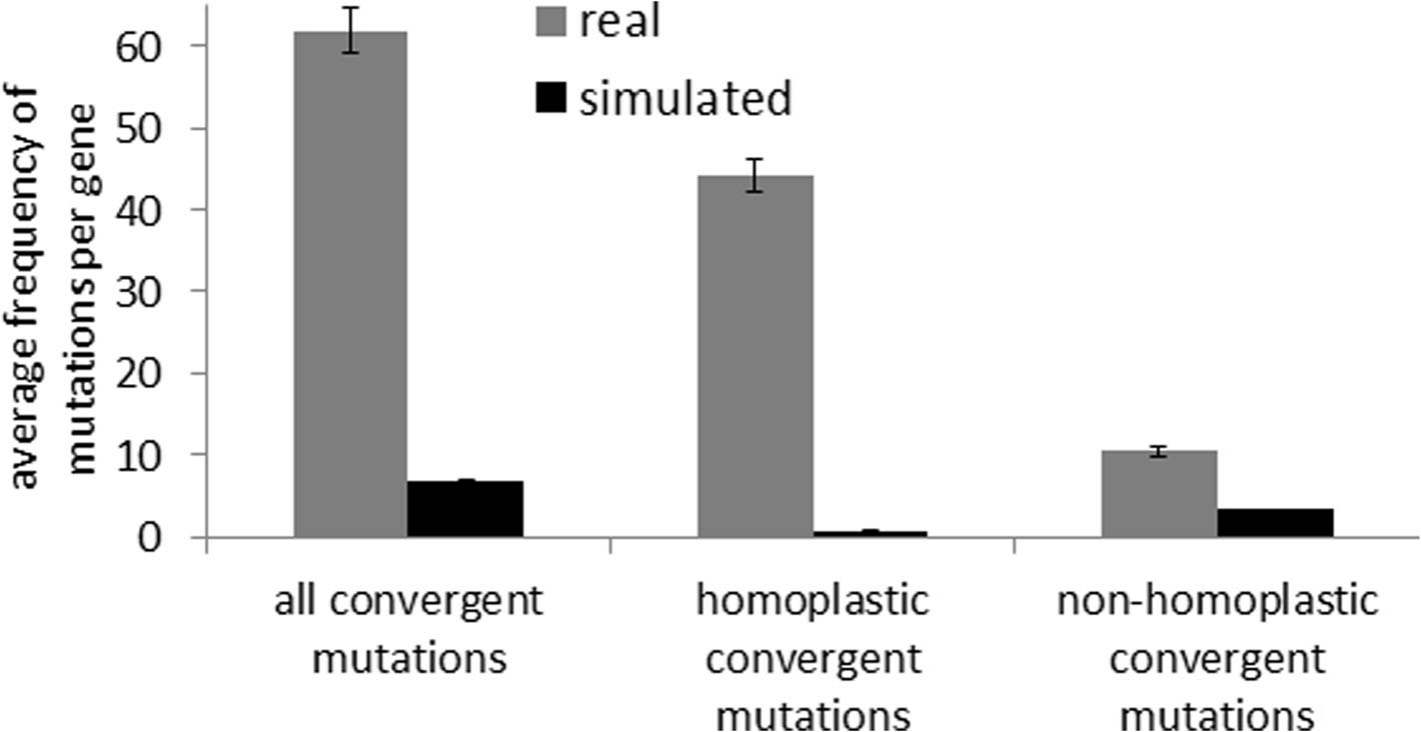
Comparison of convergent mutation-frequencies in encoded proteins of real and simulated datasets of *H. pylori* non-recombinant core genes

We found a high frequency of repeated but non-identical (i.e. non-homoplastic convergent) mutations in proteins encoded by these 504 core *H. pylori* genes (Fig. 2). Interestingly, 73% of positions with non-homoplastic convergent mutations were also targeted by homoplastic changes in our 38 genome dataset, and the frequency of homoplastic changes was higher than that of non-homoplastic ones. To determine whether the observed pattern could be explained by random mutation accumulation under no selection, for each gene we performed simulation of mutations under neutrality, based on the naturally observed mutation rate. We found that the frequency of non-homoplastic (non-identical) amino acid convergence was > 3 times higher in the real datasets than in simulated ones; in addition, the difference in frequency of homoplastic (identical) amino acid convergence was > 50 times higher in the real datasets than in the simulated ones (Fig. 3), suggesting non-neutral accumulation of convergent changes (*P* < 0.0001). Also, as expected under neutrality, the simulated data showed higher frequency of non-homoplastic amino acid changes than the homoplastic ones (Fig. 3), which contrasted with the observed prevalence of homoplastic changes in *H. pylori* genes.

**Fig. 4:**
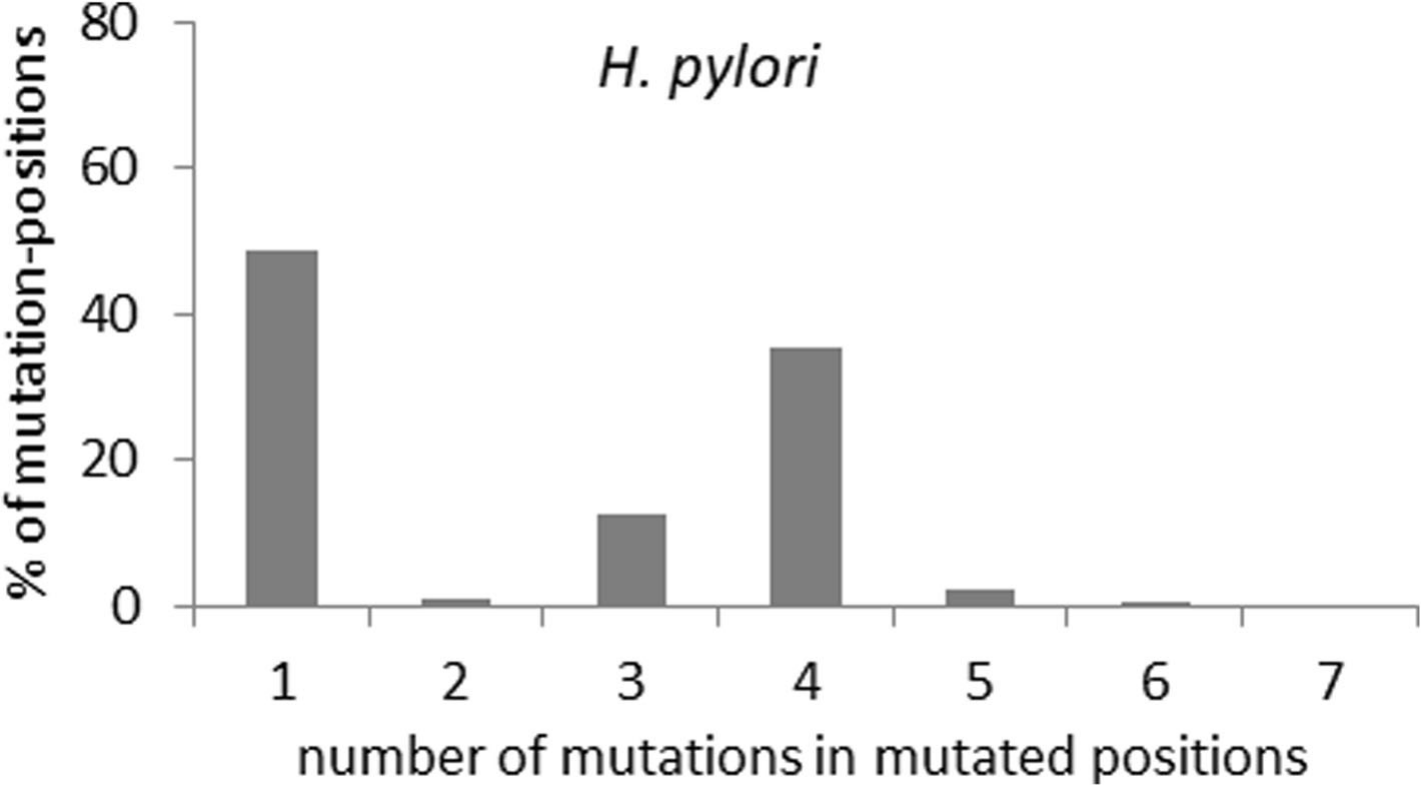
Average frequency distribution of mutations in the encoded proteins showing convergence in non-recombinant core genes of *H. pylori*

In principle, the high frequency of convergent mutations in particular positions might arise by chance alone due to *H. pylori*’s overall high mutation rate and the limited availability of mutable sites that tolerate changes without impairing function. To test this, we scored the average frequency of mutations per mutated site in encoded proteins, and found a bimodal distribution with two distinct peaks in frequencies – one with a single mutation position and the other with an average of four mutations per site, affected by multiple repeated changes of identical and/or non-identical (homoplastic or non-homoplastic convergent) nature (Fig. 4). We next analyzed the mutation frequency in 5 randomly selected replicates of 100, 50, 25 and 10 randomly chosen core genes (Additional file 2: Figure S3). We found that the bimodal mutation frequency distribution was also preserved in the 100, 50 and 25 member smaller subsets (such bimodality was not evident in the 10 gene subset due to small sample size noisiness) (Additional file 2: Figure S3). Thus, the bimodal distribution across the genome is not attributable to a skewed mutation distribution in a limited gene set.

We next performed a simulation analysis of one random set of 25 genes, using two sets of mutation rates and dN/dS values: (a) as observed in the *H. pylori* genes, and (b) two-fold higher than the values observed in the *H. pylori* genes. In both cases, the distributions of amino acid mutation frequencies in the simulated datasets were unimodal, not bimodal as observed for real datasets, with the probabilities of multiple mutations per site dropping progressively (Additional file 2: Figure S4). Taken together, these results demonstrate that homoplastic and non-homoplastic convergence strongly overlap, and that the occurrence of convergent nonsynonymous changes in *H. pylori* non-recombinant genes cannot be explained by neutral accumulation of mutations. This suggests underlying action of strong positive selection for convergent evolution.

### Diverse array of enriched functional categories

**Fig. 5:**
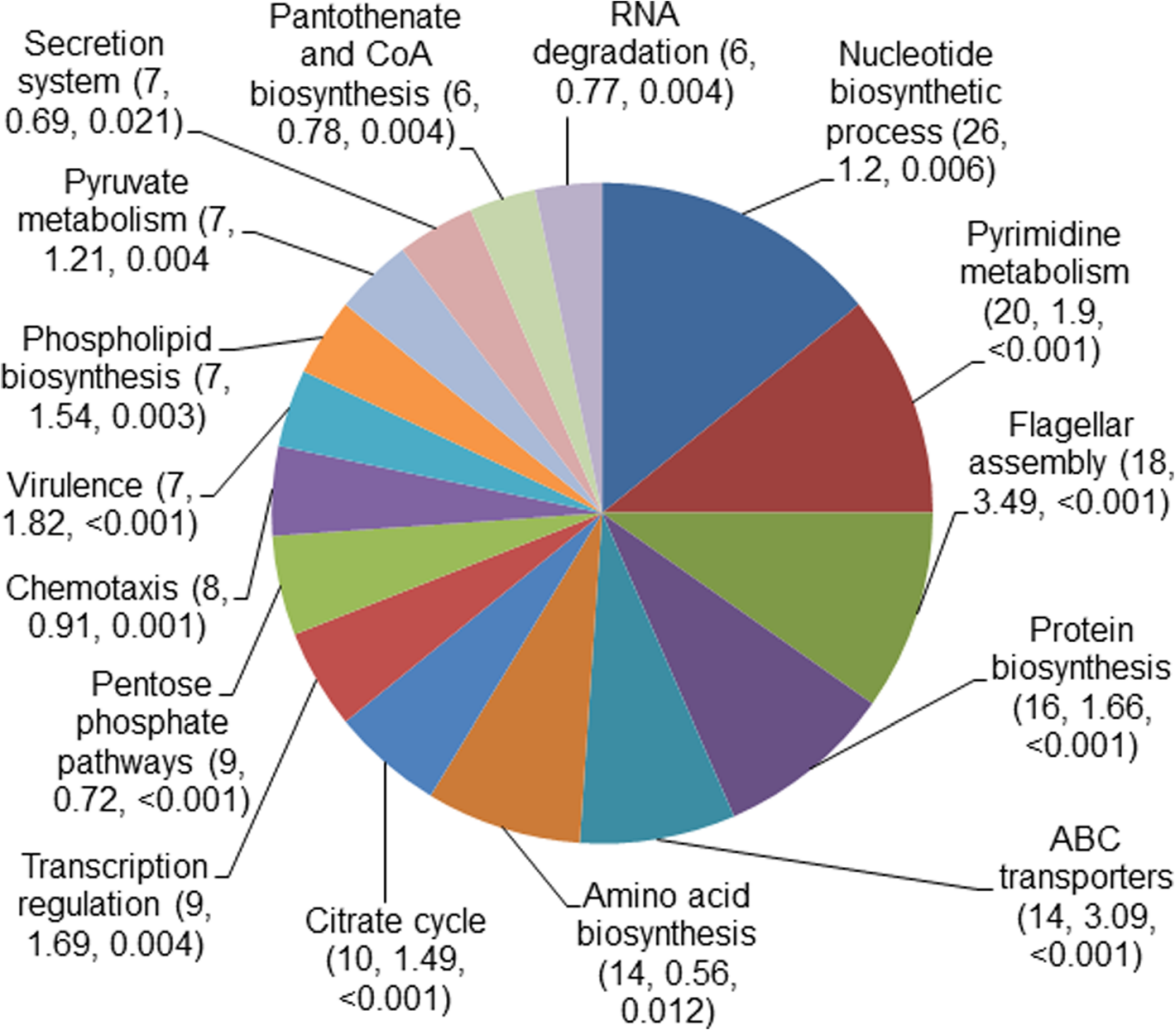
Enriched (or overrepresented) functional categories of proteins encoded by non-recombinant core genes with convergent mutations

The high rate of acquisition of convergent mutations in *H. pylori* suggests robust positive selection in the affected genes (Additional file 1: Table S3). Of the 487 non-recombinant genes with potential convergent mutations, 158 (32%) were in 16 biological process categories that were statistically enriched or over-represented (*P* < 0.05) in the dataset out of a total of 67 categories (Fig. 5). These included genes involved in protein secretion, chemotaxis, transport, virulence, flagellar assembly, RNA degradation, transcriptional regulation, and also several metabolic or biosynthetic pathways. Importantly, multiple genes in each of these pathways accumulated convergent mutations across different *H. pylori* lineages. Notably, we did not find enrichment of genes representing some of the functional categories commonly over-represented in *E. coli* or *Salmonella* [20], such as two-component systems, DNA repair, or biosynthetic pathways of cofactors, vitamins, quinones, carbohydrates, etc. Thus, *H. pylori* proteins affected by homoplastic changes that likely stem from convergent evolution are not distributed randomly among functional categories. This implies that many of the observed protein sequence changes have contributed to *H. pylori*’s adaptation to particular gastric mucosal environments.

## Discussion

Here we provide a genome-wide analysis of *H. pylori* strains that shows that, independent of recombination, convergent evolution by mutation and selection contributes importantly to extensive homoplasy in the core genes of this gastric pathogen. Such a high frequency of homoplasy, we believe, would considerably impact the tree topology based on all core genes. Instead, we reconstructed phylogeny of the analyzed strains based on the MLST loci to have only an overview of clonal grouping and diversity, since a phylogenomic or phylogeographic study was not the focus of our work. The MLST loci-based phylogram, using two *Helicobacter cetorum* strains as outgroups, displayed mainstream African, East Asian and Amerindian strains in their own distinct groups. Interestingly, the clustering of an urban Peruvian strain PeCan18B with the African strains could be attributed to the history of African slavery in Peru (and much of Latin America) during colonial times, a significant contribution of genes of ethnic Black Africans to the Lima region human gene pool, and distinct self-identified Afro-Peruvian communities in Lima and elsewhere in the country [33].

Convergent mutations at specific amino acid positions are mostly selected under positive selection, for adaptive or compensatory functions [19–21, 34–40]. The possible structural and functional reasons for accumulation of mutations at particular sites are manifold. The adaptive significance of convergent mutations in proteins, especially of the non-homoplastic ones, could stem from selective pressures on sets of functionally/structurally critical amino acid residues to avoid immune system epitope recognition or alter or even eliminate protein function [40]. Also, homoplastic mutations, in particular, suggest positive selection for fine-tuned directed changes in protein function [34].

Our conclusion about the non-recombinant nature of many or most homoplastic *H. pylori* gene changes is based primarily on the abundance of non-homoplastic convergent mutations and their significant overlap with homoplastic variations in *H. pylori* genes. Notably, it is not based just on a possible failure of recombination-detection tools to establish a strong link between structural homoplasy and recombination (which are probability-based, and some may need further improvement or might be biased towards assuming a priori that homoplasy is recombinant in nature). There is always a possibility that an excessively high frequency of adaptive nonsynonymous homoplasy in localized regions could be of recombinant origin, while a low frequency of synonymous homoplasy around the regions could be due to a lack of positive selection to be either introduced or maintained by recombination. Such a scenario would easily arise if the recombination tract lengths were very short, in the order of tens of nucleotides. However, in light of previously reported large lengths of imported fragments [10–12] discussed below and consistent high rate of synonymous changes (10 times higher on an average than the rate of nonsynonymous changes), nonsynonymous homoplasy without a linked synonymous homoplasy would require a large number of positionally constrained recombination events around the former type of changes. Thus, we consider the ‘stand-alone’ nonsynonymous homoplasy to be more plausibly (parsimoniously) explained via mutation rather than recombination, at least for a substantial part of them. Additional argument for the mutational origin of homoplasy is, if course, the overlap of both homoplastic and non-homoplastic types of change in the affected nucleotide positions.

That said, we note that our study does not diminish the adaptive importance of frequent homologous recombination in *H. pylori*. Certainly a portion of the amino acid homoplasy assigned here as likely due to mutation actually stems from recombination. Laboratory and sequence analyses of length of imported fragments in *H. pylori* genomes indicated that most recombination in *H. pylori* is inter- rather than intra-genic nature. The mean length of *H. pylori* core genes is 959 bp (95% in range of 922–996 bp). According to two recent studies, the mean length of DNA transferred from donor to recipient in vivo by a single recombination event was estimated as about 1300 bp and 1247 bp (95% confidence intervals of 950-1850 bp and 841–1721 bp, respectively) [11, 12] . Another in vitro transformation study reported an average length of imported fragments varied from 1294 to 3853 bp [10]. Finally, an additional study [41] reported an average recombination tract length of 895 bp based on two lineages of very closely related isolates from a single patient. The above ranges of recombination tract lengths were considerably higher than the value of 417 bp detected previously among serial isolates [8]. However, a follow-up study from the same group had increased the estimated recombination length three fold [12] and attributed such a discrepancy to limited number of recombination events in the earlier study. This suggests that recombination, under selection, might be primarily responsible for exchanges of relatively large regions (likely exceeding the length of a single gene), rather than for swapping of single nucleotide polymorphisms or very small regions. Thus, if recombination would be responsible for most ‘stand-alone’ single nucleotide homoplasy in *H. pylori*, this would require a fairly large number of sequential, partially-overlapping recombination events within the immediate proximity a specific nucleotide position. While this is possible, a mutational origin of the widespread single nucleotide homoplasy appears to be a more parsimonious explanation. Therefore, in individual gene-based analysis, homoplastic changes owing to inter-genic recombination would most likely be phylogenetically linked and not appear as independent repeated substitutions in our analysis.

Interestingly, *H. pylori* shows a bi-modal distribution of mutation-frequencies, which could be an intrinsic characteristic of selective forces operating on proteins in this fast-evolving organism. This suggests a strong bias for multiple mutations at the same amino acid positions. But our simulations under neutrality do not support a possibility of saturation of mutable amino acid residues even at a mutation rate twice that of *H. pylori*. Although the simulation algorithm involved limitations due to simplified assumptions, our goal was to decipher if a simulation model under neutrality could at all explain the observed trend of the high frequency of homoplastic and non-homoplastic convergence, or bimodality of the mutation frequency distribution. Also, we believe that, while the lack of accuracy of the reconstructed tree topologies compromises the detection level of homoplasy events, such a scenario would equally affect the detection of nonsynonymous and synonymous homoplasy. Therefore, the findings of unlinked nature of nonsynonymous homoplasy from the synonymous ones, along with its overlap with the nonsynonymous non-homoplastic convergent mutations would mostly remain true. However, to validate these results and to decipher the detailed evolutionary history of lineages, a population-level genomic study is warranted which is out of scope of the present work. Importantly, the diversity of human gastric mucosal micro-niches for *H. pylori* is very limited, especially relative to organisms like *Escherichia coli* or *Salmonella enterica*, which have a much broader host and environmental range. Thus, the affected proteins may be constrained to only a limited set of residues that could be under strong selection-pressures to tackle highly specific environmental challenges. Interestingly, the proportion of non-convergent mutation sites is lower than the convergent ones in *H. pylori*. This indicates that the effective number of evolvable sites that respond to selection pressures might have reached a saturation point, while the frequency of positions that can carry mutations via neutral drift is possibly far from saturation.

An overall low dN/dS value with high homoplasy rates in *H. pylori* genes could indicate mutational site saturation for nonsynonymous positions and strong purifying selection as suggested previously [24]. However, we believe that single nucleotide homoplasy in *H. pylori* is positively selected that could be explained by either adaptive or compensatory nature of the convergent mutations. The adaptive mutations could provide fitness diversification in a highly variable and hostile environment encountered by *H. pylori* during colonization of the same and different individuals. This is evident from the overlap of both homoplastic (to possibly achieve a directed, fine-tuned fitness level) and non-homoplastic (to possibly offer evasion of host defenses) changes in majority of the positions with convergent mutations. On another hand, the excessively high rate of mutations could include, along with the beneficial ones, a significant amount of deleterious mutations. High rates of compensatory mutations (along with recombination) might compensate for disruptions in fitness by adjusting structure-function relationships within a single protein or between different proteins.

Maintenance of extensive genomic diversity in *H. pylori* via a high level of homoplastic and non-homoplastic convergence suggests a possible interplay of adaptive and compensatory roles of the changes to cope with extremely hostile micro-niches. For example, earlier works on *E. coli* [42] and *H. pylori* [43] strains have demonstrated that the acquisition of adaptive antibiotic resistance mutations is balanced by additional mutations that compensate for the biological cost of resistance, which thereby can lead to increased fitness and stabilization of resistant mutants in the population. To cope with broad classes of environmental conditions (ranging from within-host micro-niches to hosts diversified by geography, genotypes, dietary habits, etc.), it is likely that mutational convergence in *H. pylori* is distributed across a wide range of proteins from different functional categories. Indeed, functional classification showed that the genes with convergent mutations represented a diverse group of functional categories. Kawai et al. [44] earlier detected seven genes where the encoded proteins accumulated positively selected amino acid changes. However, five of them were non-core in our dataset. The remaining two core genes were found to be recombinants, and therefore not considered as candidates. Recently, Montano et al. [26] demonstrated global footprints of selection in *H. pylori*, identifying an array of genes to be under positive selection in worldwide population or in different local (African, EuroAsian, East Asian, or American) sub-populations. Fifty-three of their listed selection candidates were included in the core gene dataset we analyzed here. Our PhiPack analysis identified 28 of these genes as non-recombinant. All these genes were selection candidates in our list as well owing to their accumulation of convergent mutations (marked by asterisks in Additional file 1: Table S3). Similarly, another recent work by Yahara et al. [45] offered a list of 134 *H. pylori* genes with positively selected amino acid changes. We found 23 of these genes to be non-recombinant core in our dataset, all of which were detected as candidate genes (marked by ‘+’ in Additional file 1: Table S3). However, it was not surprising given the fact that 97% of non-recombinant genes in our dataset were selected as candidates for the accumulation of convergent mutations – homoplastic and/or non-homoplastic. Nevertheless, this listing of candidate genes under positive selection and naturally occurring convergent mutations (Additional file 1: Table S3) should encourage further studies to understand functional implications of the many mutations found here.

## Conclusions

Our study highlights the genome-wide prevalence of evolutionarily convergent mutations and possible (patho-)adaptive roles in one of the most common and fast-evolving bacteria species. We hypothesize that the selection-driven convergent mutational dynamics detected in *H. pylori* is also characteristic of the evolution of other naturally competent bacterial species like *Neisseria* [46], *Streptococcus* [47], etc. Future large-scale population-wide analysis of the hundreds of sequenced *H. pylori* genomes now available will further characterize mutational footprints across isolates from both geographically diverse and localized populations, and thereby further identify sequence changes linked to within-host micro-niche adaptation, associations with distinct disease phenotypes, human genotypes, dietary habits and geographical locations [48–51].

## Methods

### Reconstruction of MLST phylogeny

The maximum-likelihood based phylogenetic tree of 38 completely sequenced *H. pylori* genomes was reconstructed using concatenated sequences of 7 housekeeping genes – *atpA, efp, mutY, ppa, trpC, ureI, yphC* (http://pubmlst.org/helicobacter/). The maximum-likelihood based phylogenetic trees for MLST and all individual genes in this study were reconstructed using the general time reversible (GTR) substitution model with estimated base frequencies site-specific by codon position distribution as implemented in PAUP* [52].

### Extraction, phylogenetic reconstruction, homoplasy detection and diversity analysis of core genes

The TimeZone software package [53] was used to extract core protein-coding genes, excluding the annotated pseudogenes, from the genome of *H. pylori* strain 26695 used as reference. Stand-alone BLAST, implemented in TimeZone, was used to find orthologs of each gene from the genomes of other *H. pylori* strains using 90% cut-off values for both nucleotide sequence identity and sequence length coverage. For each core gene, phylogenetic analysis of its allelic sequences was performed via TimeZone to detect homoplastic events in the encoded protein. The rates of synonymous (dS) and nonsynonymous (dN) mutations using mutation-fraction method of Nei and Gojobori [54], and average pairwise nucleotide diversity (π) were also calculated via TimeZone.

### Detection of recombination

We used the PhiPack software package [29] for detecting probable recombination events. This package included 3 recombination-detection statistics: pairwise homoplasy index (Phi), maximum χ^2^ (MaxChi), and neighbor similarity score (NSS). These statistics sequentially compare each set of all possible combinations of three sequences in the dataset, and perform sliding window analysis for the relative distribution of nucleotide polymorphisms. Presence of segments of the sequence alignment that support significantly different sequence diversity or sequence topology than the upstream and downstream segments is considered as evidence of intra-genic recombination. A gene was considered to be recombinant if *P* values for all of the 3 statistics were < 0.1 [55].

The synDss analysis [31] involved detection of phylogenetic incongruence based on synonymous and nonsynonymous evolutionary distances across multiple sliding windows within each given gene. The nucleotide distances were estimated on the codon level (i.e. the expected number of substitutions per site) using the window size and step size as tuning parameters, calibrated to have approximately 100 windows across each alignment. Statistical significance of the synDss statistic was computed by parametric bootstrapping of 500 replications under the null hypothesis of no recombination. The Monte Carlo estimate of the P value was achieved from this parametric bootstrap, and 95% bootstrap significance thresholds were shown for the synonymous and nonsynonymous plots (Additional file 2: Figure S2).

### Simulations

Random simulations of mutation were performed using EvolveAGene 3 [56]. For the simulation of each gene, the sequence of reference *H. pylori* strain 26695 was considered as the root sequence to generate a random tree topology where each branch had equal probability of leading either to a terminal node or to an internal node. To assign the mutation rate, average branch lengths and average selection on amino acid replacements (i.e., dN/dS) were estimated from the real data set phylogeny of corresponding gene for the simulations shown in Fig. 4. In contrast, for the ones in Additional file 2: Figure S4 (Additional file 2), a set of 25 sequences were simulated based on two different values of average branch-lengths. Since real data sets of core genes did not have any indels, no indels were allowed in the simulated data sets. Selection over sequence, as well as over branches along the tree, was set to be constant with the default modifier value of 1. Random selection of gene subsets was performed by in-house random number generator program.

### Functional enrichment analysis

DAVID software [57] was applied to perform functional annotation clustering using DAVID annotation of *H. pylori* genes as reference. For the analysis, classification stringency was set to ‘medium’. To minimize redundancy, annotated clusters representing only Biological Process category of Gene Ontology (GO) were considered. The functional categories with enrichment score > 0.5 and *P* value < 0.05 were selected as the enriched or overrepresented ones.

## Additional files

Additional file 1: **Table S1.** Comparison of nucleotide diversity in core genes and homoplasy in the encoded proteins of analyzed ‘Global’ subset of *H. pylori* with 5 other global subsets based on random selection of 12 non-Amerindian strains in our dataset. The mean values are denoted in percentages along with their standard error values. **Table S2.** Results of 95% significance level (based on a parametric bootstrap with *B* = 500) of synonymous and non-synonymous synDss statistics for 50 randomly selected core genes of *H. pylori* – 25 recombinant and 25 non-recombinant as defined using PhiPack analysis. **Table S3.** List of 487 non-recombinant core genes under positive selection for accumulating convergent amino acid mutations in the encoded proteins. The genes with ‘*’ and ‘+’ denote the positively selected genes as detected by Montano et al. [26] and Yahara et al. [45] respectively. (PDF 729 kb)

Additional file 2: **Figure S1.** Frequency of recombinant regions as detected by PhiPack using single (intra-genic) vs. multiple (inter-genic) genes in ‘global’ subset and ‘local’ (Amerindian) subset of strains (as denoted in Fig. 1). **Figure S2.** The synDss statistic landscapes for 50 randomly selected core genes of *H. pylori*: (A) 25 recombinant and (B) 25 non-recombinant, as designated by three recombination detection statistics in PhiPack software. **Figure S3.** Average frequency distribution of mutations in encoded proteins of randomly selected subsets of *H. pylori* non-recombinant core genes with convergent mutations. **Figure S4.** Average frequency distribution of mutations of encoded proteins in simulated datasets of 25 *H. pylori* genes (one of the randomly selected replicates from **Figure S3**) using two different mutation-rate constraints. (PDF 2545 kb)

## Abbreviations

GO: Gene ontology
GTR: General time reversible
MaxChi: Maximum χ^2^
MLST: Multi-locus sequence typing
NSS: Neighbor similarity score
Phi: Pairwise homoplasy index
