# Supplementary material for: Recombination-independent rapid convergent evolution of the gastric pathogen Helicobacter pylori
Source: BMC Genomics. 2018 Nov 21;19:835. doi: 10.1186/s12864-018-5231-7 (PMC6249973; doi:10.1186/s12864-018-5231-7)
Supplement: Supplementary file 1 — Table S1. Comparison of nucleotide diversity in core genes and homoplasy in the encoded proteins of analyzed ‘Global’ subset of H. pylori with 5 other global subsets based on random selection of 12 non-Amerindian strains in our dataset. The mean values are denoted in percentages along with their standard error values. Table S2. Results of 95% significance level (based on a parametric bootstrap with B = 500) of synonymous and non-synonymous synDss statistics for 50 randomly selected core genes of H. pylori – 25 recombinant and 25 non-recombinant as defined using PhiPack analysis. Table S3. List of 487 non-recombinant core genes under positive selection for accumulating convergent amino acid mutations in the encoded proteins. The genes with ‘*’ and ‘+’ denote the positively selected genes as detected by Montano et al. [26] and Yahara et al. [45] respectively. (PDF 729 kb) [file 12864_2018_5231_MOESM1_ESM.pdf]

Table S1. Comparison of nucleotide diversity in core genes and homoplasy in the encoded proteins of analyzed 'Global' subset of *H. pylori* with 5 other global subsets based on random selection of 12 non-Amerindian strains in our dataset. The mean values are denoted in percentages along with their standard error values.

	'Global' subset (12 strains)	SET 1	SET 2	SET 3	SET 4	SET 5
core genes analyzed	1200	941	984	926	936	988
diversity, π	4.21±0.03 %	4.10±0.04 %	4.08±0.03 %	4.12±0.04 %	4.13±0.03 %	3.84±0.03 %
dN	1.74±0.04 %	1.49±0.03 %	1.46±0.03 %	1.47±0.03 %	1.49±0.03 %	1.37±0.03 %
dS	15.23±0.13 %	15.47±0.12 %	15.52±0.11 %	15.68±0.12 %	15.77±0.11 %	14.48±0.11
genes showing homoplasy in encoded proteins	1126	846	891	832	826	886
amino acid positions with repeated changes	5.10±0.11 %	4.36±0.09 %	4.24±0.09 %	4.48±0.12 %	3.97±0.09 %	4.43±0.10 %
recombinant genes	490	344	357	345	291	415
amino acid positions with repeated changes in encoded proteins of recombinant genes	6.02±0.18 %	5.08±0.16 %	4.82±0.15 %	5.04±0.15 %	4.73±0.16 %	5.04±0.15 %
non-recombinant genes	710	597	627	581	645	573
amino acid positions with repeated changes in encoded proteins of non-recombinant genes	4.40±0.13 %	3.34±0.10 %	3.38±0.10 %	3.52±0.16 %	3.07±0.09 %	3.27±0.12 %

Table S2. Results of 95% significance level (based on a parametric bootstrap with *B* = 500) of synonymous and non-synonymous synDss statistics for 50 randomly selected core genes of *H. pylori* – 25 recombinant and 25 non-recombinant as defined using PhiPack analysis.

**Gene**	**Product**	**Protein-length (amino acid)**	**Synonymous Dss**	**Non-synonymous Dss**	**Possible indication**
25 randomly selected core genes designated as **recombinants** by PhiPack					
HP_0029	dethiobiotin synthetase (*bioD*)	218	no	no	neither
HP_0042	*trbI* protein	233	yes	no	synonymous convergence
HP_0102	conserved hypothetical protein	259	no	yes	non-synonymous convergence
HP_0150	predicted coding region HP0150	196	no	yes	non-synonymous convergence
HP_0214	sodium-dependent transporter (*huNaDC-1*)	552	no	yes	non-synonymous convergence
HP_0298	dipeptide ABC transporter, periplasmicdipeptide-binding protein (*dppA*)	549	yes	yes	recombination
HP_0352	flagellar motor switch protein (*fliG*)	343	no	yes	non-synonymous convergence

HP_0393	chemotaxis protein (*cheV*)	311	no	no	neither
HP_0493	phospho-N-acetylmuramoyl-pentapeptide-transferase (*mraY*)	353	no	yes	non-synonymous convergence
HP_0563	predicted coding region HP0563	416	yes	yes	recombination
HP_0603	predicted coding region HP0603	189	no	yes	non-synonymous convergence
HP_0630	modulator of drug activity (*mda66*)	194	yes	yes	recombination
HP_0683	UDP-N-acetylglucosamine pyrophosphorylase(*glmU*)	433	yes	yes	recombination
HP_0747	conserved hypothetical protein	393	no	no	neither
HP_0865	deoxyuridine 5'-triphosphate nucleotidohydrolase(*dut*)	145	no	no	neither
HP_0959	conserved hypothetical protein	243	no	yes	non-synonymous convergence
HP_1014	7-alpha-hydroxysteroid dehydrogenase (*hdhA*)	262	no	no	neither
HP_1029	predicted coding region HP1029	178	no	yes	non-synonymous convergence
HP_1055	predicted coding region HP1055	314	yes	yes	recombination

HP_1112	adenylosuccinate lyase (*purB*)	440	no	no	neither
HP_1121	cytosine specific DNA methyltransferase(BSP6IM)	312	no	no	neither
HP_1232	dihydropteroate synthase (folP)	380	yes	yes	recombination
HP_1268	NADH-ubiquinone oxidoreductase, NQO9 subunit(NQO9)	220	no	no	neither
HP_1508	ferrodoxin-like protein	458	no	yes	non-synonymous convergence
HP_1529	chromosomal replication initiator protein(*dnaA*)	457	no	yes	non-synonymous convergence
25 randomly selected core genes designated as **non-recombinants** by PhiPack					
HP_0020	carboxynorspermidine decarboxylase (*nspC*)	405	no	no	neither
HP_0124	translation initiation factor IF-3 (*infC*)	203	yes	no	synonymous convergence
HP_0146	cbb3-type cytochrome c oxidase subunit Q (*ccoQ*)	73	no	no	neither
HP_0192	fumarate reductase, flavoprotein subunit (*frdA*)	714	yes	no	synonymous convergence

HP_0232	secreted protein involved in flagellar motility	215	no	yes	non-synonymous convergence
HP_0274	conserved hypothetical protein	132	no	no	neither
HP_0299	dipeptide ABC transporter, permease protein(*dppB*)	334	no	no	neither
HP_0380	glutamate dehydrogenase (*gdhA*)	448	yes	no	synonymous convergence
HP_0420	predicted coding region HP0420	142	no	no	neither
HP_0736	phosphoserine aminotransferase (*serC*)	369	no	no	neither
HP_0742	phosphoribosylpyrophosphate synthetase (*prsA*)	318	no	yes	non-synonymous convergence
HP_0771	predicted coding region HP0771	245	no	no	neither
HP_0884	predicted coding region HP0884	614	no	yes	non-synonymous convergence
HP_0908	flagellar hook (*flgE*)	605	no	no	neither
HP_0933	predicted coding region HP0933	200	no	yes	non-synonymous convergence
HP_0950	acetyl-CoA carboxylase beta subunit (*accD*)	289	no	no	neither

HP_0958	predicted coding region HP0958	254	no	no	neither
HP_1016	phosphatidylglycerophosphate synthase (*pgsA*)	200	no	yes	non-synonymous convergence
HP_1050	homoserine kinase (*thrB*)	293	no	no	neither
HP_1190	histidyl-tRNA synthetase (*hisS*)	442	no	yes	non-synonymous convergence
HP_1203	transcription termination factor NusG (*nusG*)	176	no	no	neither
HP_1269	NADH-ubiquinone oxidoreductase, NQO10 subunit (NQO10)	182	no	no	neither
HP_1299	methionine amino peptidase (*map*)	253	no	yes	non-synonymous convergence
HP_1305	ribosomal protein S8 (*rps8*)	131	no	no	neither
HP_1559	flagellar basal-body rod protein (*flgB*)	140	no	no	neither

Table S3. List of 487 non-recombinant core genes under positive selection for accumulating convergent amino acid mutations in the encoded proteins. The genes with '*' and '+' denote the positively selected genes as detected by Montano et al. [26] and Yahara et al. [45] respectively.

**Gene**	**GI**	**CDS-region**	**Product**	**Protein Length (AA)**
HP_0001	2313078	220-633	predicted coding region HP0001	138
HP_0002	2313079	638-1105	riboflavin synthase beta chain (ribE)	156
HP_0003	2313080	1118-1945	3-deoxy-d-manno-octulosonic acid 8-phosphatesynthetase (kdsA)	276
HP_0005	2313082	2719-3399	orotidine 5'-phosphate decarboxylase (pyrF)	227
HP_0010	2313084	7606-9243	chaperone and heat shock protein (groEL)	546
HP_0011	2313085	9271-9624	co-chaperone (groES)	118
HP_0015	2313096	13702-13980	predicted coding region HP0015	93
HP_0016	2313097	13983-14243	predicted coding region HP0016	87
HP_0019	2313092	18380-19342	chemotaxis protein (cheV)	321
HP_0020	2313093	19342-20556	carboxynorspermidine decarboxylase (nspC)	405
HP_0031	2313114	31961-32371	predicted coding region HP0031	137
HP_0034	2313109	34895-35245	aspartate 1-decarboxylase (panD)	117
HP_0035	2313110	35251-35541	conserved hypothetical protein	97
HP_0039	2313126	38472-38741	predicted coding region HP0039	90
HP_0044	2313119	42105-43247	GDP-D-mannose dehydratase (rfbD)	381
HP_0045	2313120	43243-44172	nodulation protein (nolK)	310

HP_0049	2313129	48294-49283	predicted coding region HP0049	330
HP_0055	2313133	56227-57714	proline permease (putP)	496
HP_0068	2313140	72821-73417	urease accessory protein (ureG)	199
HP_0069	2313141	73449-74210	urease accessory protein (ureF)	254
HP_0072	2313153	75530-77236	urease beta subunit (urea amidohydrolase)(ureB)	569
HP_0073	2313154	77243-77956	urease alpha subunit (ureA) (ureaamidohydrolase)	238
HP_0074	2313155	78305-78775	lipoprotein signal peptidase (lspA)	157
HP_0075	2313156	78772-80106	urease protein (ureC)	445
HP_0076	2313157	80196-80462	ribosomal protein S20 (rps20)	89
HP_0083	2313164	88836-89222	ribosomal protein S9 (rps9)	129
HP_0084	2313165	89222-89644	ribosomal protein L13 (rpl13)	141
HP_0085	2313172	89960-90145	predicted coding region HP0085	62
HP_0089	2313168	95194-95886	pfs protein (pfs)	231
***** *HP_0090*	2313169	95900-96826	malonyl coenzyme A-acyl carrier proteintransacylase (fabD)	309
HP_0093	4580425	98939-99376	predicted coding region HP0093	146
HP_0095	2313182	99942-100469	predicted coding region HP0095	176
HP_0105	2313188	112831-113295	conserved hypothetical protein	155
HP_0107	2313190	114503-115420	cysteine synthetase (cysK)	306
HP_0109	2313191	116365-118224	chaperone and heat shock protein 70 (dnaK)	620
HP_0110	2313192	118257-118823	co-chaperone and heat shock protein (grpE)	189
HP_0112	2313200	119980-120672	predicted coding region HP0112	231
HP_0114	2313202	121005-122888	predicted coding region HP0114	628

HP_0115	2313197	122951-124492	flagellin B (flaB)	514
HP_0116	2313198	124658-126865	DNA topoisomerase I (topA)	736
+ *HP_0123*	2313207	135142-136977	threonyl-tRNA synthetase (thrS)	612
HP_0124	2313208	136977-137585	translation initiation factor IF-3 (infC)	203
HP_0125	2313210	137569-137760	ribosomal protein L35 (rpl35)	64
HP_0126	2313209	137857-138204	ribosomal protein L20 (rpl20)	116
HP_0127	2313212	138407-139264	outer membrane protein (omp4)	286
***** *HP_0133*	2313219	143597-144835	serine transporter (sdaC)	413
HP_0135	2313228	146444-146575	predicted coding region HP0135	44
HP_0136	2313221	146816-147271	bacterioferritin comigratory protein (bcp)	152
HP_0137	2313227	147284-147916	predicted coding region HP0137	211
HP_0139	2313223	149386-150111	conserved hypothetical secreted protein	242
HP_0145	2313232	157813-158508	cytochrome c oxidase, monoheme subunit,membrane-bound (fixO)	232
HP_0146	2313239	158519-158737	cbb3-type cytochrome c oxidase subunit Q (CcoQ)	73
HP_0147	2313233	158742-159599	cytochrome c oxidase, diheme subunit,membrane-bound (fixP)	286
HP_0148	2313246	159630-159833	predicted coding region HP0148	68
HP_0155	2313243	165256-165528	predicted coding region HP0155	91
HP_0156	2313244	165543-166142	predicted coding region HP0156	200
HP_0157	2313237	166150-166635	shikimic acid kinase I (aroK)	162
HP_0158	2313245	166660-167613	predicted coding region HP0158	318
HP_0162	2313249	170707-171426	conserved hypothetical protein	240
HP_0168	2313259	175146-175406	predicted coding region HP0168	87

HP_0176	2313265	182781-183701	fructose-bisphosphate aldolase (tsr)	307
HP_0177	2313266	183726-184286	translation elongation factor EF-P (efp)	187
HP_0182	2313270	188681-190183	lysyl-tRNA synthetase (lysS)	501
HP_0191	2313278	197129-197863	fumarate reductase, iron-sulfur subunit (frdB)	245
***** *HP_0192*	2313279	197859-200000	fumarate reductase, flavoprotein subunit (frdA)	714
HP_0193	2313280	200013-200777	fumarate reductase, cytochrome b subunit (frdC)	255
HP_0194	2313281	200992-201693	triosephosphate isomerase (tpi)	234
HP_0196	2313283	202543-203550	UDP-3-0-(3-hydroxymyristoyl) glucosamineN-acyltransferase (lpxD)	336
HP_0197	2313288	203618-204772	S-adenosylmethionine synthetase 2 (metX)	385
HP_0198	2313289	204842-205252	nucleoside diphosphate kinase (ndk)	137
HP_0199	2313294	205281-205634	predicted coding region HP0199	118
HP_0201	2313290	205874-206887	fatty acid/phospholipid synthesis protein(plsX)	338
HP_0202	2313291	206915-207907	beta-ketoacyl-acyl carrier protein synthase III(fabH)	331
HP_0215	2313305	223891-224688	CDP-diglyceride synthetase (cdsA)	266
HP_0220	2313311	228339-229499	synthesis of [Fe-S] cluster (nifS)	387
HP_0223	2313313	230973-232340	ATP-dependent protease (sms)	456
HP_0226	2313315	233932-234762	conserved hypothetical integral membraneprotein	277
HP_0230	2313323	240357-241085	CTP:CMP-3-deoxy-D-manno-octulosonate-cytidylyl-transferase (kdsB)	243
HP_0232	2313324	242006-242650	secreted protein involved in flagellar motility	215
HP_0234	2313326	243849-244376	conserved hypothetical integral	176

			membraneprotein	
HP_0241	2313335	251582-251974	predicted coding region HP0241	131
HP_0242	2313336	251970-252251	predicted coding region HP0242	94
HP_0243	2313332	252275-252706	neutrophil activating protein (napA)(bacterioferritin)	144
HP_0245	2313346	254057-254371	predicted coding region HP0245	105
HP_0248	2313341	257084-258169	conserved hypothetical protein	362
[+] *HP_0249*	2313347	258179-258715	predicted coding region HP0249	179
HP_0251	2313343	260364-261377	oligopeptide ABC transporter, permease protein(oppC)	338
[+] *HP_0255*	2313350	264709-265941	adenylosuccinate synthetase (purA)	411
HP_0256	2313360	265941-266366	predicted coding region HP0256	142
HP_0265	2313357	275250-275969	cytochrome c biogenesis protein (ccdA)	240
HP_0266	2313358	275982-277115	dihydroorotase (pyrC)	378
[+] *HP_0267*	2313359	277103-278329	chlorohydrolase	409
HP_0268	2313371	278393-278632	predicted coding region HP0268	80
HP_0270	2313372	280163-280648	predicted coding region HP0270	162
HP_0272	2313374	281617-282147	predicted coding region HP0272	177
HP_0274	2313365	282703-283098	conserved hypothetical protein	132
HP_0277	2313367	285032-285283	ferredoxin	84
HP_0280	2313370	287766-288749	heat shock protein B (ibpB)	328
HP_0282	2313386	290018-291457	predicted coding region HP0282	480
[+] *HP_0285*	2313381	294055-295308	conserved hypothetical protein	418
HP_0287	2313387	296953-297468	predicted coding region HP0287	172
HP_0291	2313389	307975-308262	predicted coding region HP0291	96

HP_0295	2313393	312148-314631	flagellin B homolog (fla)	828
HP_0296	2313394	314873-315184	ribosomal protein L21 (rpl21)	104
HP_0297	2313395	315202-315465	ribosomal protein L27 (rpl27)	88
HP_0299	2313397	317245-318246	dipeptide ABC transporter, permease protein(dppB)	334
HP_0300	2313398	318249-319103	dipeptide ABC transporter, permease protein(dppC)	285
HP_0302	2313400	319978-320781	dipeptide ABC transporter, ATP-binding protein(dppF)	268
HP_0306	2313404	323725-325014	glutamate-1-semialdehyde 2,1-aminomutase (hemL)	430
HP_0307	2313412	325014-325283	predicted coding region HP0307	90
HP_0308	2313413	325302-325703	predicted coding region HP0308	134
HP_0313	2313408	328950-330092	nitrite extrusion protein (narK)	381
HP_0318	2313418	334391-335143	conserved hypothetical protein	251
HP_0319	2313419	335207-336829	arginyl-tRNA synthetase (argS)	541
HP_0320	2313428	336835-337071	conserved hypothetical secreted protein	79
HP_0321	2313420	337146-337763	5'-guanylate kinase (gmk)	206
HP_0323	2313422	339415-339954	membrane bound endonuclease (nuc)	180
HP_0325	2313423	340832-341542	flagellar basal-body L-ring protein (flgH)	237
HP_0327	2313425	343113-343652	flagellar protein G (flaG)	180
HP_0330	2313431	345554-346543	ketol-acid reductoisomerase (ilvC)	330
HP_0332	2313433	347374-347604	cell division topological specificity factor(minE)	77
HP_0333	2313434	347607-348416	DNA processing chain A (dprA)	270
***** *HP_0349*	2313438	356453-358066	CTP synthetase (pyrG)	538

HP_0353	2313454	361777-362550	flagellar export protein (fliH)	258
HP_0354	2313455	362550-364403	deoxyxylulose-5-phosphate synthase, putative(dxs)	618
HP_0355	2313456	364403-366208	GTP-binding membrane protein (lepA)	602
HP_0363	2313465	372968-373594	L-isoaspartyl-protein carboxyl methyltransferase(pcm)	209
HP_0364	2313466	373607-374629	ribonucleoside diphosphate reductase, betasubunit (nrdB)	341
HP_0371	2313469	379643-380110	biotin carboxyl carrier protein (fabE)	156
HP_0372	2313476	380234-380803	deoxycytidine triphosphate deaminase (dcd)	190
HP_0375	2313484	383775-384203	predicted coding region HP0375	143
HP_0376	2313479	384259-385260	ferrochelatase (hemH)	334
HP_0378	2313481	386015-388822	cytochrome c biogenesis protein (ycf5)	936
HP_0380	2313483	390128-391471	glutamate dehydrogenase (gdhA)	448
$^{+}$ *HP_0381*	2313486	391540-392367	protoporphyrinogen oxidase (hemK)	276
HP_0385	2313501	395289-395516	predicted coding region HP0385	76
HP_0386	2313504	395532-395765	predicted coding region HP0386	78
$^{+}$ *HP_0388*	2313489	397649-398377	conserved hypothetical protein	243
HP_0389	2313490	398435-399073	superoxide dismutase (sodB)	213
HP_0390	2313491	399297-399794	adhesin-thiol peroxidase (tagD)	166
HP_0391	2313492	400055-400549	purine-binding chemotaxis protein (cheW)	165
***** *HP_0397*	2313497	407267-408838	phosphoglycerate dehydrogenase (serA)	524
HP_0399	2313498	409434-411101	ribosomal protein S1 (rps1)	556
HP_0403	2313509	415638-416621	phenylalanyl-tRNA synthetase, alpha subunit(pheS)	328

HP_0404	2313510	416702-417013	protein kinase C inhibitor (SP:P16436)	104
HP_0406	2313513	418383-418970	predicted coding region HP0406	196
HP_0419	2313522	433863-434645	conserved hypothetical protein	261
HP_0420	2313528	434665-435090	predicted coding region HP0420	142
HP_0421	2313523	435098-436264	type 1 capsular polysaccharide biosynthesisprotein J (capJ)	389
HP_0466	2313570	487913-488677	conserved hypothetical protein	255
HP_0467	2313571	488690-489034	conserved hypothetical integral membraneprotein	115
HP_0470	2313574	490978-492711	oligoendopeptidase F (pepF)	578
+ *HP_0480*	2313589	502628-504424	GTP-binding protein, fusA-homolog (yihK)	599
HP_0485	2313595	508125-509066	catalase-like protein	314
HP_0491	2313612	518288-518473	ribosomal protein L28 (rpL28)	62
*****+*HP_0500*	2313610	526549-527670	DNA polymerase III beta-subunit (dnaN)	374
HP_0514	2313629	542010-542459	ribosomal protein L9 (rpl9)	150
HP_0550	2313666	585391-586704	transcription termination factor Rho (rho)	438
HP_0551	2313667	586968-587168	ribosomal protein L31 (rpl31)	67
HP_0552	2313668	587196-588056	conserved hypothetical protein	287
HP_0553	2313669	588072-588752	conserved hypothetical protein	227
***** *HP_0557*	2313675	591533-592468	acetyl-coenzyme A carboxylase (accA)	312
***** *HP_0558*	2313676	592494-593729	beta ketoacyl-acyl carrier protein synthase II (fabF)	412
HP_0559	2313677	594040-594273	acyl carrier protein (acpP)	78
HP_0562	2313679	595292-595501	ribosomal protein S21 (rps21)	70
HP_0565	2313680	597296-597940	predicted coding region HP0565	215

HP_0571	2313691	603722-604297	conserved hypothetical integral membraneprotein	192
HP_0572	2313692	604315-604851	adenine phosphoribosyltransferase (apt)	179
HP_0573	2313701	604912-605241	predicted coding region HP0573	110
HP_0574	2313693	605296-605748	galactosidase acetyltransferase (lacA)	151
HP_0575	2313694	605772-606467	conserved hypothetical membrane protein	232
HP_0576	2313695	606479-607348	signal peptidase I (lepB)	290
HP_0577	2313696	607351-608226	methylene-tetrahydrofolate dehydrogenase (folD)	292
HP_0581	2313687	612000-613016	dihydroorotase (pyrC)	339
HP_0584	2313688	614859-615227	flagellar switch protein (fliN)	123
***** *HP_0585*	2313704	615312-615965	endonuclease III (nth)	218
HP_0588	2313706	620219-620557	ferrodoxin-like protein	113
HP_0590	2313708	621689-622507	ferredoxin oxidoreductase, beta subunit	273
HP_0591	2313709	622510-623067	ferredoxin oxidoreductase, gamma subunit	186
HP_0594	2313720	628139-628300	predicted coding region HP0594	54
HP_0596	2313719	630259-630834	predicted coding region HP0596	192
HP_0601	2313722	637282-638811	flagellin A (flaA)	510
HP_0602	2313723	638932-639585	endonuclease III	218
HP_0604	2313724	640268-641287	uroporphyrinogen decarboxylase (hemE)	340
HP_0616	2313738	661117-662055	chemotaxis protein (cheV)	313
[+] *HP_0618*	2313740	663843-664415	adenylate kinase (adk)	191
HP_0620	2313741	665747-666265	inorganic pyrophosphatase (ppa)	173
HP_0622	2313746	668662-669021	predicted coding region HP0622	120
***** *HP_0623*	2313743	669024-670370	UDP-N-acetylmuramate-alanine ligase (murC)	449

+ *HP_0624*	2313744	670366-671490	solute-binding signature and mitochondrialsignature protein (aspB)	375
HP_0625	2313753	671610-672686	protein E (gcpE)	359
HP_0626	2313754	672692-673894	tetrahydrodipicolinate N-succinyltransferase(dapD)	401
HP_0627	2313756	673909-674238	predicted coding region HP0627	110
HP_0628	2313757	674290-674964	predicted coding region HP0628	225
HP_0634	2313752	681542-682075	quinone-reactive Ni/Fe hydrogenase (hydD)	178
HP_0637	2313773	684149-684598	predicted coding region HP0637	150
HP_0639	2313760	685737-686414	conserved hypothetical protein	226
+ *HP_0640*	2313761	686477-687682	poly(A) polymerase (papS)	402
HP_0643	2313763	688746-690062	glutamyl-tRNA synthetase (gltX)	439
HP_0646	2313766	692043-692861	UDP-glucose pyrophosphorylase (galU)	273
HP_0648	2313767	693286-694551	UDP-N-acetylglucosamine enolpyruvyl transferase(murZ)	422
HP_0650	2313777	696063-696650	predicted coding region HP0650	196
HP_0654	2313779	699570-700649	conserved hypothetical protein	360
HP_0656	2313781	703397-704545	conserved hypothetical protein	383
*+ *HP_0661*	2313784	709533-709961	ribonuclease H (rnhA)	143
* *HP_0663*	2313786	710692-711786	chorismate synthase (aroC)	365
HP_0664	2313791	711827-712339	predicted coding region HP0664	171
HP_0680	2313806	729052-731415	ribonucleoside-diphosphate reductase 1 alphasubunit (nrdA)	788
HP_0688	2313822	739466-739963	predicted coding region HP0688	166
HP_0692	2313816	742440-743060	3-oxoadipate coA-transferase subunit B (yxjE)	207

HP_0697	2313821	750262-750765	predicted coding region HP0697	168
HP_0700	2313825	752106-752489	diacylglycerol kinase (dgkA)	128
HP_0702	2313831	754992-755465	predicted coding region HP0702	158
***** *HP_0714*	2313838	768092-769333	RNA polymerase sigma-54 factor (rpoN)	414
HP_0715	2313839	769339-770058	ABC transporter, ATP-binding protein	240
HP_0716	2313840	770074-770472	conserved hypothetical protein	133
HP_0720	2313853	773436-773594	predicted coding region HP0720	53
HP_0728	2313855	783154-784161	conserved hypothetical protein	336
HP_0735	2313857	790701-791159	xanthine guanine phosphoribosyl transferase(gpt)	153
HP_0736	2313858	791171-792277	phosphoserine aminotransferase (serC)	369
HP_0737	2313859	792424-792897	conserved hypothetical integral membraneprotein	158
HP_0739	2313861	794021-794743	2-hydroxy-6-oxohepta-2,4-dienoate hydrolase	241
HP_0741	2313863	796224-796706	conserved hypothetical protein	161
HP_0742	2313870	796774-797727	phosphoribosylpyrophosphate synthetase (prsA)	318
HP_0745	2313872	800330-801310	conserved hypothetical protein	327
HP_0748	2313874	803712-804380	cell division protein (ftsE)	223
HP_0751	2313876	806464-806820	polar flagellin (flaG)	119
HP_0754	2313888	809303-809539	predicted coding region HP0754	79
HP_0755	2313882	809635-810264	molybdopterin biosynthesis protein (moeB)	210
HP_0758	2313884	811424-812734	conserved hypothetical integral membraneprotein	437
HP_0759	2313885	812800-814050	conserved hypothetical integral	417

			membraneprotein	
HP_0760	2313886	814057-815442	conserved hypothetical protein	462
HP_0762	2313890	816317-816871	predicted coding region HP0762	185
HP_0763	2313887	816883-817761	cell division protein (ftsY)	293
***** *HP_0770*	2313898	822128-823201	flagellar biosynthetic protein (flhB)	358
HP_0771	2313903	823280-824014	predicted coding region HP0771	245
HP_0773	2313904	825365-826453	predicted coding region HP0773	363
HP_0774	2313900	826473-827678	tyrosyl-tRNA synthetase (tyrS)	402
HP_0776	2313905	830019-830270	predicted coding region HP0776	84
HP_0777	2313902	830285-831004	uridine 5'-monophosphate (UMP) kinase (pyrH)	240
HP_0778	2313912	831115-831795	predicted coding region HP0778	227
HP_0780	2313913	834559-834828	predicted coding region HP0780	90
HP_0784	2313917	838719-838850	predicted coding region HP0784	44
HP_0785	2313909	838881-839432	conserved hypothetical secreted protein	184
HP_0786	2313910	839580-842174	preprotein translocase subunit (secA)	865
HP_0794	2313923	851020-851607	ATP-dependent clp protease proteolytic component(clpP)	196
HP_0802	2313933	856624-857199	GTP cyclohydrolase II (ribA)	192
HP_0804	2313934	858219-859250	GTP cyclohydrolase II/3,4-dihydroxy-2-butanone4-phosphate synthase (ribA, ribB)	344
HP_0805	2313935	859424-860275	lipooligosaccharide 5G8 epitopebiosynthesis-associated protein (lex2B)	284
+ *HP_0808*	2313937	863554-863910	holo-acp synthase (acpS)	119
HP_0809	2313942	863920-864468	predicted coding region HP0809	183

HP_0813	2313945	866980-867594	conserved hypothetical protein	205
HP_0815	2313947	868381-869151	flagellar motor rotation protein (motA)	257
+ *HP_0816*	2313948	869157-869927	flagellar motor rotation protein (motB)	257
HP_0817	2313955	869927-870370	predicted coding region HP0817	148
HP_0821	2313951	873393-875174	excinuclease ABC subunit C (uvrC)	594
HP_0823	2313953	876454-876795	conserved hypothetical protein	114
***** *HP_0824*	2313958	876887-877204	thioredoxin (trxA)	106
HP_0825	2313959	877212-878144	thioredoxin reductase (trxB)	311
HP_0828	2313962	879966-880643	ATP synthase F0, subunit a (atpB)	226
HP_0830	2313964	882223-883581	Glu-tRNA(Gln) amidotransferase, subunit A(gatA)	453
HP_0831	2313965	883643-884230	conserved hypothetical ATP binding protein	196
***** *HP_0835*	2313970	887585-887866	histone-like DNA-binding protein HU (hup)	94
HP_0836	2313978	888471-888827	predicted coding region HP0836	119
HP_0837	2313979	888773-889078	predicted coding region HP0837	102
HP_0838	2313980	889101-889715	predicted coding region HP0838	205
HP_0840	2313972	891482-892480	flaA1 protein	333
HP_0842	2313981	893757-894491	predicted coding region HP0842	245
HP_0851	2313985	902875-903555	conserved hypothetical integral membraneprotein	227
HP_0860	2313992	913295-913813	conserved hypothetical protein	173
HP_0866	2313994	917926-918417	transcription elongation factor GreA (greA)	164
HP_0869	2313996	920008-920358	hydrogenase expression/formation protein (hypA)	117
HP_0875	2314010	925574-927088	catalase	505

HP_0877	2314012	929790-930260	Holliday junction endodeoxyribonuclease (ruvC)	157
HP_0878	2314018	930391-930561	predicted coding region HP0878	57
HP_0884	2314027	933398-935239	predicted coding region HP0884	614
HP_0886	2314022	936793-938187	cysteinyl-tRNA synthetase (cysS)	465
HP_0888	2314024	942350-943114	iron(III) dicitrate ABC transporter, ATP-bindingprotein (fecE)	255
HP_0889	2314025	943117-944094	iron(III) dicitrate ABC transporter, permeaseprotein (fecD)	326
HP_0899	2314034	951861-952091	hydrogenase expression/formation protein (hypC)	77
HP_0900	2314035	952094-952819	hydrogenase expression/formation protein (hypB)	242
HP_0908	2314044	959021-960835	flagellar hook (flgE)	605
HP_0918	2314058	973728-974156	predicted coding region HP0918	143
HP_0920	2314055	977520-978209	conserved hypothetical integral membraneprotein	230
HP_0925	2314062	988604-989182	recombinational DNA repair protein (recR)	193
HP_0928	2314065	991247-991786	GTP cyclohydrolase I (folE)	180
HP_0929	2314066	991805-992713	geranyltranstransferase (ispA)	303
HP_0930	2314067	992713-993513	stationary-phase survival protein (surE)	267
HP_0931	2314071	993513-993950	predicted coding region HP0931	146
HP_0932	2314072	993869-994168	predicted coding region HP0932	100
HP_0933	2314073	994173-994772	predicted coding region HP0933	200
HP_0939	2314077	999610-1000320	amino acid ABC transporter, permease	237

			protein(yckJ)	
HP_0944	2314082	1004932-1005306	conserved hypothetical protein	125
HP_0950	2314089	1010691-1011557	acetyl-CoA carboxylase beta subunit (accD)	289
HP_0951	2314100	1011635-1012249	predicted coding region HP0951	205
HP_0955	2314092	1014185-1015036	prolipoprotein diacylglyceryl transferase (lgt)	284
HP_0958	2314102	1016971-1017732	predicted coding region HP0958	254
+ *HP_0961*	2314097	1019388-1020323	glycerol-3-phosphate dehydrogenase, NAD(P)+dependent	312
HP_0975	2314117	1037309-1037587	Glu-tRNA(Gln) amidotransferase, subunit C(gatC)	93
HP_0980	2314122	1044552-1044851	conserved hypothetical secreted protein	100
HP_1010	2314153	1072429-1074453	polyphosphate kinase (ppk)	675
+ *HP_1013*	2314156	1076894-1077793	dihydrodipicolinate synthetase (dapA)	300
HP_1016	2314161	1079133-1079732	phosphatidylglycerophosphate synthase (pgsA)	200
HP_1019	2314163	1081537-1082865	serine protease (htrA)	443

HP_1025	2314167	1088509-<br>1088877	putative heat shock protein (hspR)	123
HP_1026	2314168	1088877-<br>1090049	conserved hypothetical helicase-like protein	391
HP_1027	2314174	1090212-<br>1090661	ferric uptake regulation protein (fur)	150
HP_1031	2314176	1092613-<br>1093674	flagellar motor switch protein (fliM)	354
HP_1038	2314182	1098565-<br>1099065	3-dehydroquinase type II (aroQ)	167
HP_1040	2314183	1100517-<br>1100786	ribosomal protein S15 (rps15)	90
HP_1041	2314189	1100927-<br>1103125	flagellar biosynthesis protein (flhA)	733
HP_1043	2314190	1104748-<br>1105416	response regulator	223
HP_1046	2314193	1109182-<br>1109619	conserved hypothetical protein	146
HP_1047	2314194	1109615-<br>1109947	ribosome-binding factor A (rbfA)	111
HP_1049	2314196	1112781-<br>1113047	conserved hypothetical protein	89
HP_1050	2314197	1113022-<br>1113900	homoserine kinase (thrB)	293
HP_1052	2314201	1114452-	UDP-3-0-acyl N-acetylglcosamine	295

		1115336	deacetylase(envA)	
HP_1058	2314202	1119911-1120720	3-methyl-2-oxobutanoate hydroxymethyltransferase(panB)	270
HP_1059	2314203	1120723-1121730	Holliday junction DNA helicase (ruvB)	336
HP_1063	2314206	1124072-1124605	glucose-inhibited division protein (gidB)	178
HP_1065	2314215	1124835-1125239	predicted coding region HP1065	135
HP_1066	2314207	1125378-1125977	conserved hypothetical protein	200
HP_1067	2314217	1126268-1126639	chemotaxis protein (cheY)	124
HP_1071	2314220	1129798-1130508	phosphatidylserine synthase (pssA)	237
***** *HP_1073*	2314224	1132746-1132943	copper ion binding protein (copP)	66
HP_1081	2314238	1139472-1140092	predicted coding region HP1081	207
HP_1082	2314232	1140089-1141741	multidrug resistance protein (msbA)	551
HP_1085	2314240	1144517-1145029	predicted coding region HP1085	171
HP_1088	2314236	1146594-1148516	transketolase A (tktA)	641

HP_1101	2314250	1162198-1163472	glucose-6-phosphate dehydrogenase (g6pD)	425
HP_1102	2314251	1163486-1164166	glucose-6-phosphate 1-dehydrogenase (devB)	227
+ *HP_1103*	2314252	1164156-1165163	glucokinase (glk)	336
HP_1108	2314259	1170138-1170695	pyruvate ferredoxin oxidoreductase, gammasubunit	186
HP_1109	2314260	1170714-1171103	pyruvate ferredoxin oxidoreductase, deltasubunit	130
HP_1111	2314262	1172352-1173293	pyruvate ferredoxin oxidoreductase, betasubunit	314
HP_1120	2314274	1186445-1186876	predicted coding region HP1120	144
HP_1129	2314280	1192860-1193258	biopolymer transport protein (exbD)	133
HP_1131	2314282	1193894-1194265	ATP synthase F1, subunit epsilon (atpC)	124
HP_1132	2314283	1194276-1195682	ATP synthase F1, subunit beta (atpD)	469
HP_1133	2314284	1195708-1196610	ATP synthase F1, subunit gamma (atpG)	301
* *HP_1134*	2314285	1196628-1198136	ATP synthase F1, subunit alpha (atpA)	503
HP_1135	2314286	1198160-	ATP synthase F1, subunit delta (atpH)	180

		1198699		
* *HP_1136*	2314287	1198703-1199215	ATP synthase F0, subunit b (atpF)	171
HP_1137	2314288	1199222-1199653	ATP synthase F0, subunit b' (atpF')	144
HP_1138	2314294	1199767-1200636	plasmid replication-partition related protein	290
* *HP_1147*	2314302	1211234-1211587	ribosomal protein L19 (rpl19)	118
HP_1151	2314310	1213221-1213448	ribosomal protein S16 (rpS16)	76
* *HP_1152*	2314305	1213525-1214868	signal recognition particle protein (ffh)	448
HP_1159	2314309	1225846-1226376	cell filamentation protein (fic)	177
HP_1160	2314318	1226473-1226892	conserved hypothetical protein	140
HP_1161	2314319	1226950-1227441	flavodoxin (fldA)	164
HP_1163	2314326	1228239-1228427	predicted coding region HP1163	63
+ *HP_1164*	2314321	1228455-1229426	thioredoxin reductase (trxB)	324
* *HP_1166*	2314323	1230660-1232294	glucose-6-phosphate isomerase (pgi)	545

HP_1170	2314329	1237227-1237895	glutamine ABC transporter, permease protein(glnP)	223
+ *HP_1171*	2314330	1237900-1238643	glutamine ABC transporter, ATP-binding protein(glnQ)	248
HP_1173	2314338	1239650-1240198	predicted coding region HP1173	183
HP_1175	2314333	1241828-1243132	conserved hypothetical integral membraneprotein	435
* *HP_1178*	2314335	1245967-1246665	purine-nucleoside phosphorylase (deoD)	233
HP_1186	2314346	1255772-1256377	carbonic anhydrase	202
HP_1189	2314350	1259622-1260659	aspartate-semialdehyde dehydrogenase (asd)	346
HP_1190	2314351	1260649-1261974	histidyl-tRNA synthetase (hisS)	442
HP_1196	2314355	1267401-1267865	ribosomal protein S7 (rps7)	155
HP_1199	2314361	1277276-1277650	ribosomal protein L7/L12 (rpl7/l12)	125
HP_1201	2314363	1278302-1279003	ribosomal protein L1 (rpl1)	234
HP_1202	2314364	1279051-1279473	ribosomal protein L11 (rpl11)	141
HP_1203	2314365	1279494-	transcription termination factor NusG (nusG)	176

		1280021		
HP_1205	2314366	1280682-1281878	translation elongation factor EF-Tu (tufB)	399
HP_1207	2314372	1284327-1284992	predicted coding region HP1207	222
HP_1211	2314384	1287786-1288028	predicted coding region HP1211	81
HP_1212	2314374	1288091-1288405	ATP synthase F0, subunit c (atpE)	105
HP_1220	2314379	1295707-1296390	ABC transporter, ATP-binding protein (yhcG)	228
HP_1223	2314397	1300041-1300370	predicted coding region HP1223	110
HP_1225	2314388	1301104-1301493	conserved hypothetical integral membraneprotein	130
HP_1228	2314391	1303120-1303584	invasion protein (invA)	155
HP_1229	2314392	1303589-1304803	aspartokinase (lysC)	405
HP_1231	2314393	1305342-1305995	DNA polymerase III delta prime subunit (holB)	218
HP_1233	2314399	1307235-1307693	predicted coding region HP1233	153
HP_1234	2314395	1308086-1308979	conserved hypothetical integral membraneprotein	298

HP_1236	2314406	1310312-1310860	predicted coding region HP1236	183
HP_1240	2314403	1313617-1314186	conserved hypothetical protein	190
HP_1242	2314405	1316851-1317078	conserved hypothetical protein	76
HP_1254	2314418	1329963-1330682	biotin synthesis protein (bioC)	240
HP_1256	2314423	1331404-1331958	ribosome releasing factor (frr)	185
HP_1257	2314424	1331965-1332567	orotate phosphoribosyltransferase (pyrE)	201
HP_1258	2314425	1332560-1333021	conserved hypothetical mitochondrial protein 4	154
HP_1259	2314426	1333094-1333708	conserved hypothetical protein	205
HP_1260	2314427	1333813-1334211	NADH-ubiquinone oxidoreductase, NQO7 subunit(NQO7)	133
HP_1261	2314428	1334214-1334690	NADH-ubiquinone oxidoreductase, NQO6 subunit(NQO6)	159
HP_1262	2314429	1334690-1335487	NADH-ubiquinone oxidoreductase, NQO5 subunit(NQO5)	266
HP_1263	2314430	1335492-1336718	NADH-ubiquinone oxidoreductase, NQO4 subunit(NQO4)	409
HP_1264	2314442	1336718-	predicted coding region HP1264	76

		1336945		
HP_1269	2314434	1342120-1342665	NADH-ubiquinone oxidoreductase, NQO10 subunit(NQO10)	182
HP_1273	2314438	1346337-1347806	NADH-ubiquinone oxidoreductase, NQO14 subunit(NQO14)	490
HP_1281	2314447	1356689-1357270	anthranilate synthase component II (trpD)	194
HP_1285	2314453	1361660-1362349	conserved hypothetical secreted protein	230
HP_1290	2314456	1365401-1366060	nicotinamide mononucleotide transporter (pnuC)	220
HP_1291	2314457	1366051-1366662	conserved hypothetical protein	204
HP_1292	2314458	1367375-1367722	ribosomal protein L17 (rpl17)	116
HP_1293	2314459	1367725-1368756	DNA-directed RNA polymerase, alpha subunit(rpoA)	344
HP_1294	2314460	1368771-1369394	ribosomal protein S4 (rps4)	208
HP_1295	2314461	1369407-1369799	ribosomal protein S11 (rps11)	131
HP_1296	2314462	1369825-1370184	ribosomal protein S13 (rps13)	120
HP_1299	2314463	1370601-1371359	methionine amino peptidase (map)	253

HP_1300	2314464	1371362-<br>1372621	preprotein translocase subunit (secY)	420
HP_1301	2314465	1372667-<br>1373071	ribosomal protein L15 (rpl15)	135
HP_1302	2314466	1373088-<br>1373546	ribosomal protein S5 (rps5)	153
HP_1303	2314467	1373546-<br>1373902	ribosomal protein L18 (rpl18)	119
HP_1305	2314469	1374463-<br>1374855	ribosomal protein S8 (rps8)	131
HP_1307	2314470	1375063-<br>1375605	ribosomal protein L5 (rpl5)	181
HP_1311	2314485	1376486-<br>1376683	ribosomal protein L29 (rpL29)	66
HP_1313	2314475	1377101-<br>1377802	ribosomal protein S3 (rps3)	234
HP_1314	2314476	1377809-<br>1378174	ribosomal protein L22 (rpl22)	122
HP_1316	2314478	1378479-<br>1379306	ribosomal protein L2 (rpl2)	276
HP_1318	2314480	1379611-<br>1380255	ribosomal protein L4 (rpl4)	215
HP_1319	2314481	1380293-<br>1380865	ribosomal protein L3 (rpl3)	191
HP_1323	2314491	1383302-	ribonuclease HII (rnhB)	209

		1383928		
HP_1325	2314492	1384220-1385608	fumarase (fumC)	463
HP_1326	2314500	1385783-1386157	predicted coding region HP1326	125
HP_1330	2314495	1391530-1391874	conserved hypothetical integral membraneprotein	115
HP_1336	2314515	1397061-1397819	predicted coding region HP1336	253
+ *HP_1338*	2314505	1398330-1398773	conserved hypothetical protein	148
HP_1346	2314512	1406102-1407091	glyceraldehyde-3-phosphate dehydrogenase (gap)	330
HP_1356	2314524	1417892-1418899	quinolinate synthetase A (nadA)	336
HP_1357	2314525	1418892-1419692	phosphatidylserine decarboxylase proenzyme(psd)	267
HP_1358	2314534	1419689-1420192	predicted coding region HP1358	168
HP_1360	2314526	1420695-1421576	4-hydroxybenzoate octaprenyltransferase (ubiA)	294
HP_1372	2314542	1436254-1436997	rod shape-determining protein (mreC)	248
HP_1373	2314543	1437004-1438044	rod shape-determining protein (mreB)	347

HP_1374	2314544	1438090-1439427	ATP-dependent protease ATPase subunit (clpX)	446
HP_1377	2314555	1440899-1441336	predicted coding region HP1377	146
HP_1378	2314548	1441694-1442353	competence lipoprotein (comL)	220
HP_1386	2314553	1448099-1448749	D-ribulose-5-phosphate 3 epimerase (rpe)	217
HP_1391	2314568	1451760-1452053	predicted coding region HP1391	98
+ *HP_1398*	2314564	1458585-1459724	alanine dehydrogenase (ald)	380
HP_1407	2314579	1471967-1472842	conserved hypothetical integral membraneprotein	292
HP_1413	2314588	1484032-1484475	conserved hypothetical protein	148
HP_1419	2314593	1489411-1489674	flagellar biosynthetic protein (fliQ)	88
***** *HP_1420*	2314594	1489688-1490989	flagellar export protein ATP synthase (fliI)	434
HP_1421	2314595	1490993-1491904	conjugative transfer regulon protein (trbB)	304
HP_1428	2314600	1497370-1498440	conserved hypothetical protein	357
***** *HP_1429*	2314601	1498440-	polysialic acid capsule expression protein	329

		1499426	(kpsF)	
[+] *HP_1431*	2314603	1501520-1502332	16S rRNA (adenosine-N6,N6-)-dimethyltransferase(ksgA)	271
HP_1440	2314621	1513146-1513922	predicted coding region HP1440	259
HP_1442	2314614	1514554-1514781	carbon storage regulator (csrA)	76
HP_1444	2314616	1515584-1516039	small protein (smpB)	152
HP_1449	2314625	1517547-1517897	conserved hypothetical protein	117
HP_1458	2314636	1527775-1528086	thioredoxin	104
HP_1459	2314637	1528144-1528929	conserved hypothetical protein	262
HP_1468	2314646	1538765-1539784	branched-chain-amino-acid aminotransferase(ilvE)	340
HP_1474	2314650	1547338-1547910	thymidylate kinase (tmk)	191
HP_1476	2314652	1548388-1548948	phenylacrylic acid decarboxylase	187
HP_1485	2314657	1557746-1558315	conserved hypothetical protein	190
HP_1492	2314672	1566261-1566527	conserved hypothetical nifU-like protein	89

***** *HP_1495*	2314674	1568504-1569451	transaldolase (tal)	316
HP_1496	2314675	1569509-1570042	general stress protein (ctc)	178
HP_1497	2314676	1570055-1570612	peptidyl-tRNA hydrolase (pth)	186
HP_1504	2314678	1577242-1577955	conserved hypothetical protein	238
HP_1509	2314690	1582845-1583630	conserved hypothetical integral membraneprotein	262
HP_1511	2314696	1583967-1584290	predicted coding region HP1511	108
***** *HP_1514*	2314694	1588569-1589753	transcription termination factor NusA (nusA)	395
***** *HP_1523*	2314702	1602091-1603959	DNA recombinase (recG)	623
HP_1526	2314703	1605027-1605776	exodeoxyribonuclease (lexA)	250
HP_1530	2314710	1609150-1609689	purine nucleoside phosphorylase (punB)	180
HP_1531	2314716	1609735-1609971	predicted coding region HP1531	79
HP_1533	2314712	1611722-1612414	conserved hypothetical protein	231
HP_1540	2314722	1617984-	ubiquinol cytochrome c oxidoreductase,	167

		1618484	Rieske2Fe-2S subunit (fbcF)	
HP_1542	2314731	1621615-1622022	predicted coding region HP1542	136
HP_1543	2314724	1621944-1622879	toxR-activated gene (tagE)	312
HP_1547	2314727	1625500-1627917	leucyl-tRNA synthetase (leuS)	806
HP_1548	2314728	1627930-1628265	conserved hypothetical integral membraneprotein	112
HP_1549	2314729	1628278-1629246	protein-export membrane protein (secF)	323
HP_1554	2314737	1635686-1636477	ribosomal protein S2 (rps2)	264
HP_1555	2314738	1636480-1637544	translation elongation factor EF-Ts (tsf)	355
HP_1557	2314740	1640002-1640328	flagellar basal-body protein (fliE)	109
HP_1558	2314741	1640459-1640941	flagellar basal-body rod protein (flgC)	161
HP_1559	2314742	1640957-1641376	flagellar basal-body rod protein (flgB)	140
HP_1568	2314755	1650097-1650645	predicted coding region HP1568	183
HP_1569	2314756	1650648-1651238	predicted coding region HP1569	197

HP_1570	2314751	1651216-1651707	conserved hypothetical protein	164
HP_1573	2314758	1653860-1654621	conserved hypothetical protein	254
HP_1574	2314759	1654696-1655313	riboflavin synthase alpha subunit (ribC)	206
HP_1575	2314760	1655317-1655586	ABC transporter, putative	90
HP_1576	2314761	1655611-1656591	ABC transporter, ATP-binding protein (abc)	327
HP_1577	2314762	1656596-1657240	ABC transporter, permease protein (yaeE)	215
HP_1583	2314766	1661400-1662320	pyridoxal phosphate biosynthetic protein A(pdxA)	307
HP_1585	2314768	1663590-1664375	flagellar basal-body rod protein (flgG)	262
