# Supplementary material for: Recombination-independent rapid convergent evolution of the gastric pathogen Helicobacter pylori
Source: BMC Genomics. 2018 Nov 21;19:835. doi: 10.1186/s12864-018-5231-7 (PMC6249973; doi:10.1186/s12864-018-5231-7)
Supplement: Supplementary file 2 — Figure S1. Frequency of recombinant regions as detected by PhiPack using single (intra-genic) vs. multiple (inter-genic) genes in ‘global’ subset and ‘local’ (Amerindian) subset of strains (as denoted in Fig. 1). Figure S2. The synDss statistic landscapes for 50 randomly selected core genes of H. pylori: (A) 25 recombinant and (B) 25 non-recombinant, as designated by three recombination detection statistics in PhiPack software. Figure S3. Average frequency distribution of mutations in encoded proteins of randomly selected subsets of H. pylori non-recombinant core genes with convergent mutations. Figure S4. Average frequency distribution of mutations of encoded proteins in simulated datasets of 25 H. pylori genes (one of the randomly selected replicates from Figure S3) using two different mutation-rate constraints. (PDF 2545 kb) [file 12864_2018_5231_MOESM2_ESM.pdf]

Figure S1. Frequency of recombinant regions as detected by PhiPack using single (intra-genic) vs. multiple (inter-genic) genes in 'global' subset and 'local' (Amerindian) subset of strains (as denoted in Fig. 1).

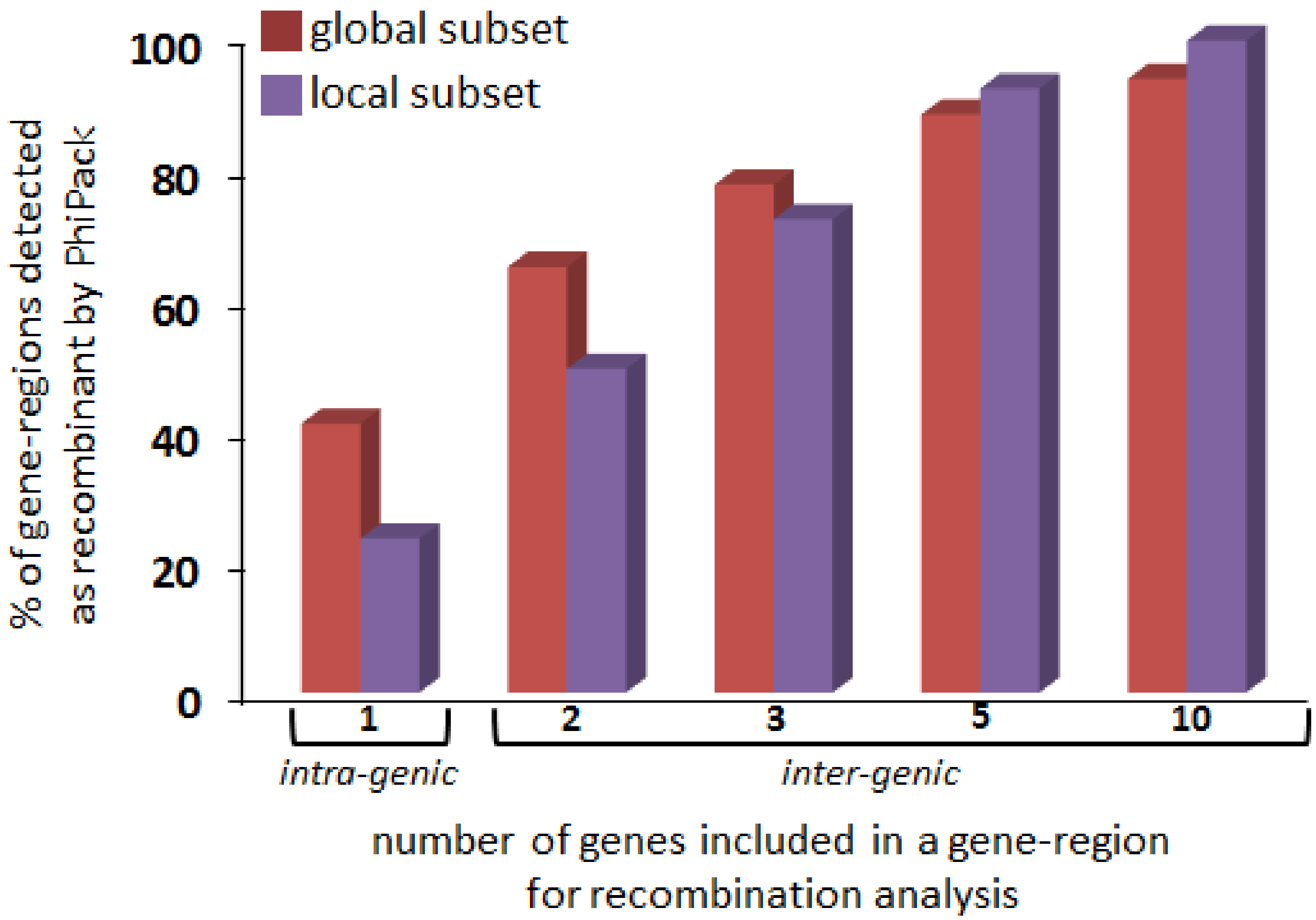

Figure S2. The synDss statistic landscapes for 50 randomly selected core genes of *H. pylori*: (A) 25 recombinant and (B) 25 non-recombinant, as designated by three recombination detection statistics in PhiPack software.

(A) 25 randomly selected core genes designated as **recombinants** by PhiPack

HP_0029

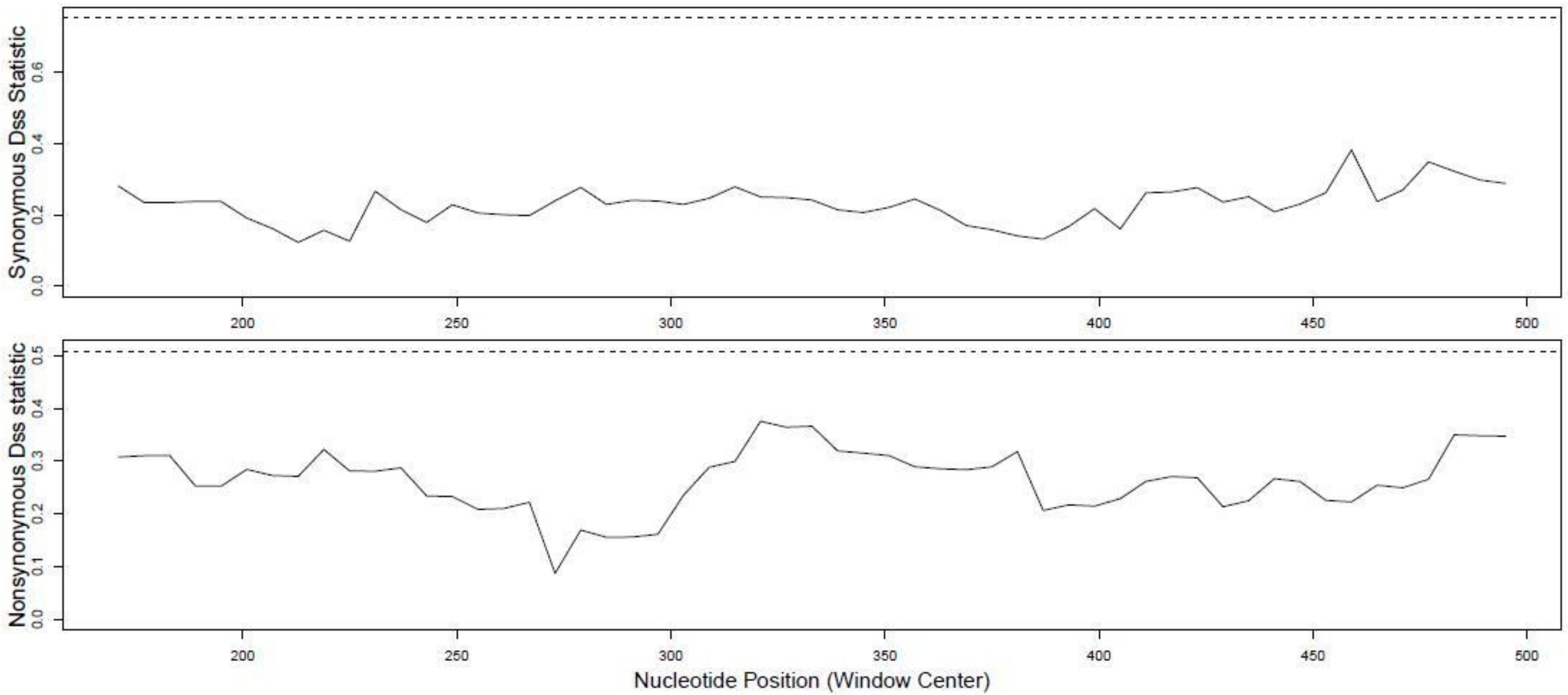

Dss statistic landscapes for synonymous and nonsynonymous changes. Dotted horizontal lines represent the 95% significance level for each test, from a parametric bootstrap with 500 replicates.

(A) 25 randomly selected core genes designated as **recombinants** by PhiPack (CONTD.)

HP_0042

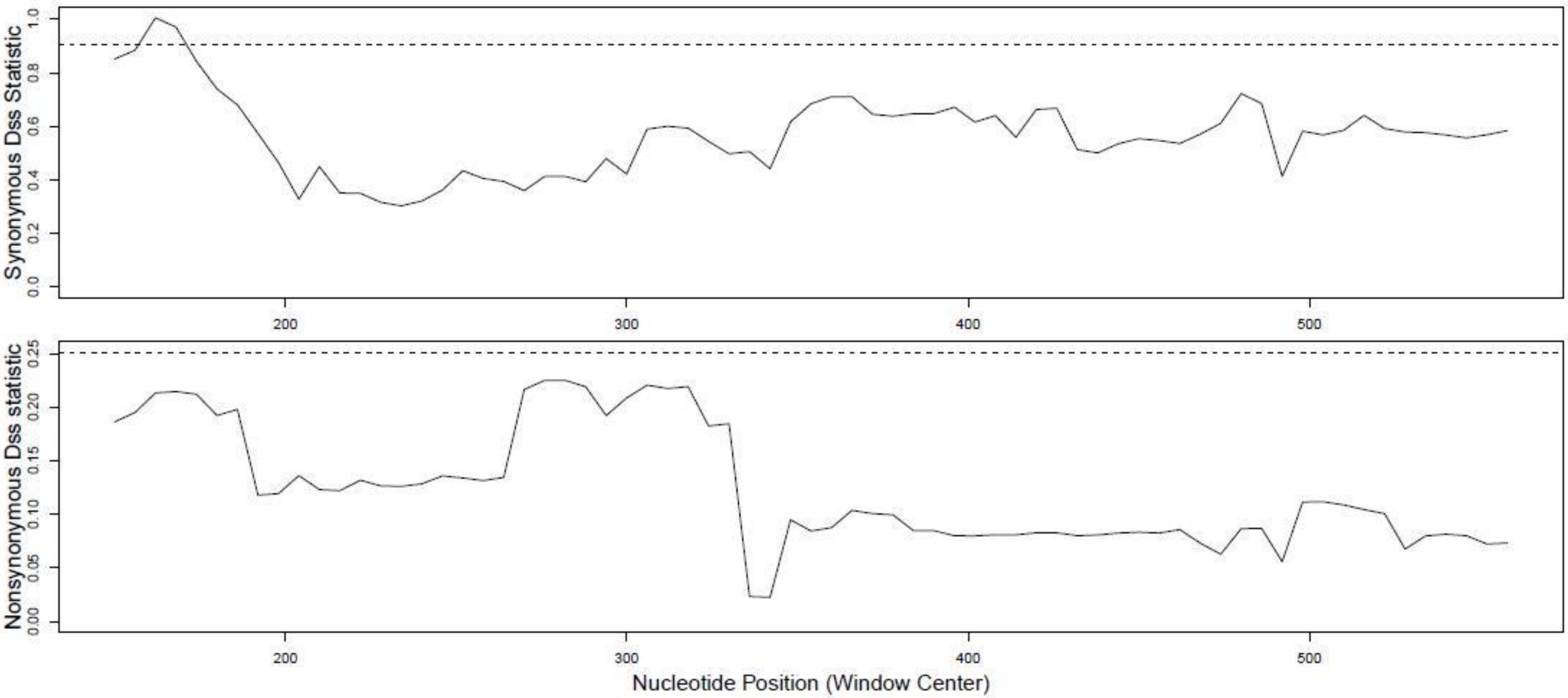

Dss statistic landscapes for synonymous and nonsynonymous changes. Dotted horizontal lines represent the 95% significance level for each test, from a parametric bootstrap with 500 replicates.

(A) 25 randomly selected core genes designated as **recombinants** by PhiPack (CONTD.)

HP_0102

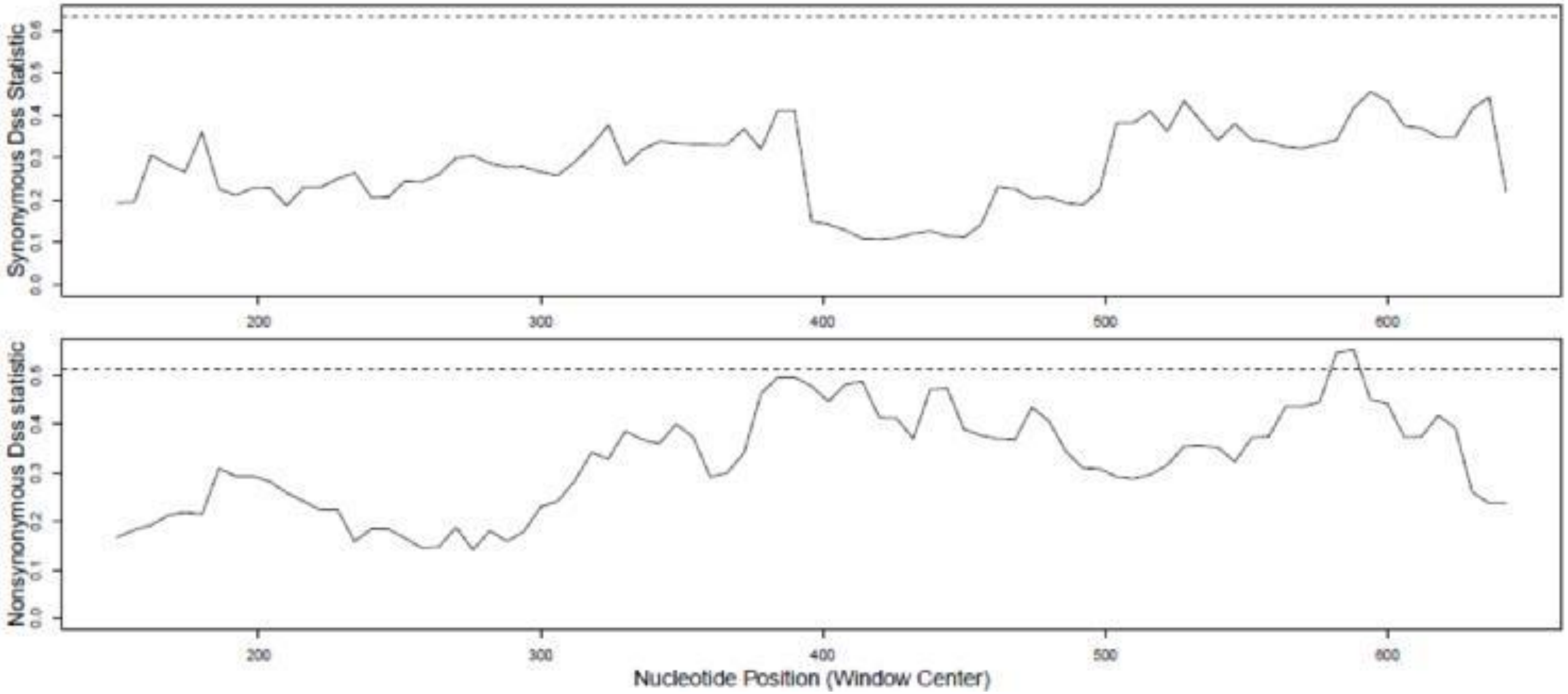

Dss statistic landscapes for synonymous and nonsynonymous changes. Dotted horizontal lines represent the 95% significance level for each test, from a parametric bootstrap with 500 replicates.

(A) 25 randomly selected core genes designated as **recombinants** by PhiPack (CONTD.)

HP_0150

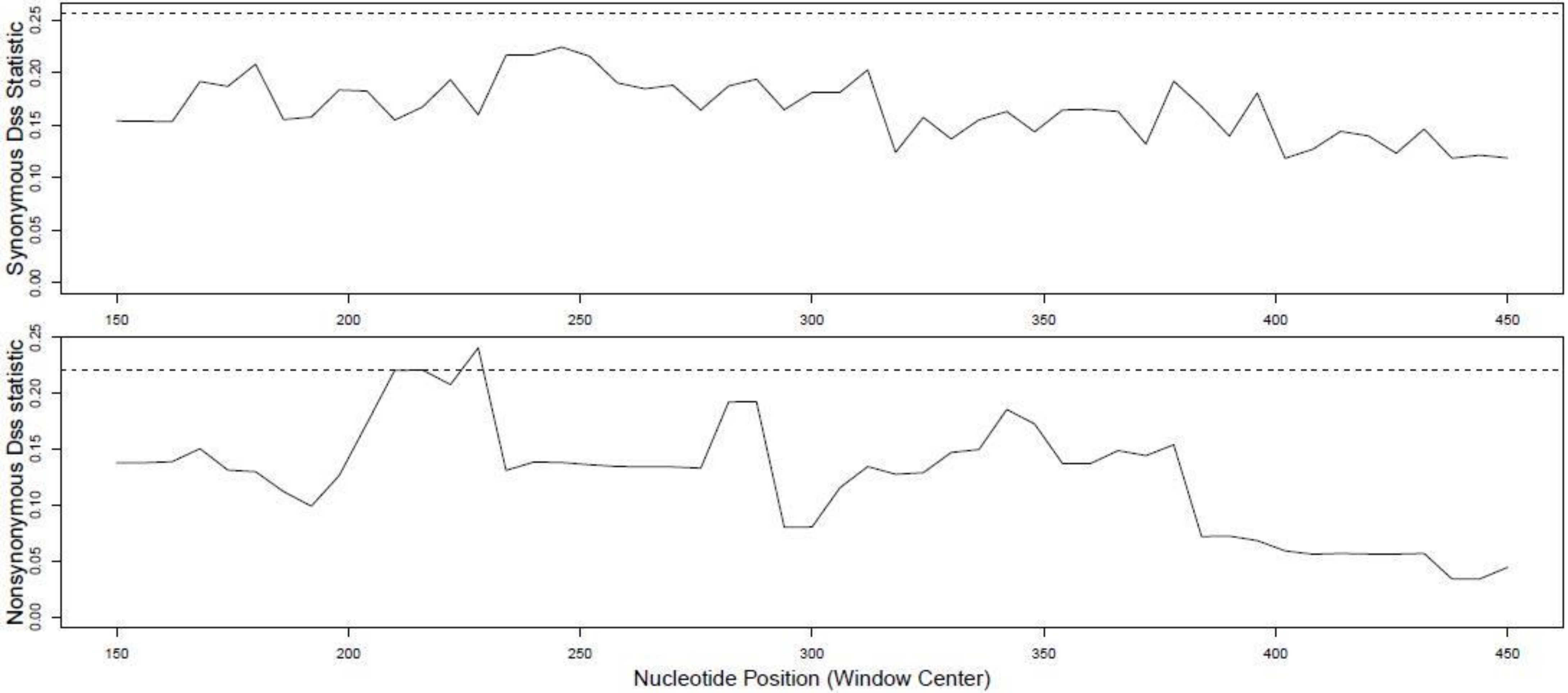

Dss statistic landscapes for synonymous and nonsynonymous changes. Dotted horizontal lines represent the 95% significance level for each test, from a parametric bootstrap with 500 replicates.

(A) 25 randomly selected core genes designated as **recombinants** by PhiPack (CONTD.)

HP_0214

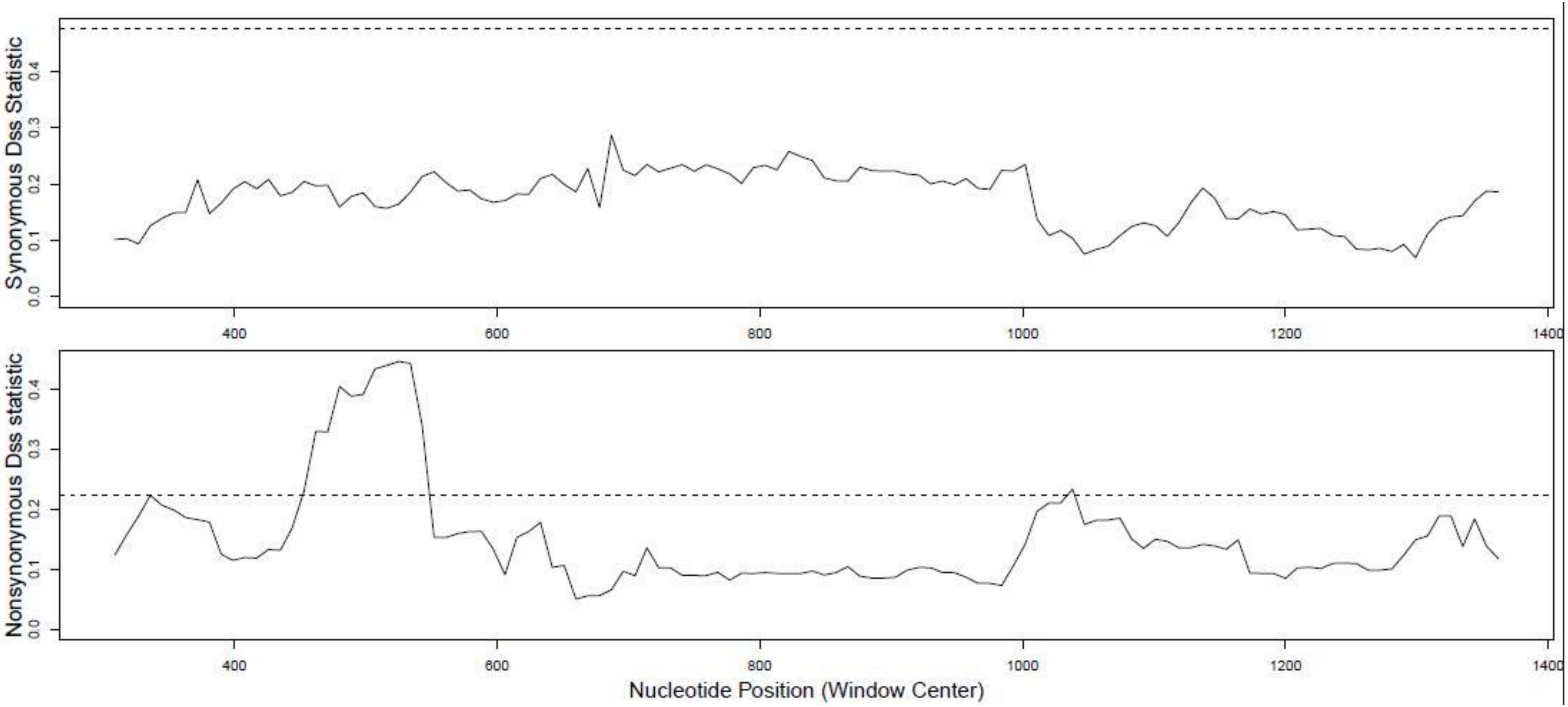

Dss statistic landscapes for synonymous and nonsynonymous changes. Dotted horizontal lines represent the 95% significance level for each test, from a parametric bootstrap with 500 replicates.

(A) 25 randomly selected core genes designated as **recombinants** by PhiPack (CONTD.)

HP_0298

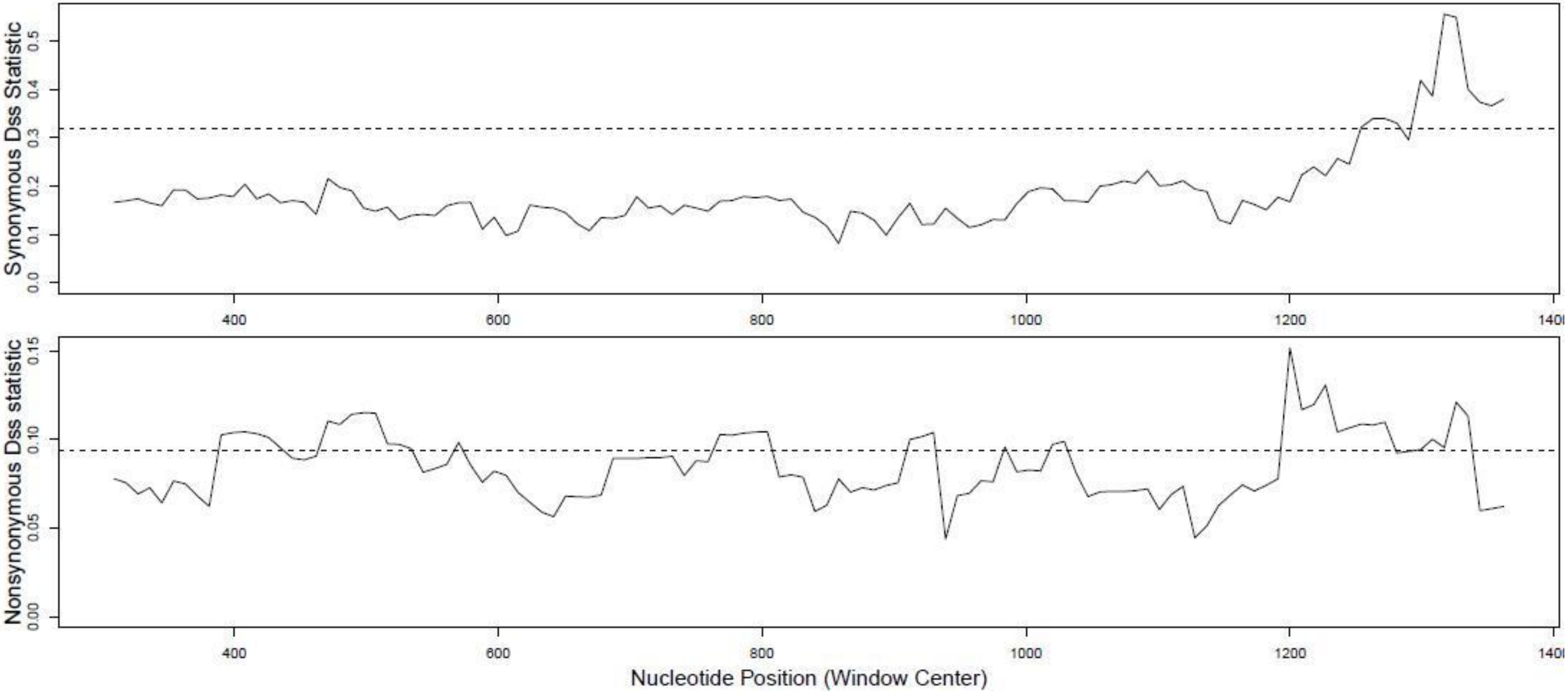

Dss statistic landscapes for synonymous and nonsynonymous changes. Dotted horizontal lines represent the 95% significance level for each test, from a parametric bootstrap with 500 replicates.

(A) 25 randomly selected core genes designated as **recombinants** by PhiPack (CONTD.)

HP_0352

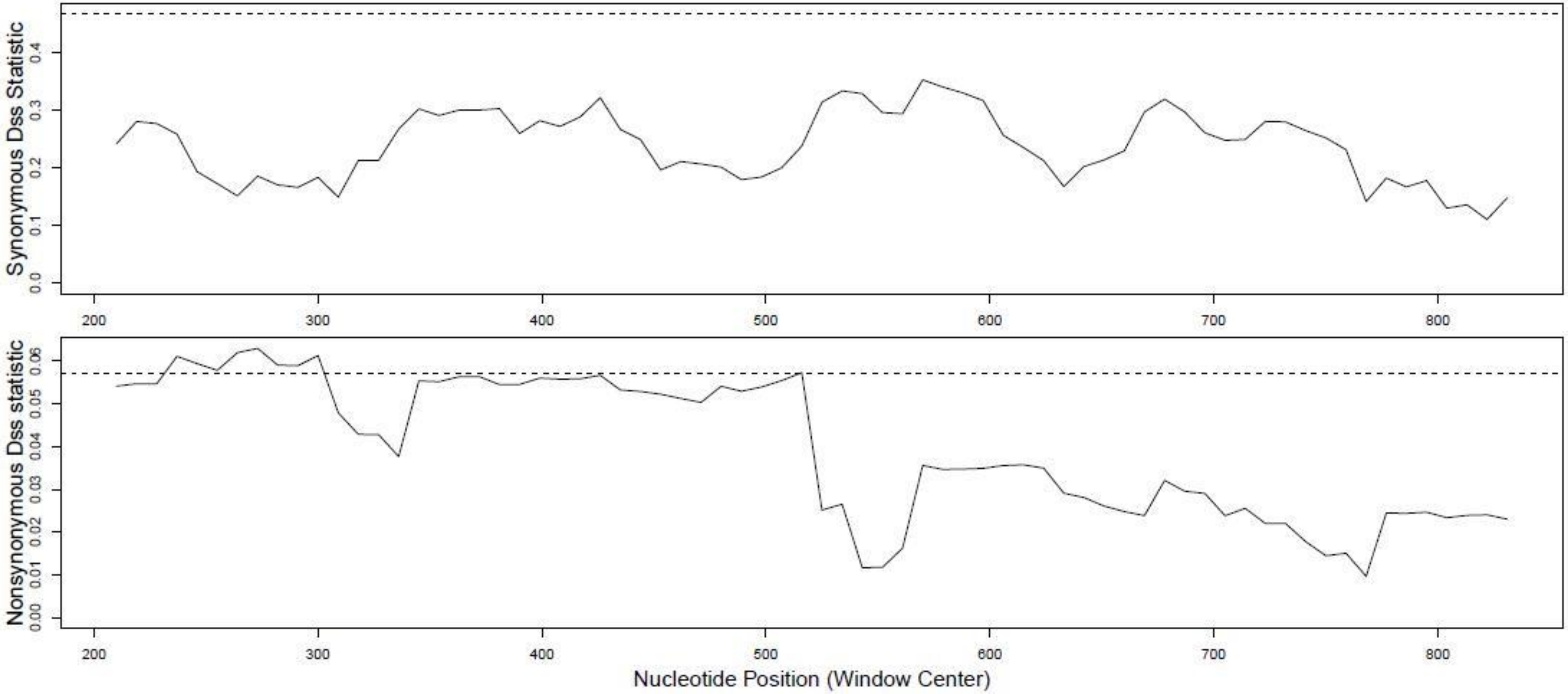

Dss statistic landscapes for synonymous and nonsynonymous changes. Dotted horizontal lines represent the 95% significance level for each test, from a parametric bootstrap with 500 replicates.

(A) 25 randomly selected core genes designated as **recombinants** by PhiPack (CONTD.)

HP_0393

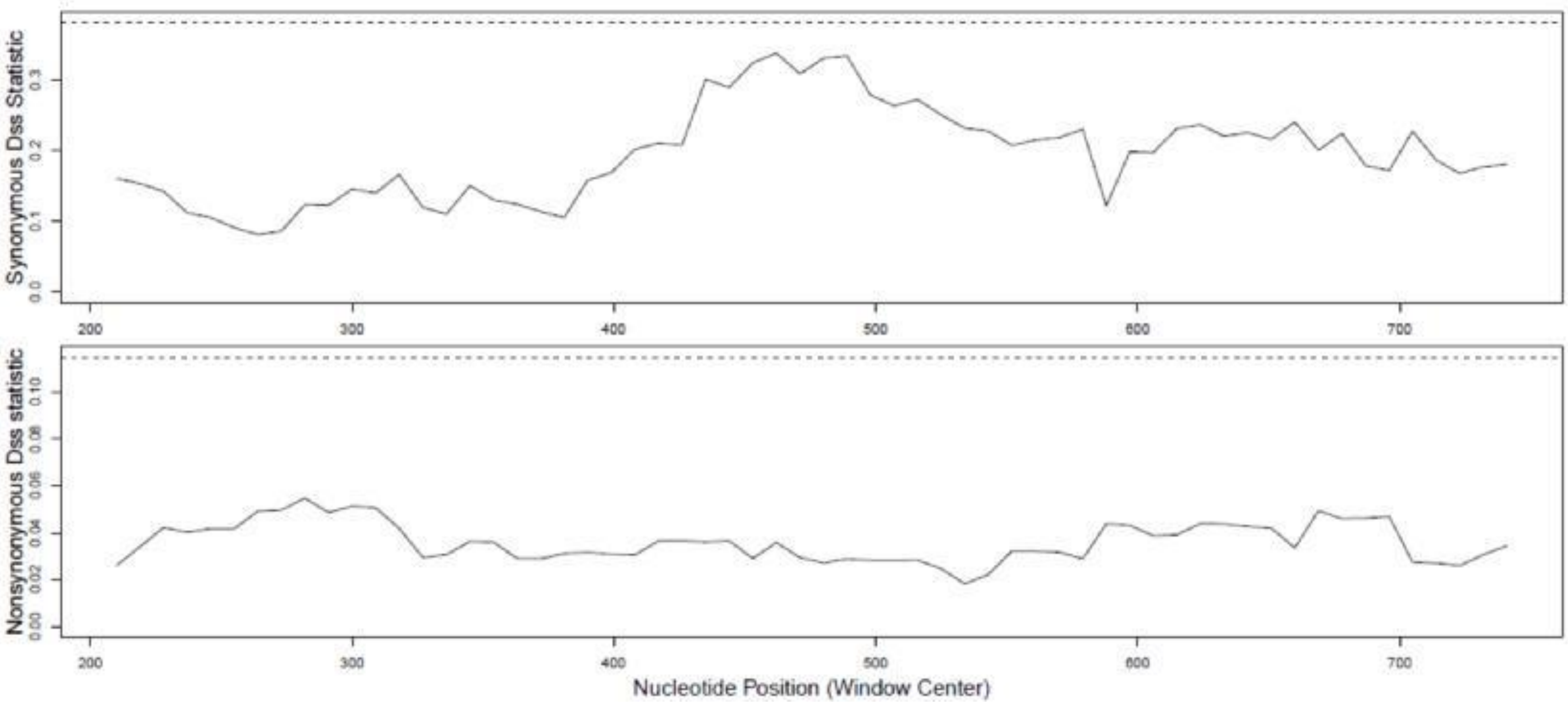

Dss statistic landscapes for synonymous and nonsynonymous changes. Dotted horizontal lines represent the 95% significance level for each test, from a parametric bootstrap with 500 replicates.

(A) 25 randomly selected core genes designated as **recombinants** by PhiPack (CONTD.)

HP_0493

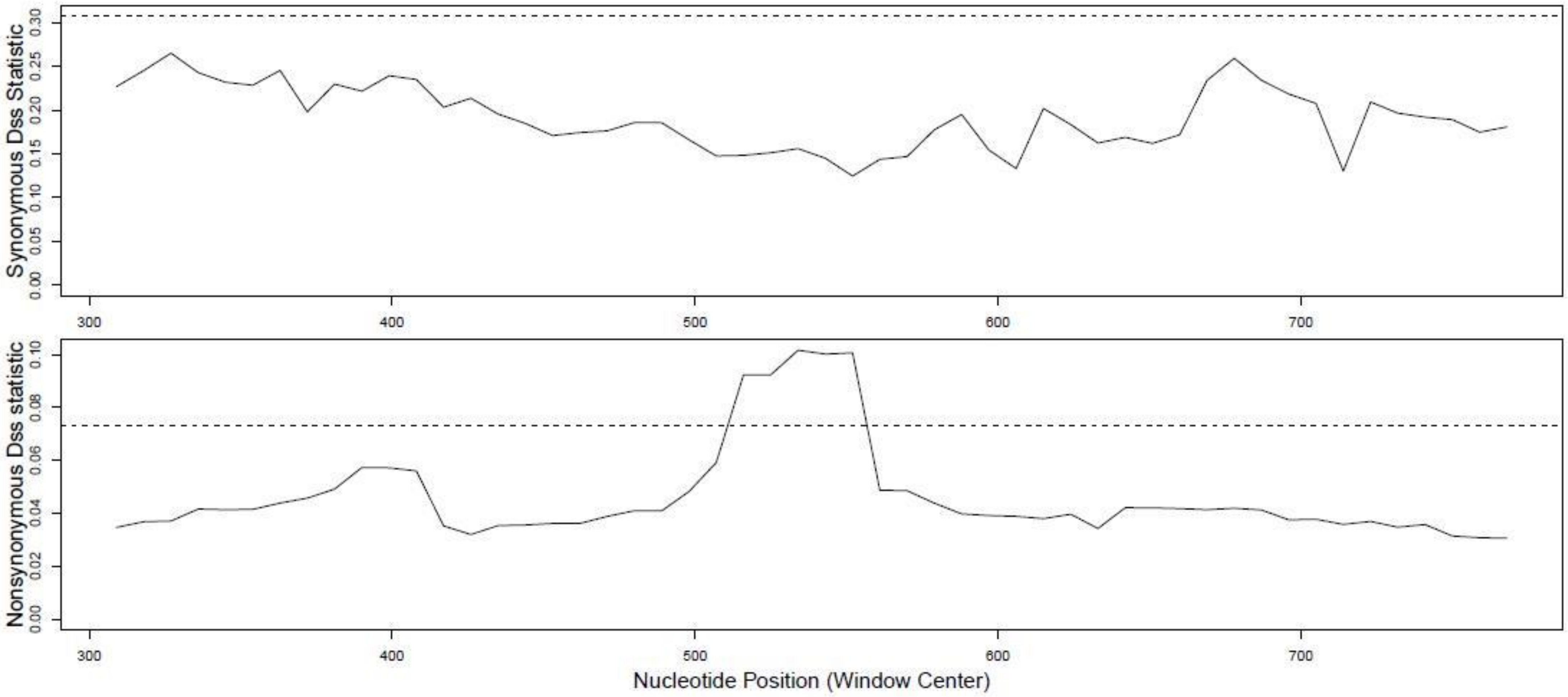

Dss statistic landscapes for synonymous and nonsynonymous changes. Dotted horizontal lines represent the 95% significance level for each test, from a parametric bootstrap with 500 replicates.

(A) 25 randomly selected core genes designated as **recombinants** by PhiPack (CONTD.)

HP_0563

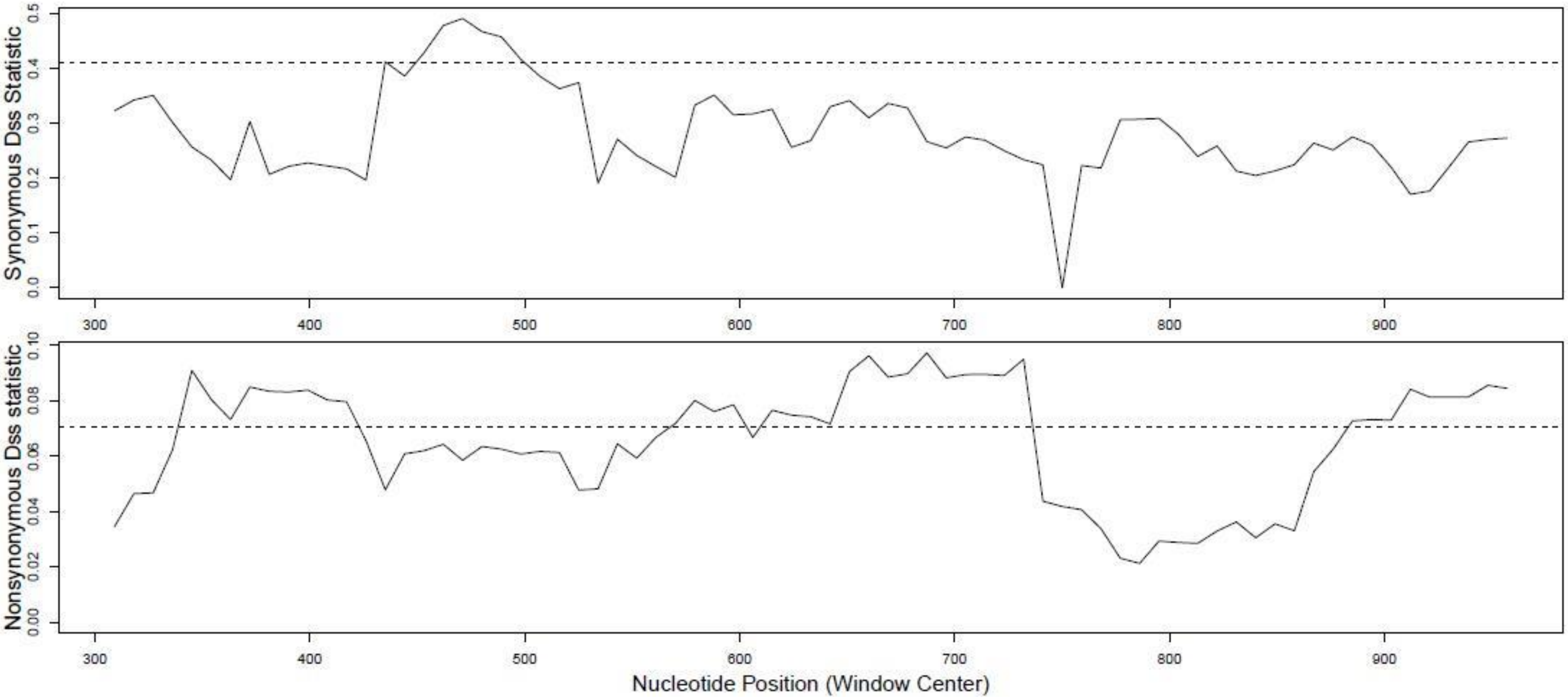

Dss statistic landscapes for synonymous and nonsynonymous changes. Dotted horizontal lines represent the 95% significance level for each test, from a parametric bootstrap with 500 replicates.

(A) 25 randomly selected core genes designated as **recombinants** by PhiPack (CONTD.)

HP_0603

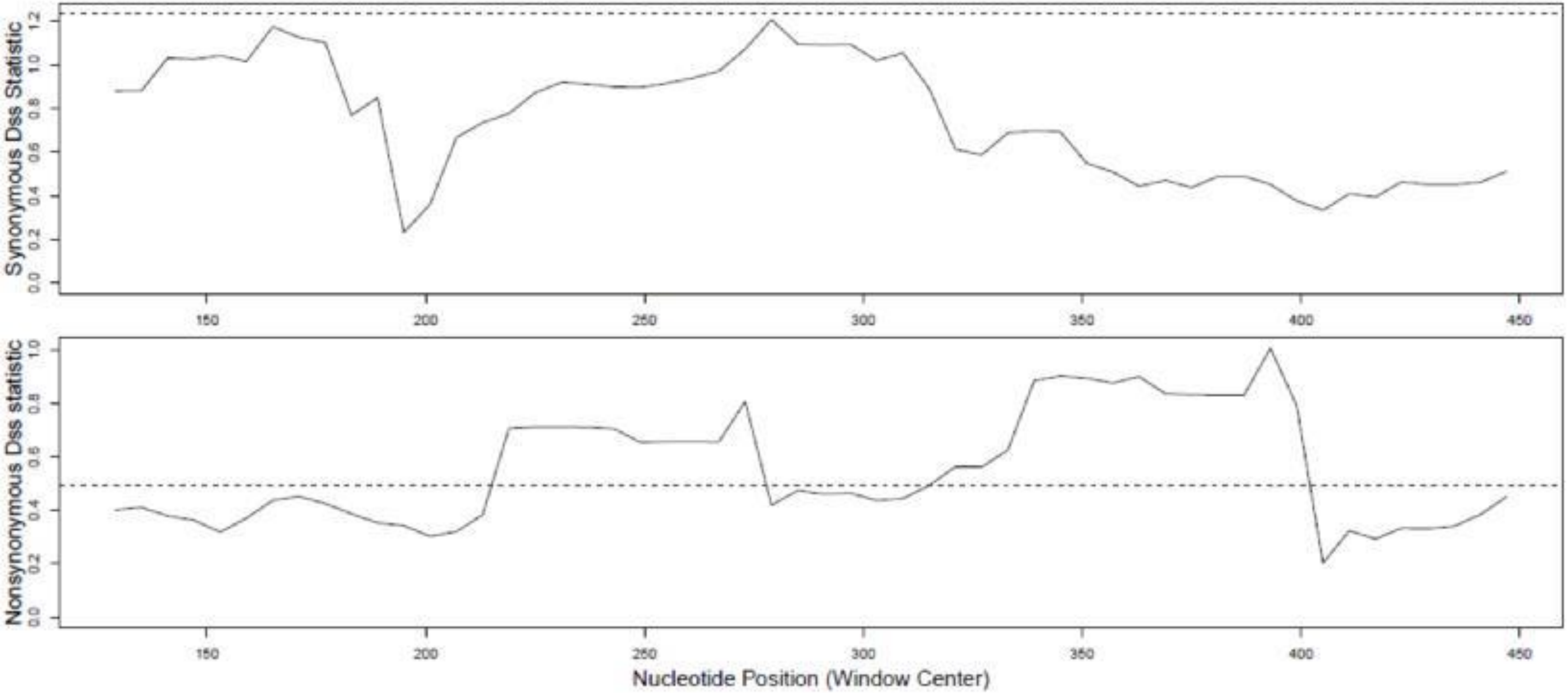

Dss statistic landscapes for synonymous and nonsynonymous changes. Dotted horizontal lines represent the 95% significance level for each test, from a parametric bootstrap with 500 replicates.

(A) 25 randomly selected core genes designated as **recombinants** by PhiPack (CONTD.)

HP_0630

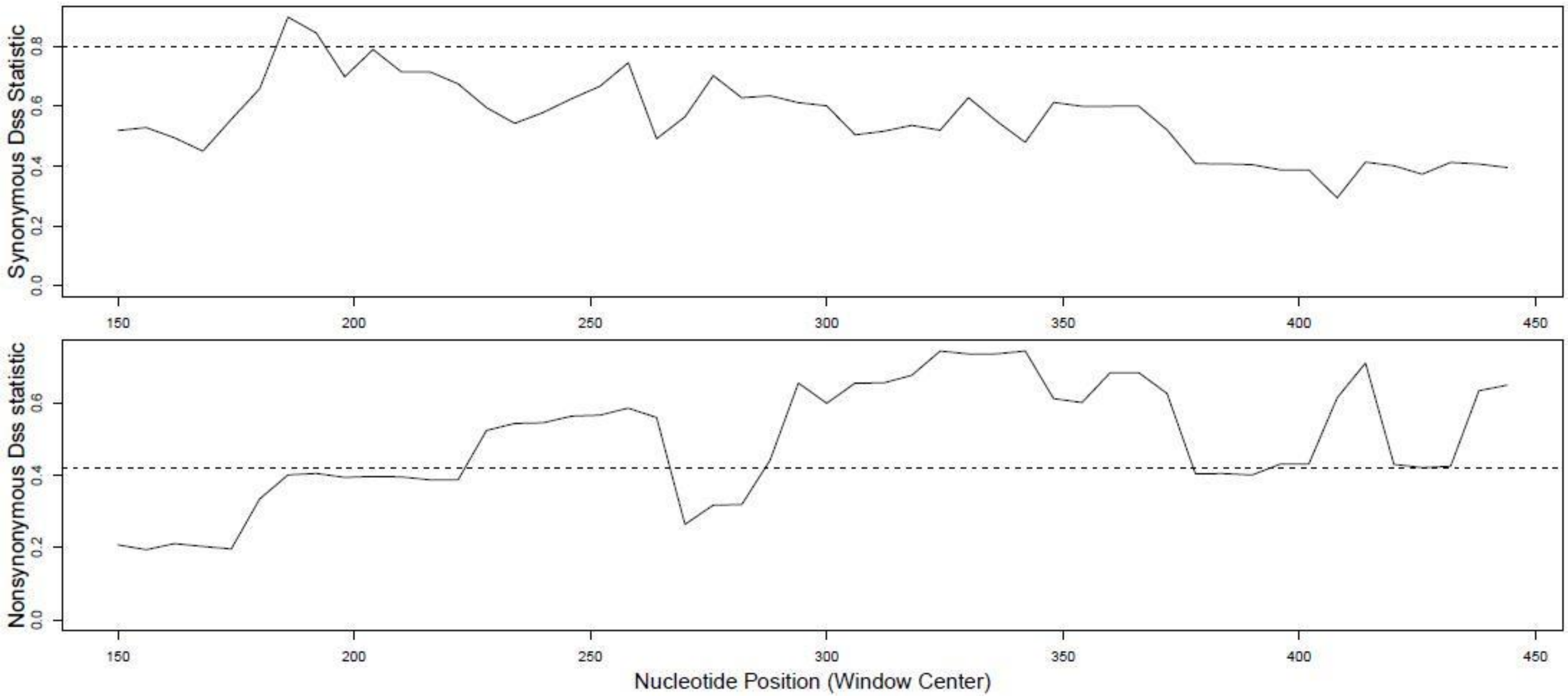

Dss statistic landscapes for synonymous and nonsynonymous changes. Dotted horizontal lines represent the 95% significance level for each test, from a parametric bootstrap with 500 replicates.

(A) 25 randomly selected core genes designated as **recombinants** by PhiPack (CONTD.)

HP_0683

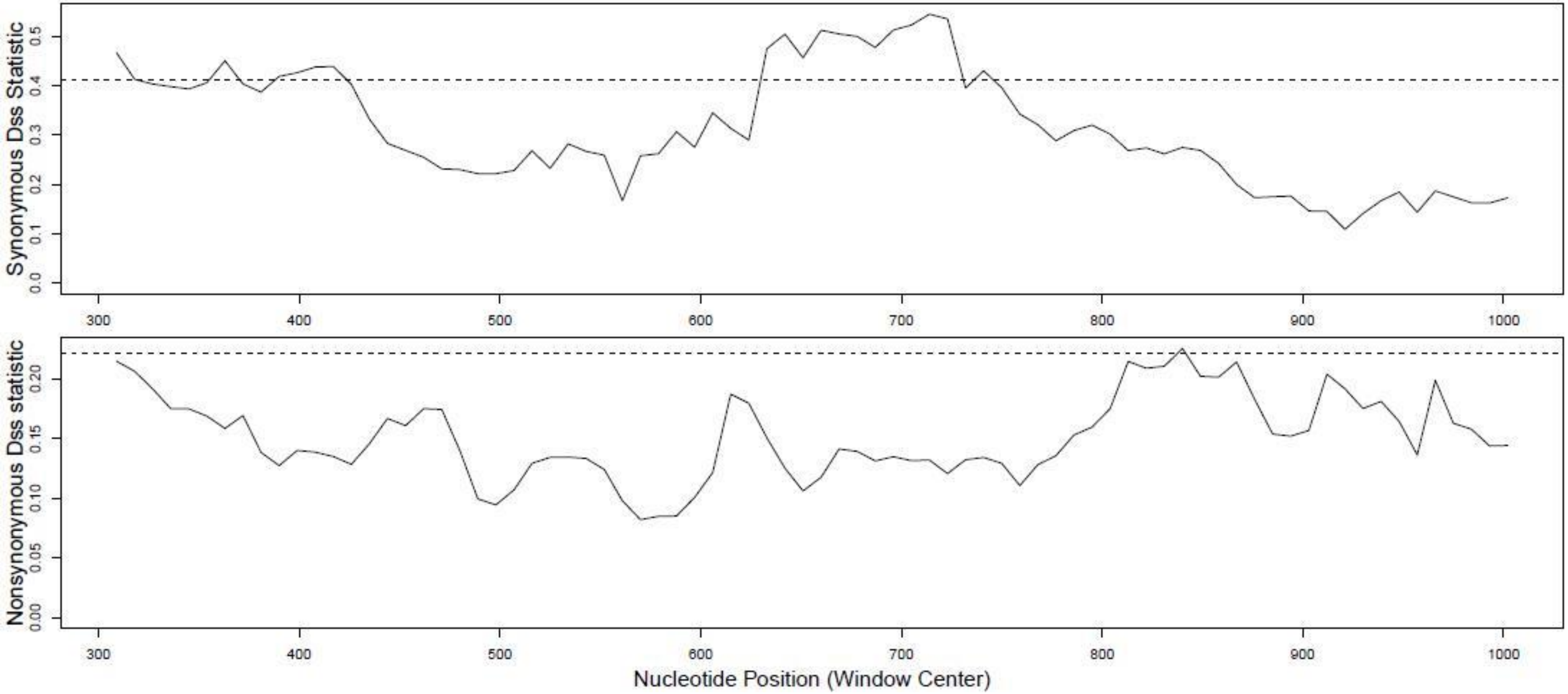

Dss statistic landscapes for synonymous and nonsynonymous changes. Dotted horizontal lines represent the 95% significance level for each test, from a parametric bootstrap with 500 replicates.

(A) 25 randomly selected core genes designated as **recombinants** by PhiPack (CONTD.)

HP_0747

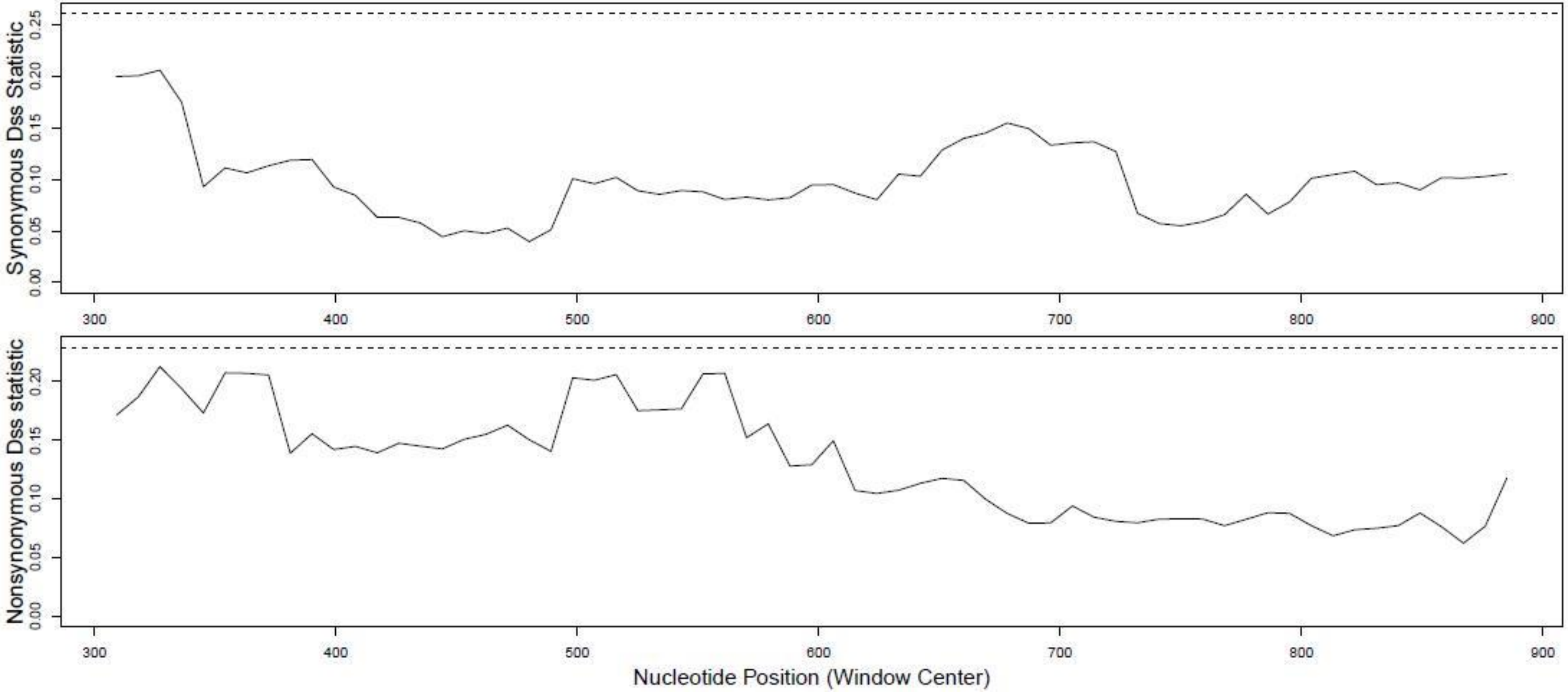

Dss statistic landscapes for synonymous and nonsynonymous changes. Dotted horizontal lines represent the 95% significance level for each test, from a parametric bootstrap with 500 replicates.

(A) 25 randomly selected core genes designated as **recombinants** by PhiPack (CONTD.)

HP_0865

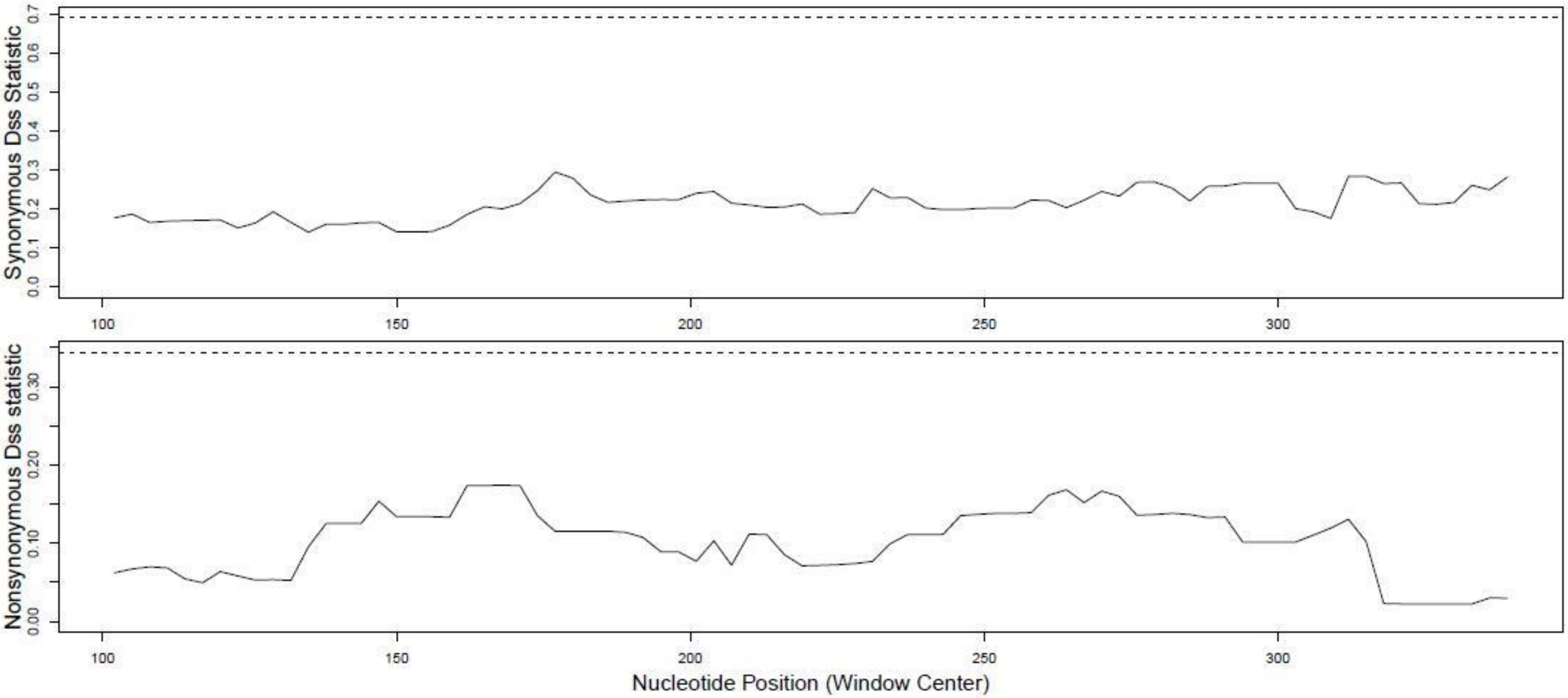

Dss statistic landscapes for synonymous and nonsynonymous changes. Dotted horizontal lines represent the 95% significance level for each test, from a parametric bootstrap with 500 replicates.

(A) 25 randomly selected core genes designated as **recombinants** by PhiPack (CONTD.)

HP_0959

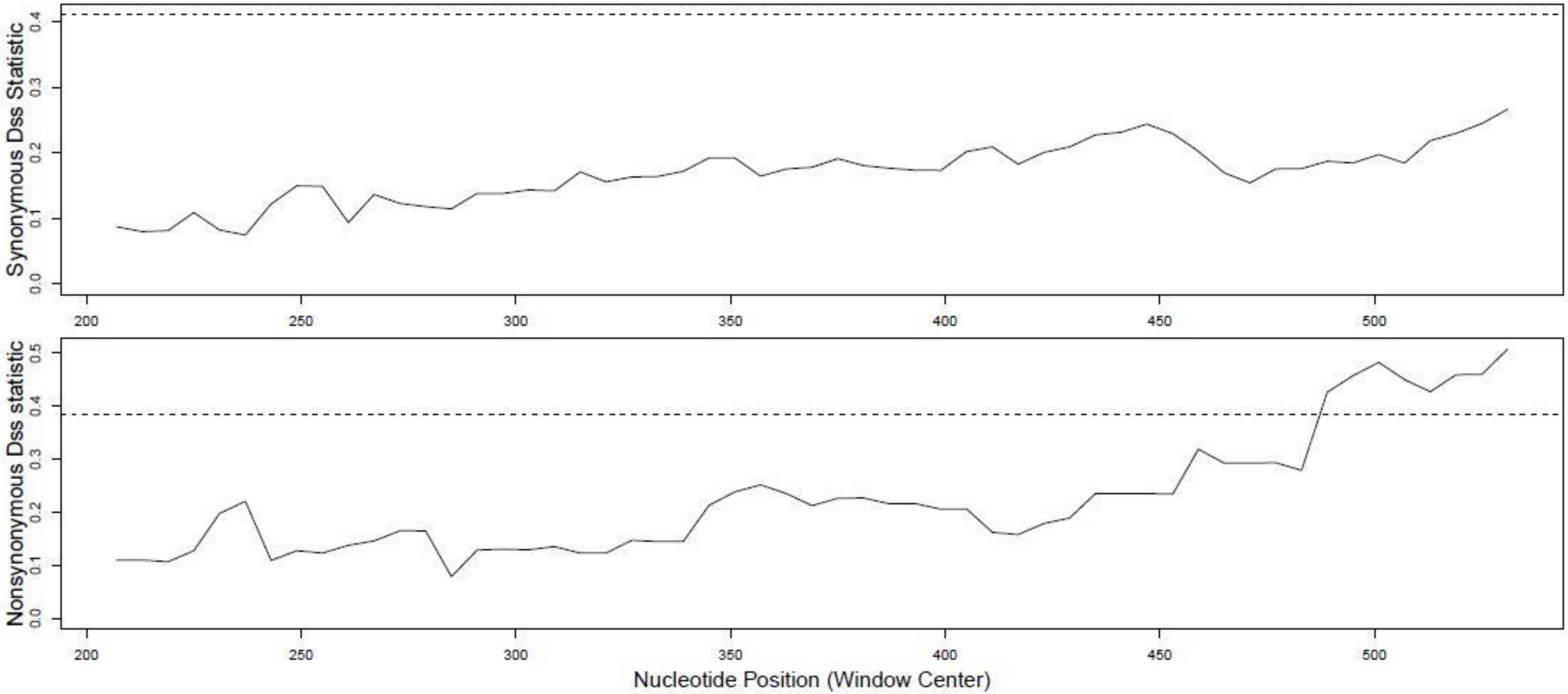

Dss statistic landscapes for synonymous and nonsynonymous changes. Dotted horizontal lines represent the 95% significance level for each test, from a parametric bootstrap with 500 replicates.

(A) 25 randomly selected core genes designated as **recombinants** by PhiPack (CONTD.)

HP_1014

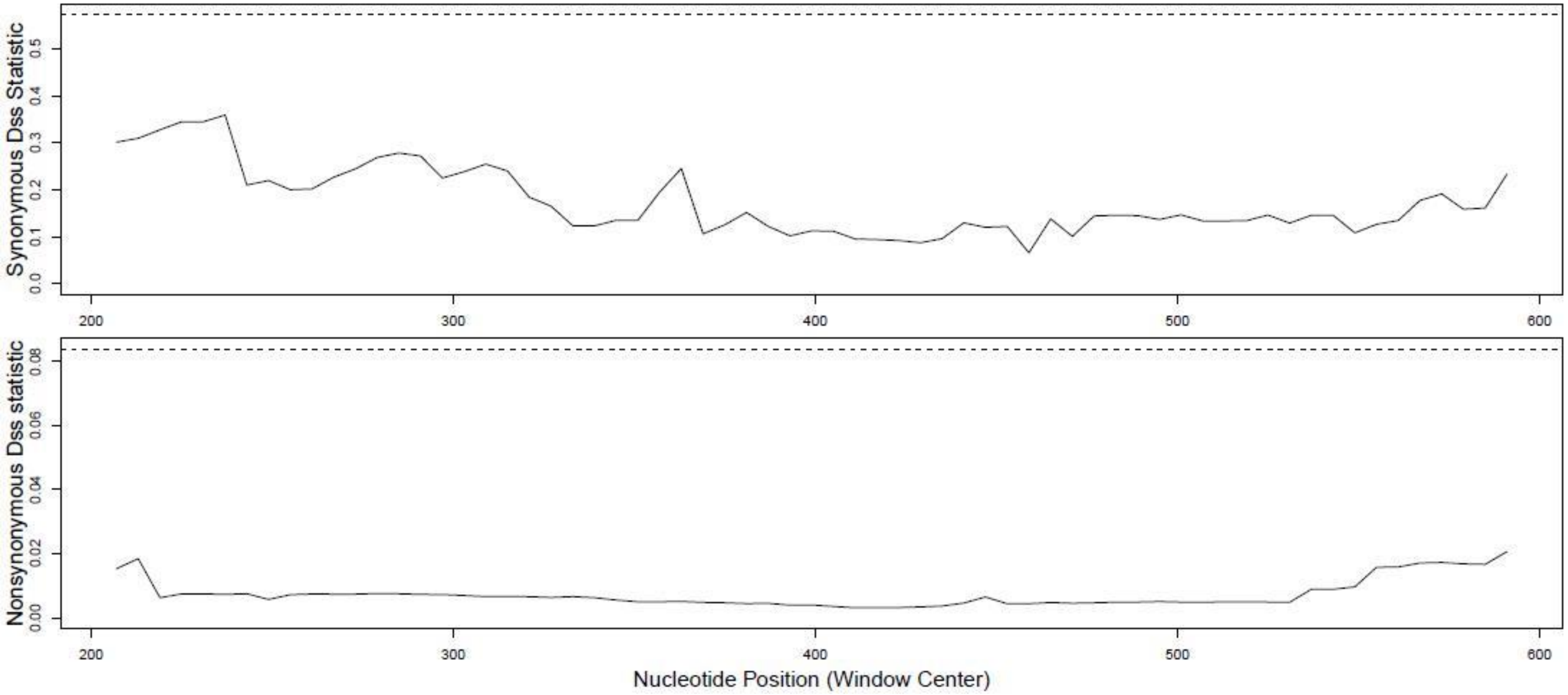

Dss statistic landscapes for synonymous and nonsynonymous changes. Dotted horizontal lines represent the 95% significance level for each test, from a parametric bootstrap with 500 replicates.

(A) 25 randomly selected core genes designated as **recombinants** by PhiPack (CONTD.)

HP_1029

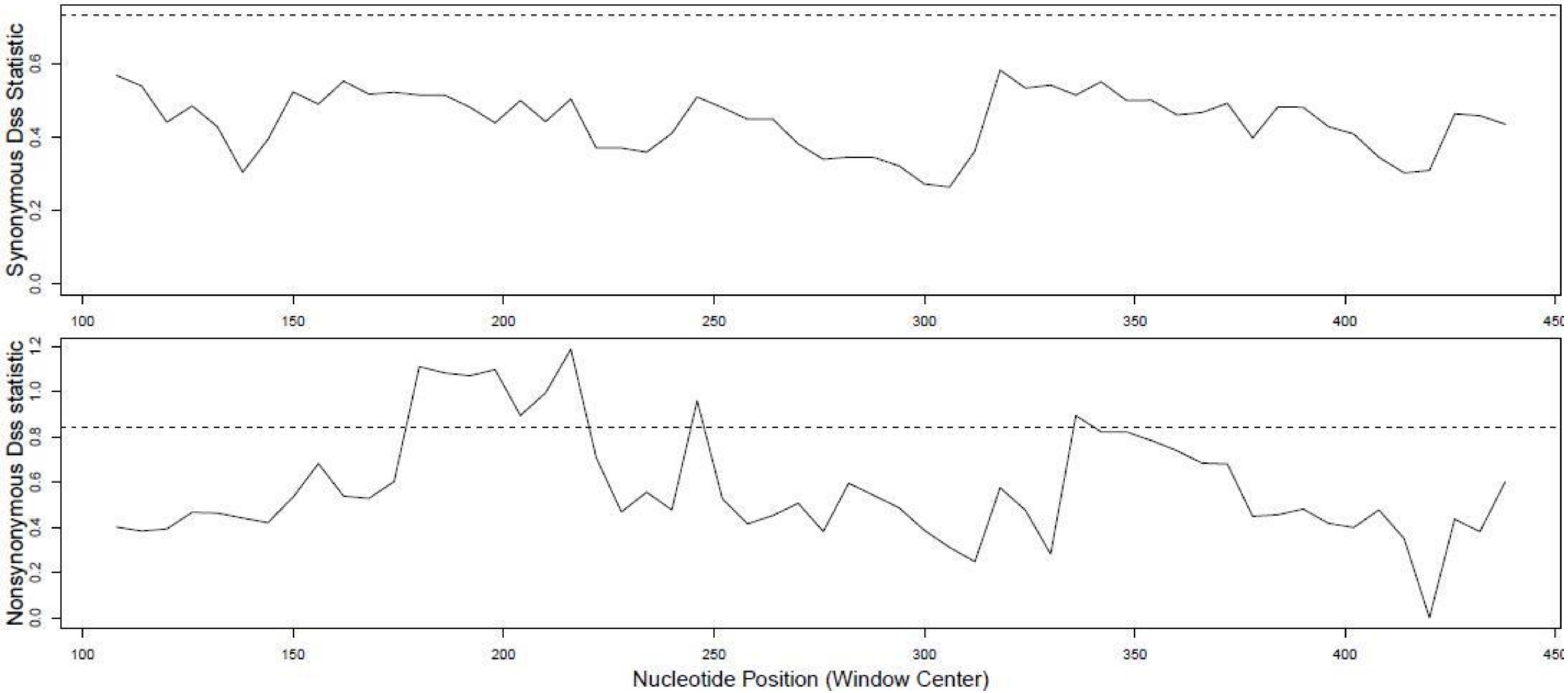

Dss statistic landscapes for synonymous and nonsynonymous changes. Dotted horizontal lines represent the 95% significance level for each test, from a parametric bootstrap with 500 replicates.

(A) 25 randomly selected core genes designated as **recombinants** by PhiPack (CONTD.)

HP_1055

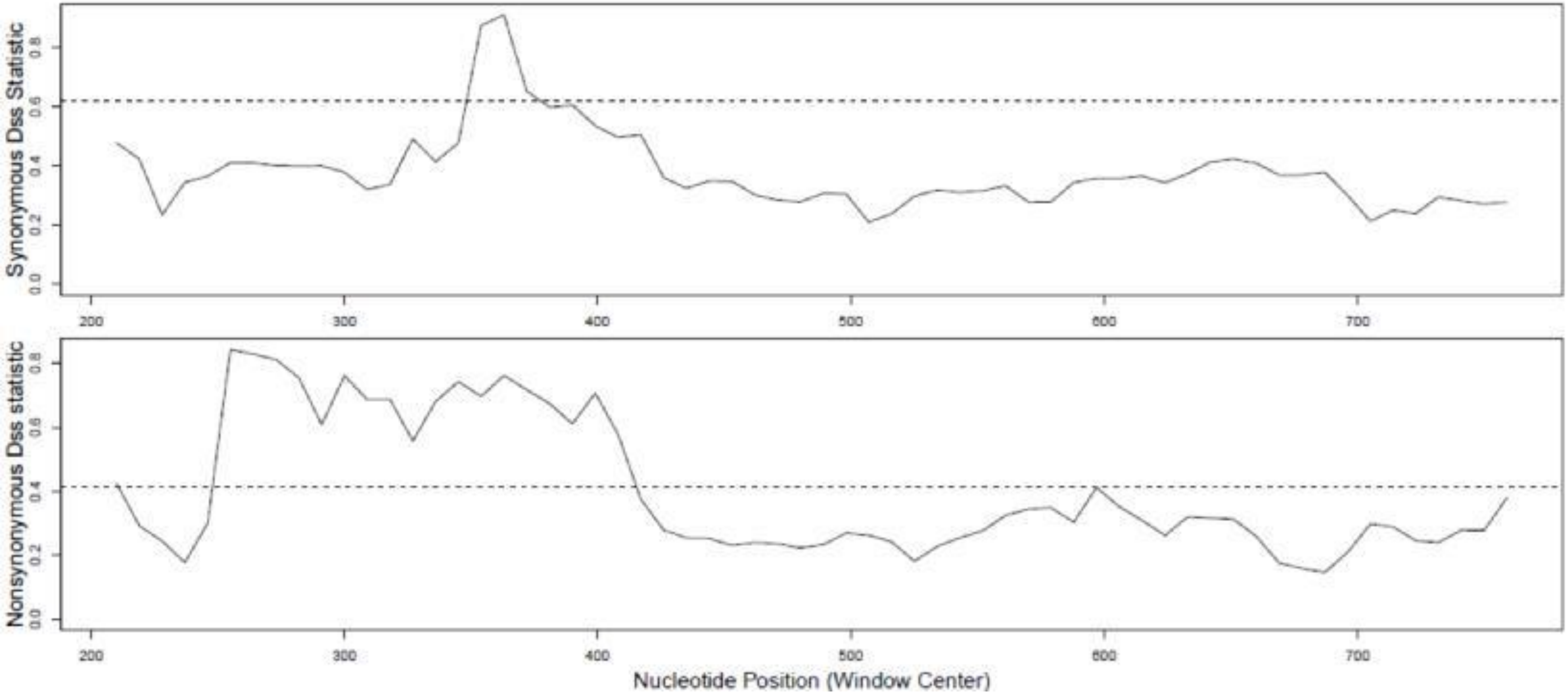

Dss statistic landscapes for synonymous and nonsynonymous changes. Dotted horizontal lines represent the 95% significance level for each test, from a parametric bootstrap with 500 replicates.

(A) 25 randomly selected core genes designated as **recombinants** by PhiPack (CONTD.)

HP_1112

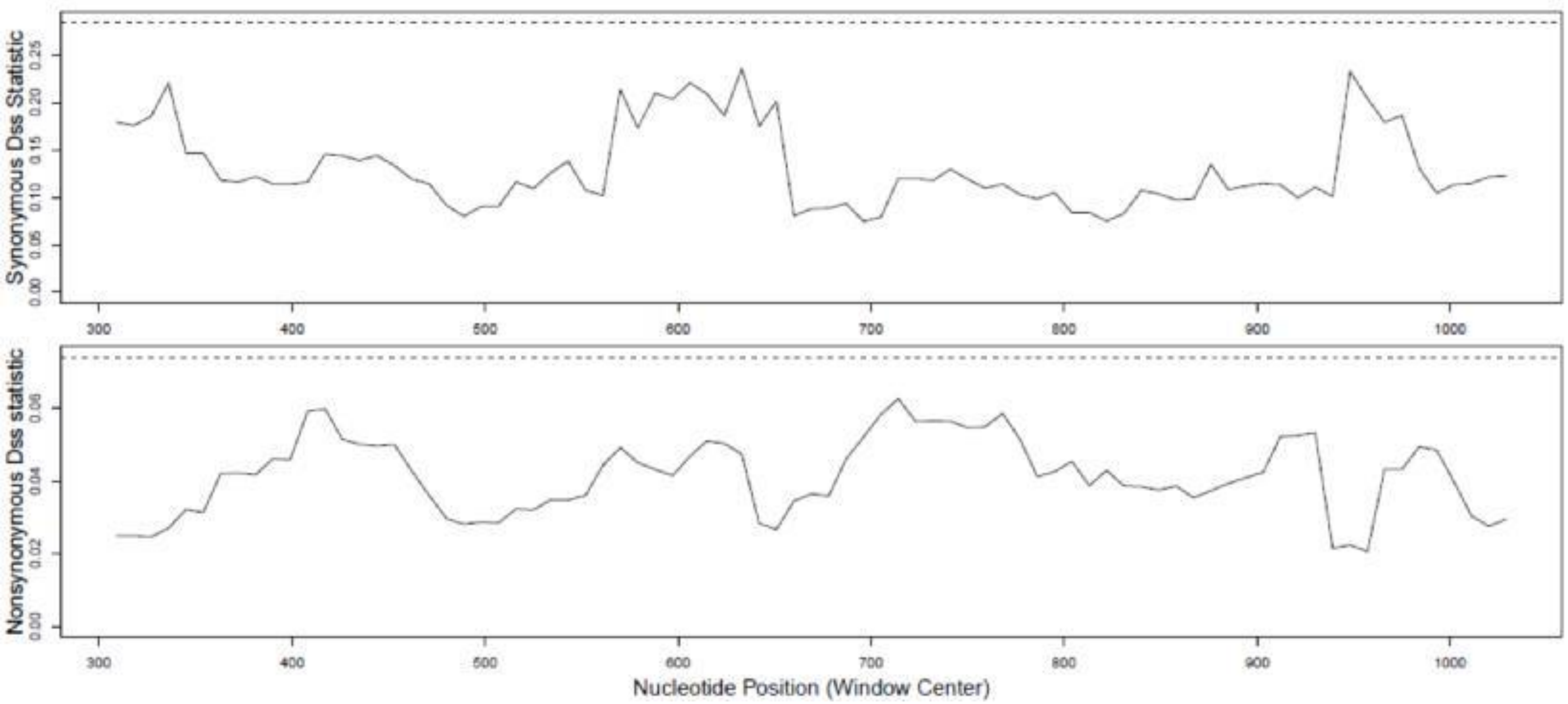

Dss statistic landscapes for synonymous and nonsynonymous changes. Dotted horizontal lines represent the 95% significance level for each test, from a parametric bootstrap with 500 replicates.

(A) 25 randomly selected core genes designated as **recombinants** by PhiPack (CONTD.)

HP_1121

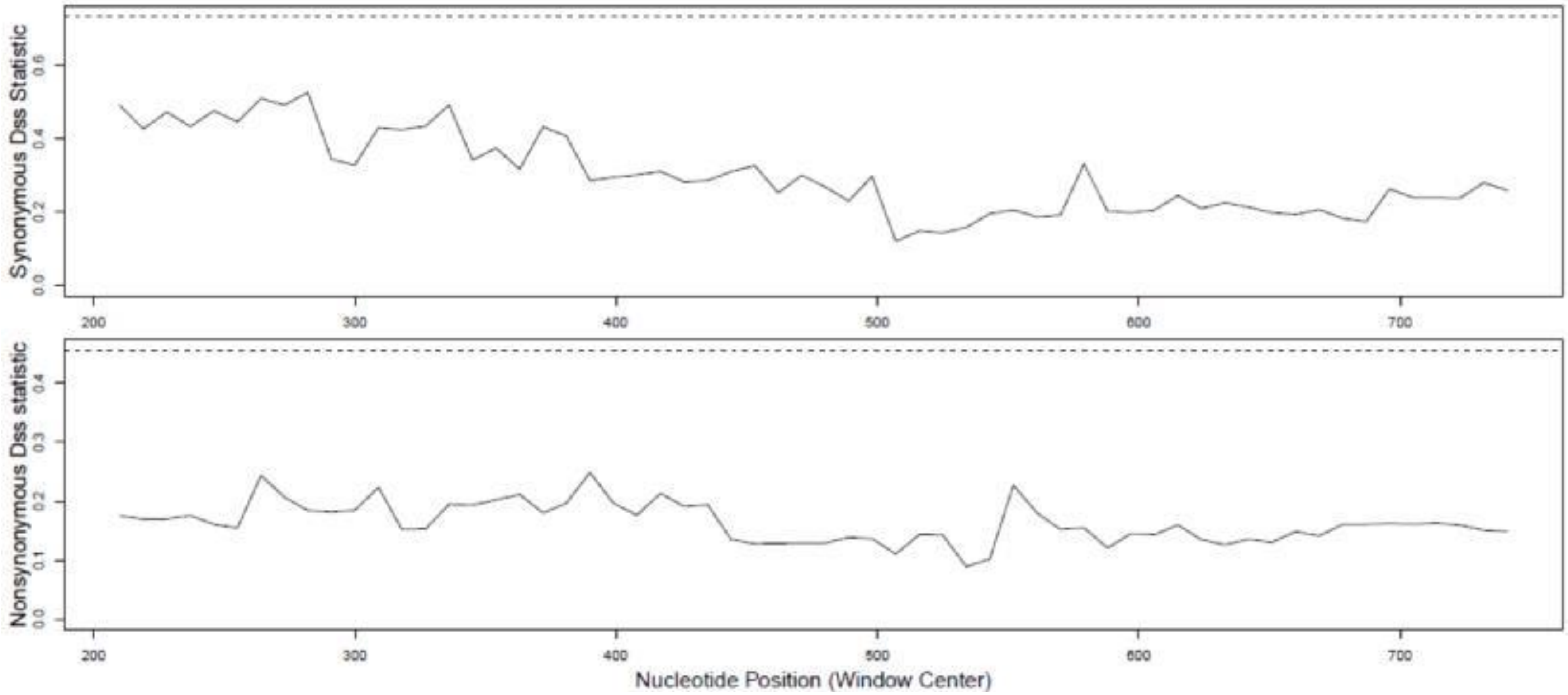

Dss statistic landscapes for synonymous and nonsynonymous changes. Dotted horizontal lines represent the 95% significance level for each test, from a parametric bootstrap with 500 replicates.

(A) 25 randomly selected core genes designated as **recombinants** by PhiPack (CONTD.)

HP_1232

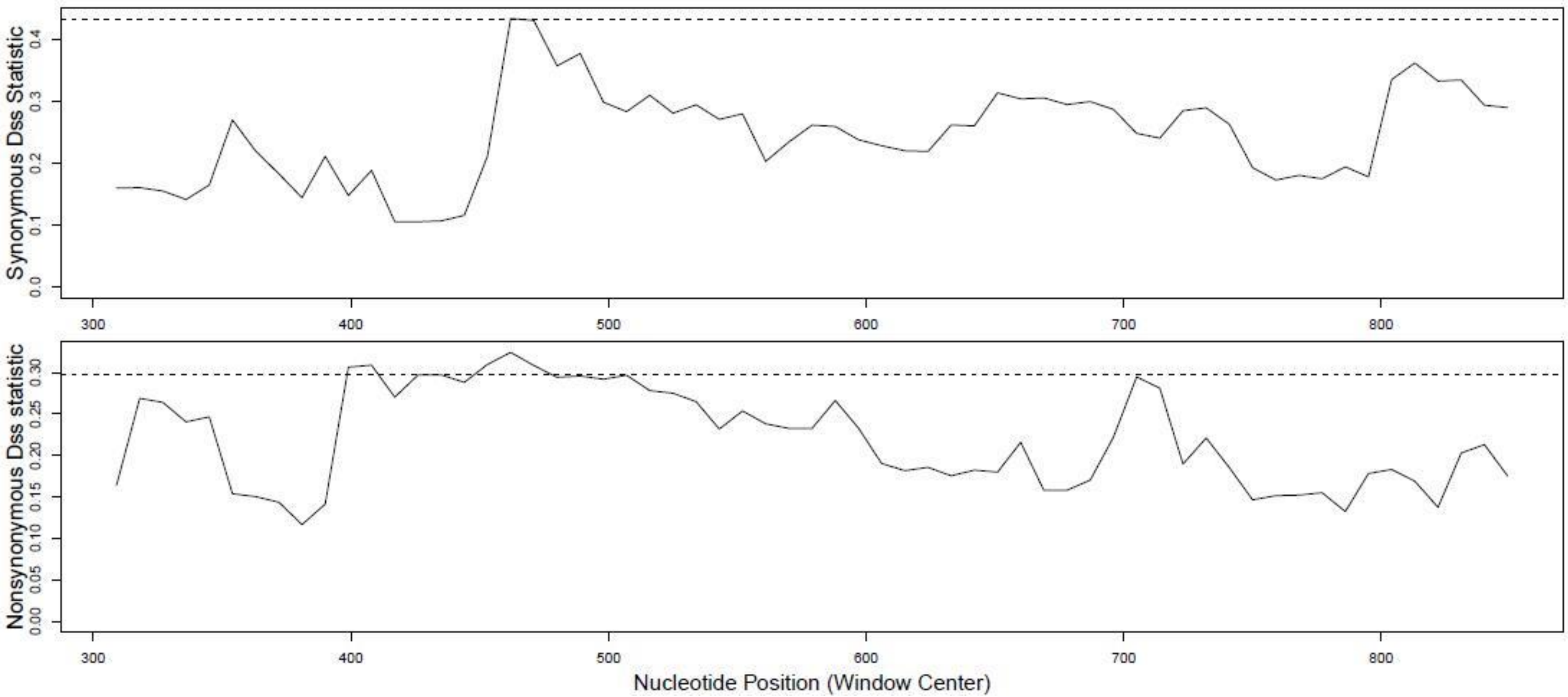

Dss statistic landscapes for synonymous and nonsynonymous changes. Dotted horizontal lines represent the 95% significance level for each test, from a parametric bootstrap with 500 replicates.

(A) 25 randomly selected core genes designated as **recombinants** by PhiPack (CONTD.)

HP_1268

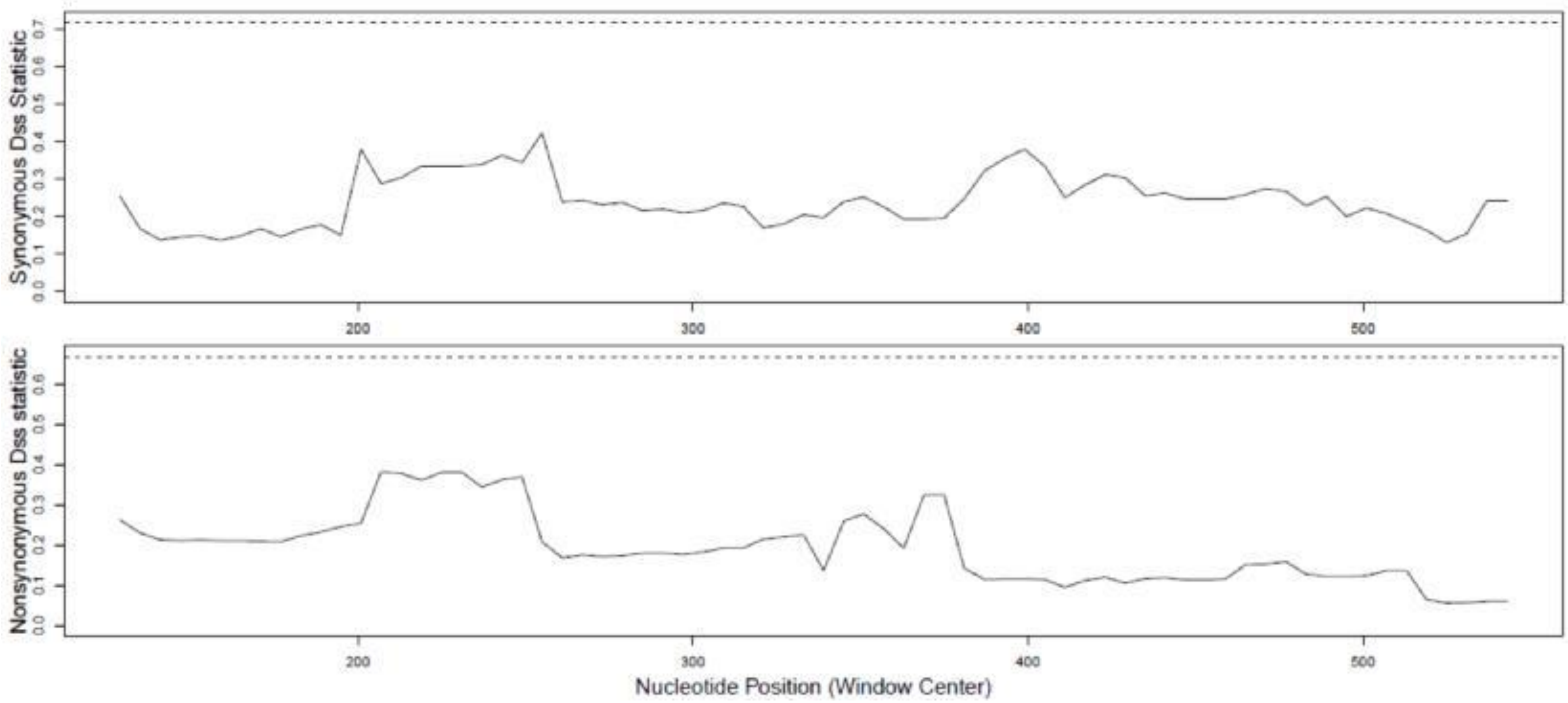

Dss statistic landscapes for synonymous and nonsynonymous changes. Dotted horizontal lines represent the 95% significance level for each test, from a parametric bootstrap with 500 replicates.

(A) 25 randomly selected core genes designated as **recombinants** by PhiPack (CONTD.)

HP_1508

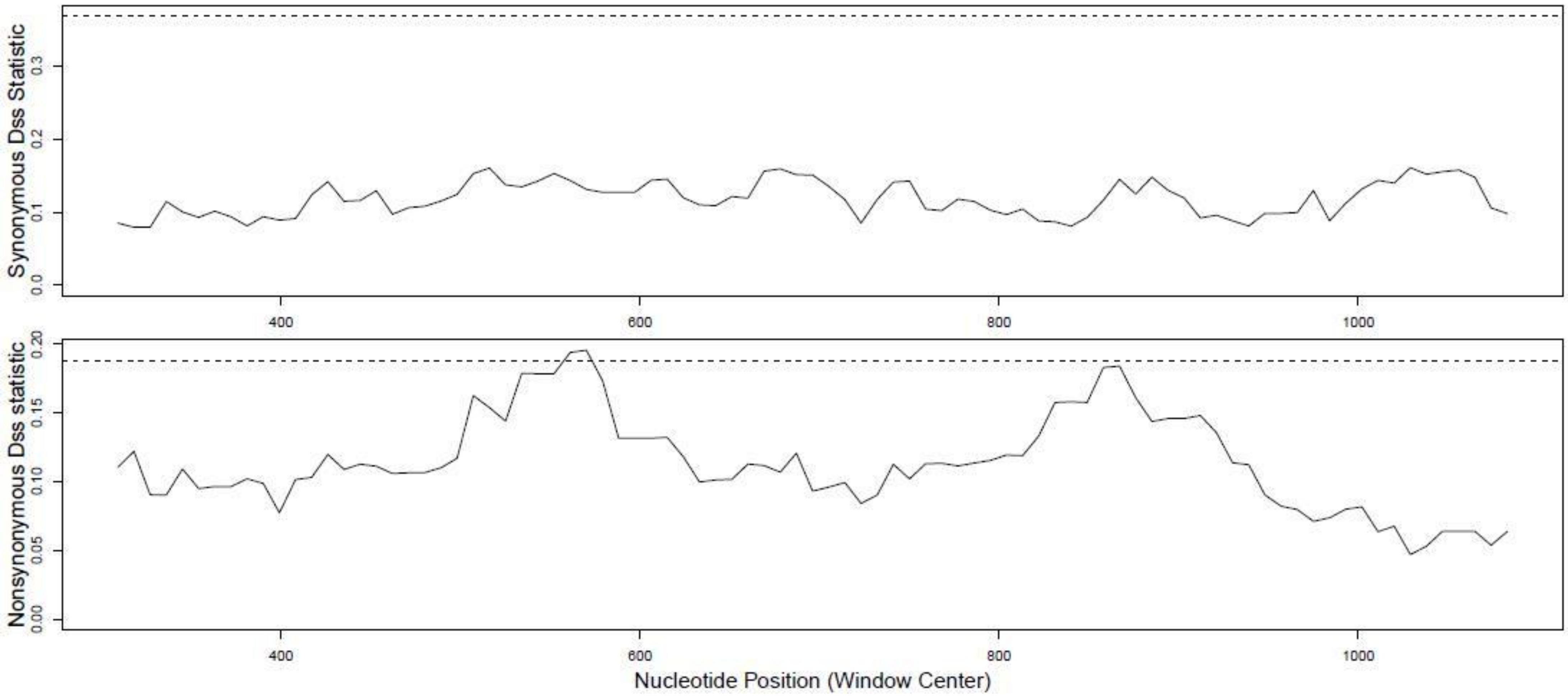

Dss statistic landscapes for synonymous and nonsynonymous changes. Dotted horizontal lines represent the 95% significance level for each test, from a parametric bootstrap with 500 replicates.

(A) 25 randomly selected core genes designated as **recombinants** by PhiPack (CONTD.)

HP_1529

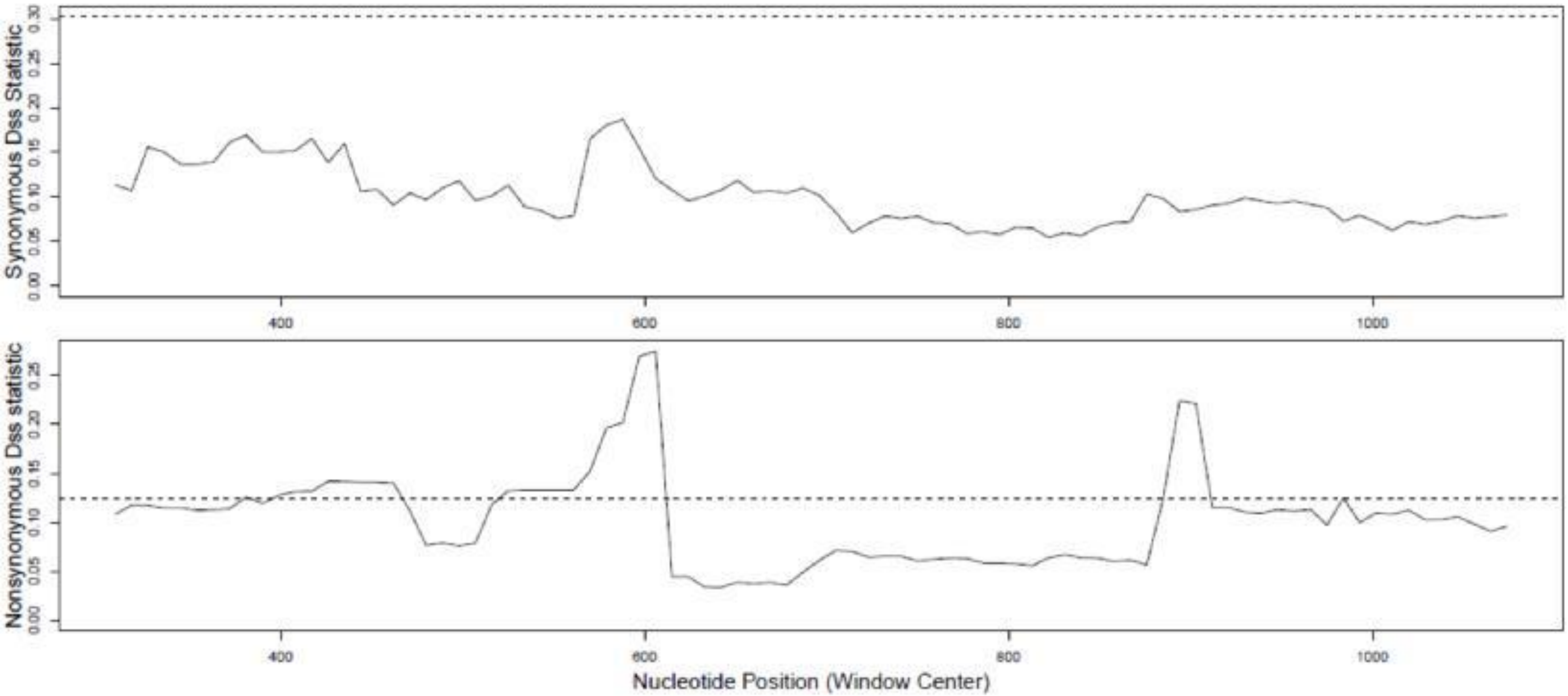

Dss statistic landscapes for synonymous and nonsynonymous changes. Dotted horizontal lines represent the 95% significance level for each test, from a parametric bootstrap with 500 replicates.

(B) 25 randomly selected core genes designated as **non-recombinants** by PhiPack

HP_0020

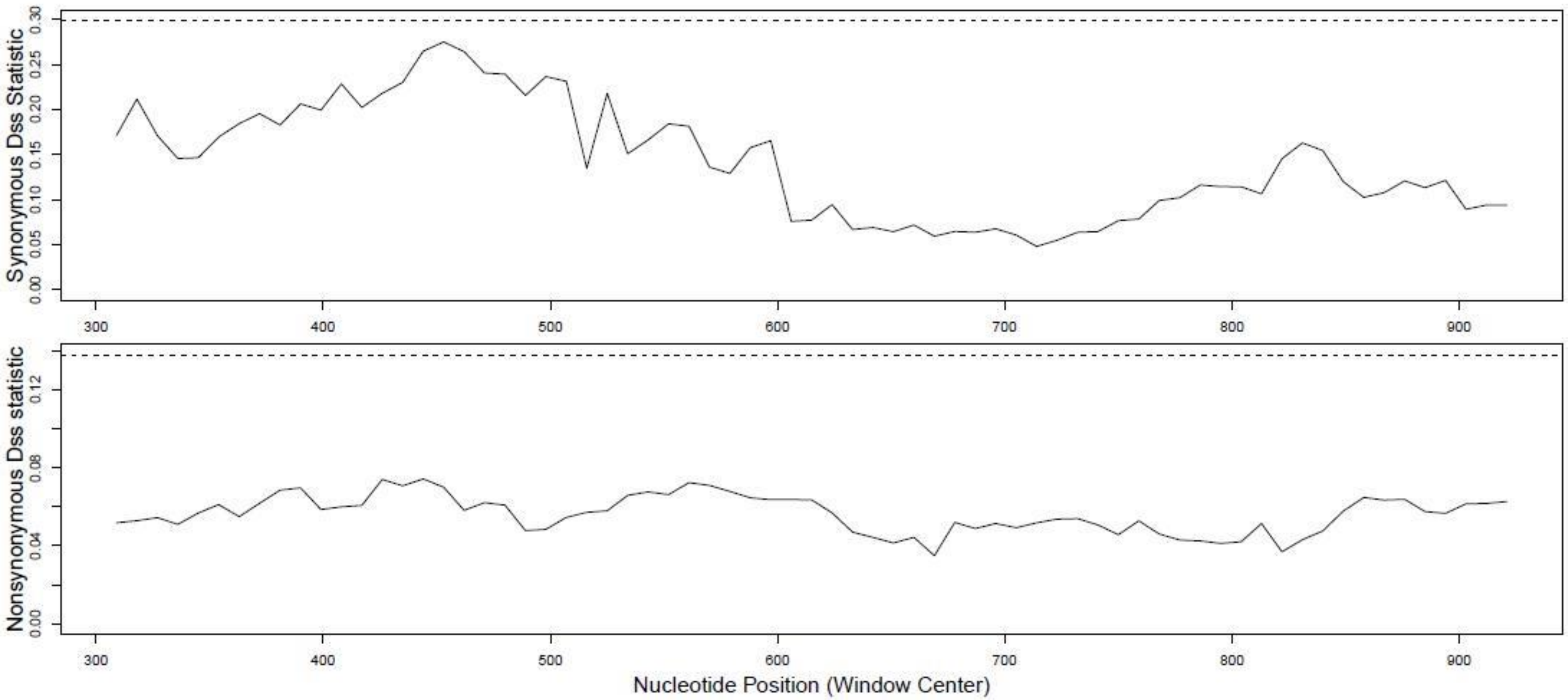

Dss statistic landscapes for synonymous and nonsynonymous changes. Dotted horizontal lines represent the 95% significance level for each test, from a parametric bootstrap with 500 replicates.

(B) 25 randomly selected core genes designated as **non-recombinants** by PhiPack (CONTD.)

HP_0124

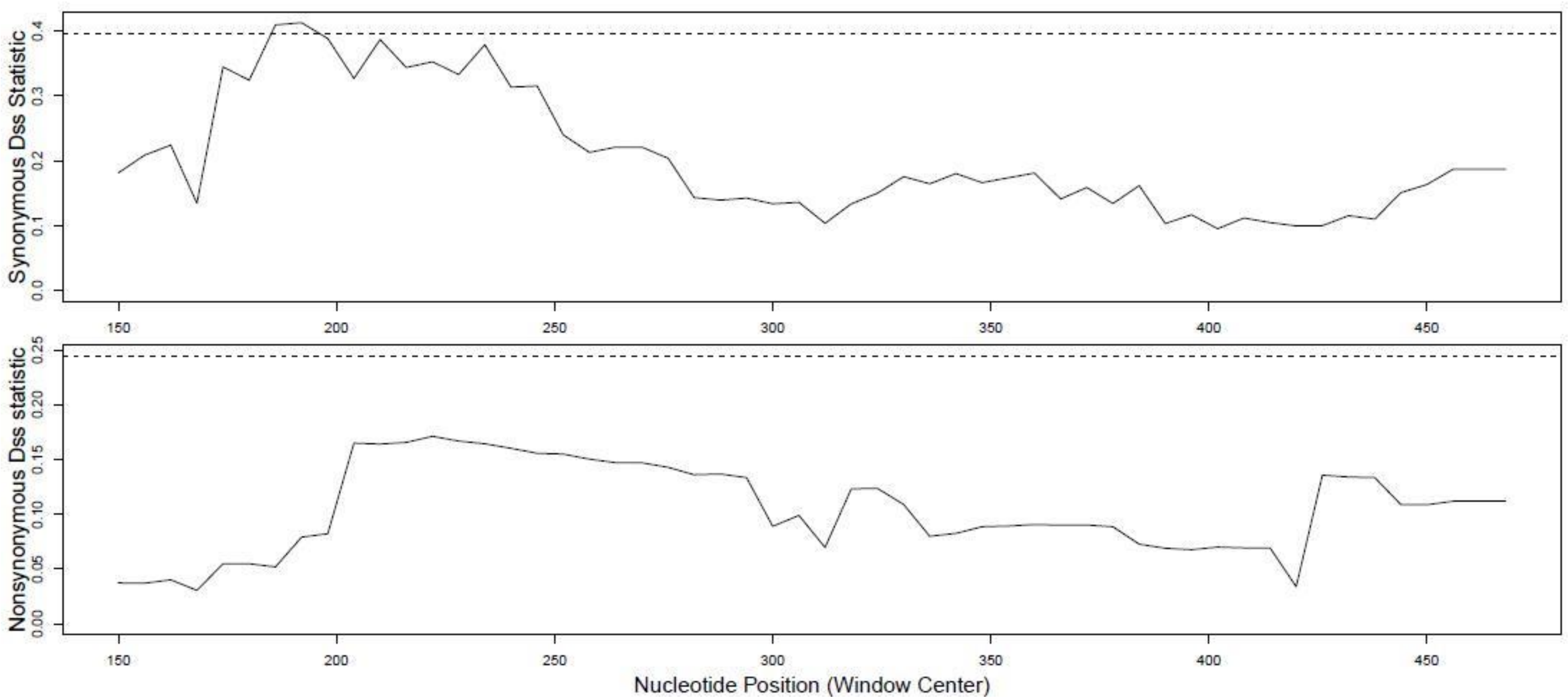

Dss statistic landscapes for synonymous and nonsynonymous changes. Dotted horizontal lines represent the 95% significance level for each test, from a parametric bootstrap with 500 replicates.

(B) 25 randomly selected core genes designated as **non-recombinants** by PhiPack (CONTD.)

HP_0146

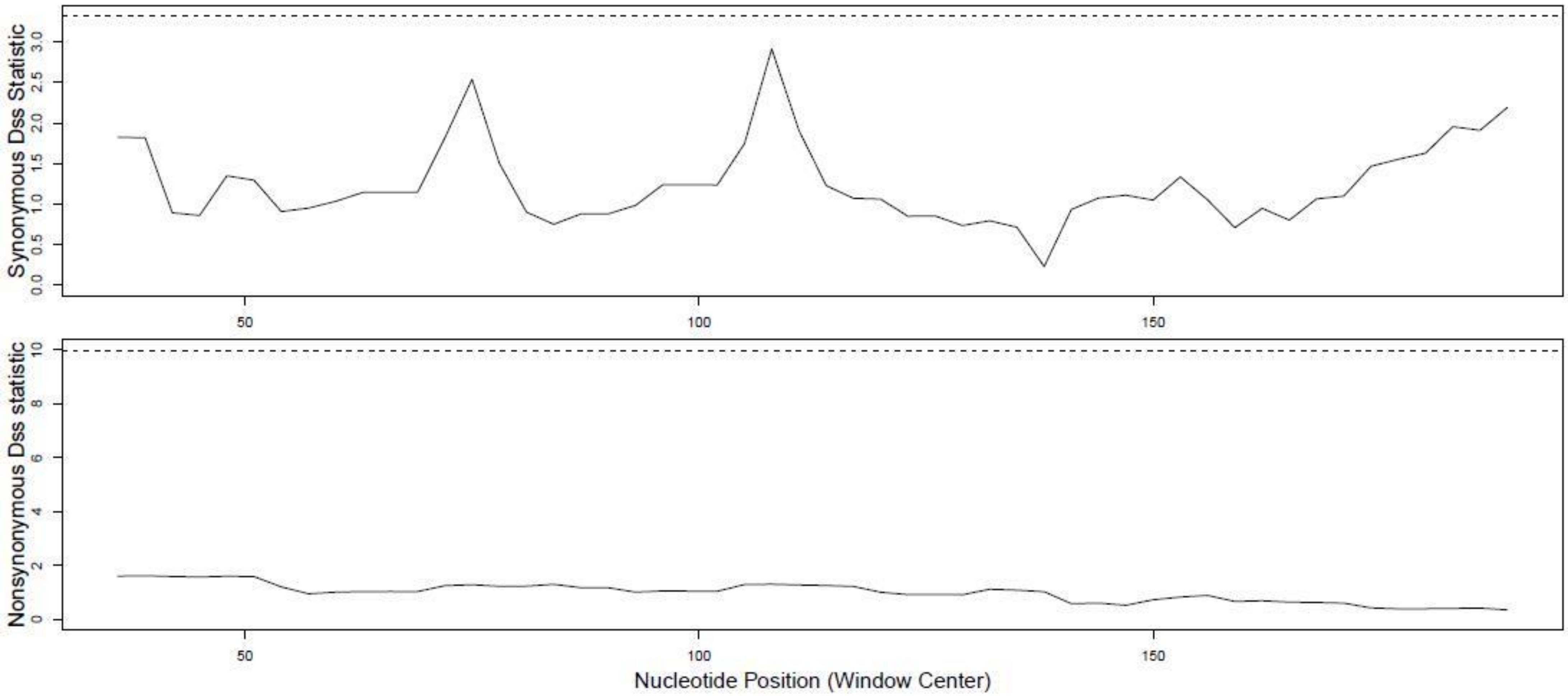

Dss statistic landscapes for synonymous and nonsynonymous changes. Dotted horizontal lines represent the 95% significance level for each test, from a parametric bootstrap with 500 replicates.

(B) 25 randomly selected core genes designated as **non-recombinants** by PhiPack (CONTD.)

HP_0192

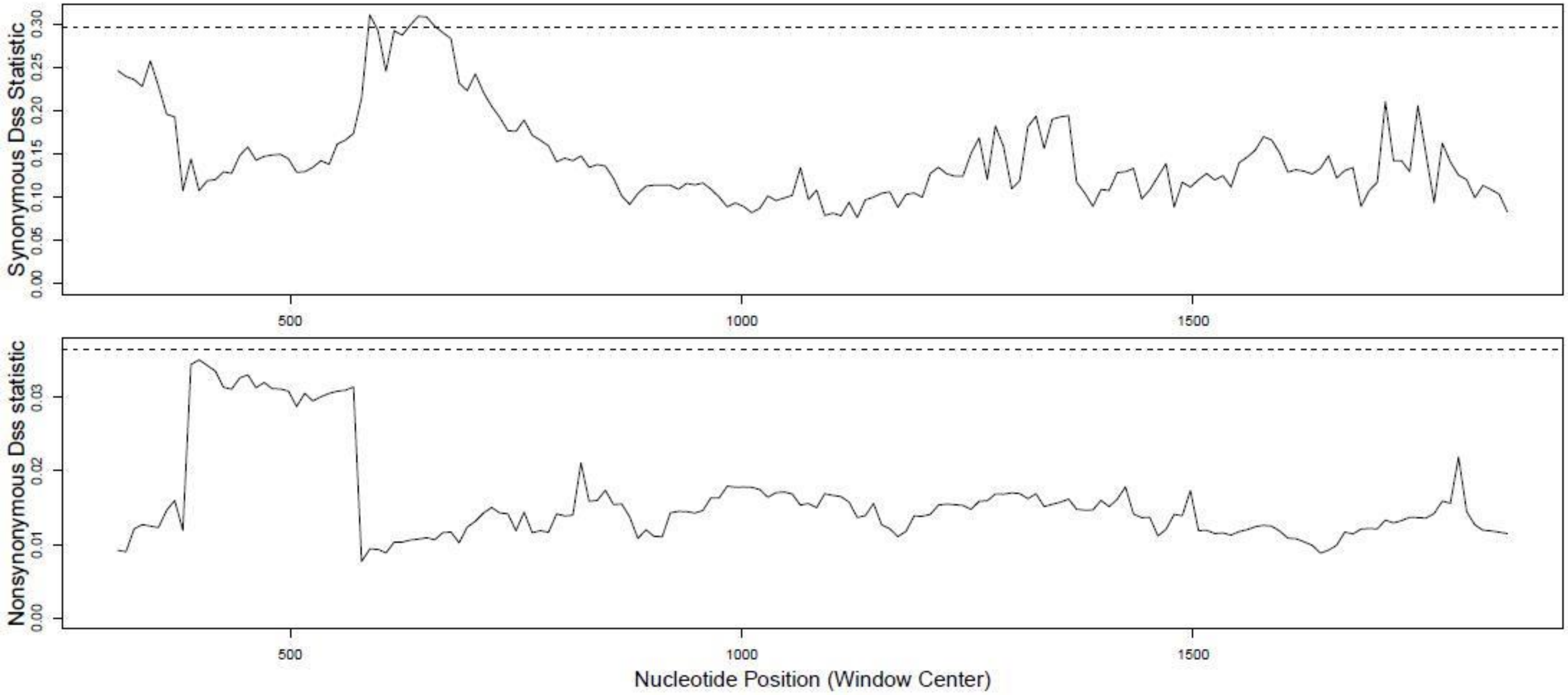

Dss statistic landscapes for synonymous and nonsynonymous changes. Dotted horizontal lines represent the 95% significance level for each test, from a parametric bootstrap with 500 replicates.

(B) 25 randomly selected core genes designated as **non-recombinants** by PhiPack (CONTD.)

HP_0232

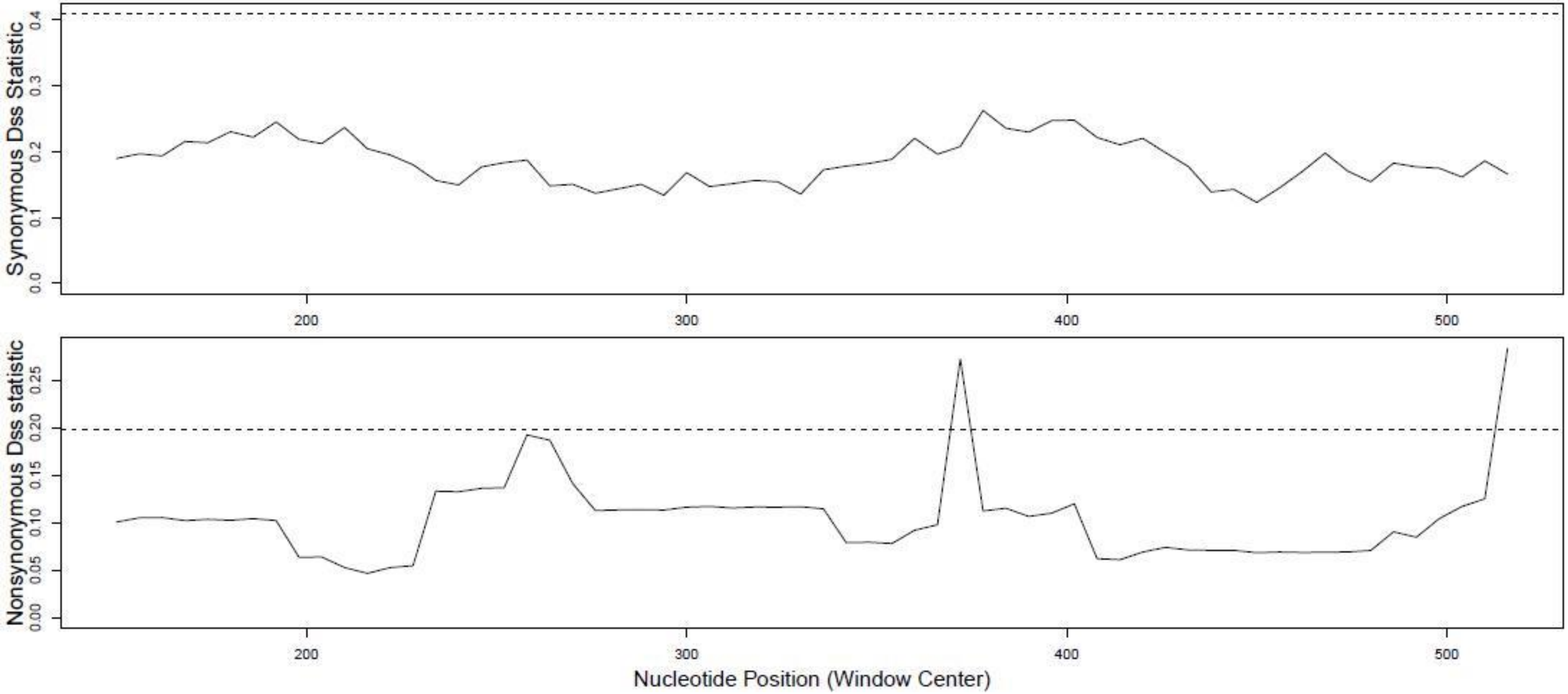

Dss statistic landscapes for synonymous and nonsynonymous changes. Dotted horizontal lines represent the 95% significance level for each test, from a parametric bootstrap with 500 replicates.

(B) 25 randomly selected core genes designated as **non-recombinants** by PhiPack (CONTD.)

HP_0274

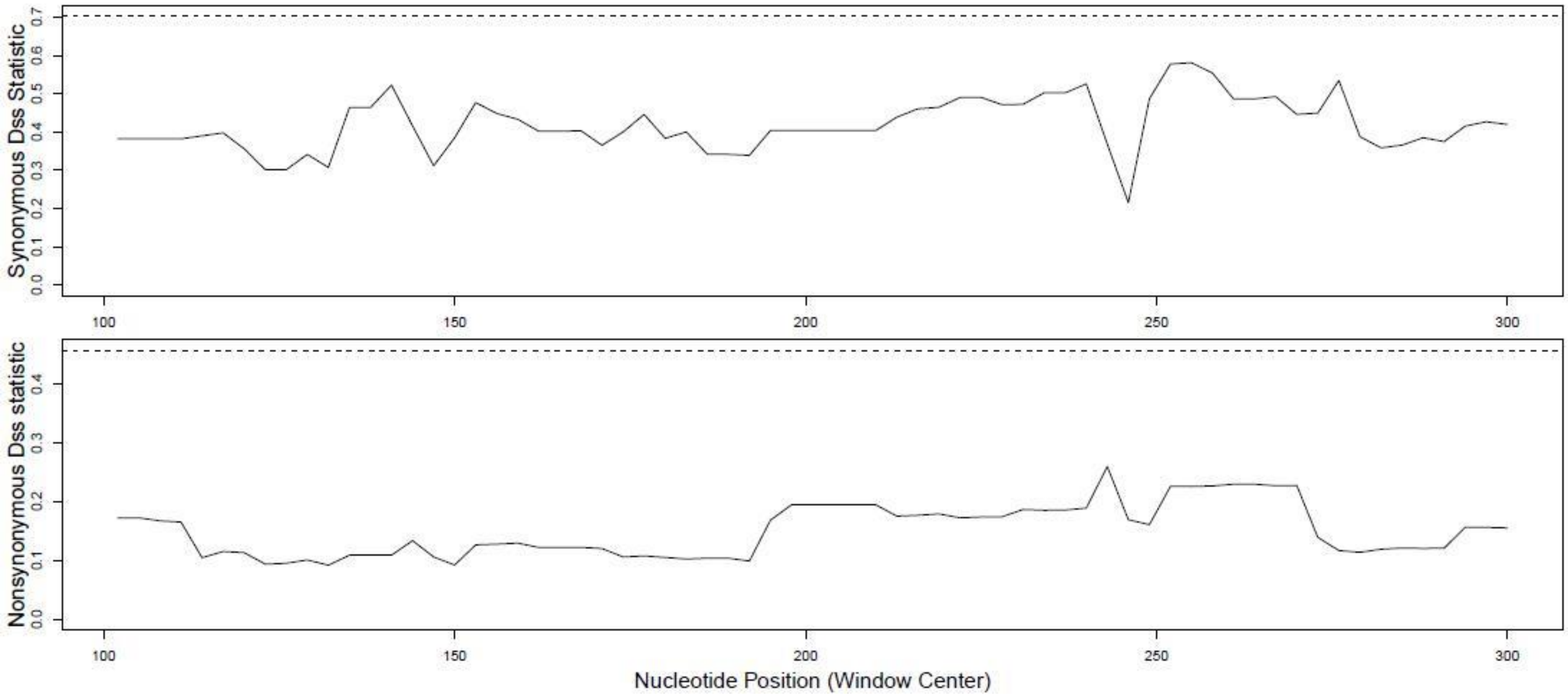

Dss statistic landscapes for synonymous and nonsynonymous changes. Dotted horizontal lines represent the 95% significance level for each test, from a parametric bootstrap with 500 replicates.

(B) 25 randomly selected core genes designated as **non-recombinants** by PhiPack (CONTD.)

HP_0299

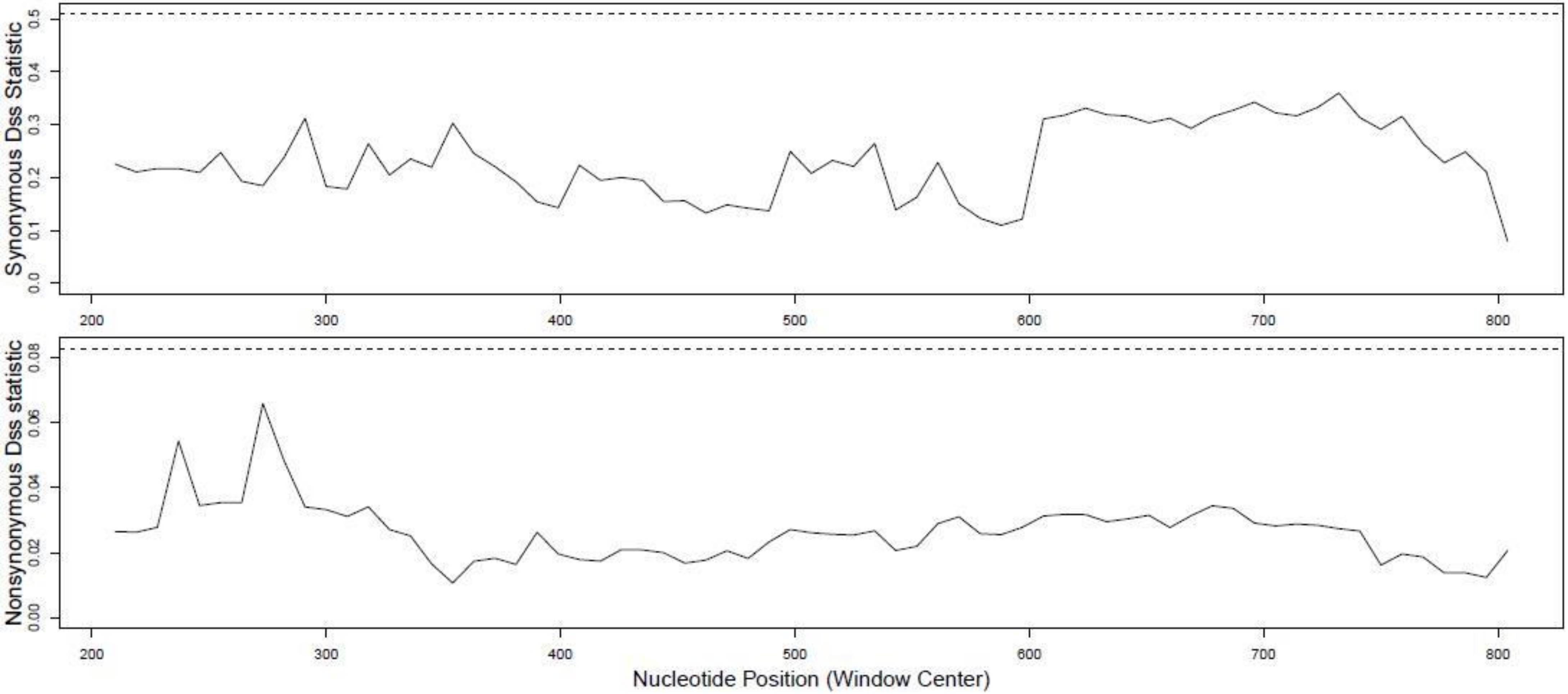

Dss statistic landscapes for synonymous and nonsynonymous changes. Dotted horizontal lines represent the 95% significance level for each test, from a parametric bootstrap with 500 replicates.

(B) 25 randomly selected core genes designated as **non-recombinants** by PhiPack (CONTD.)

HP_0380

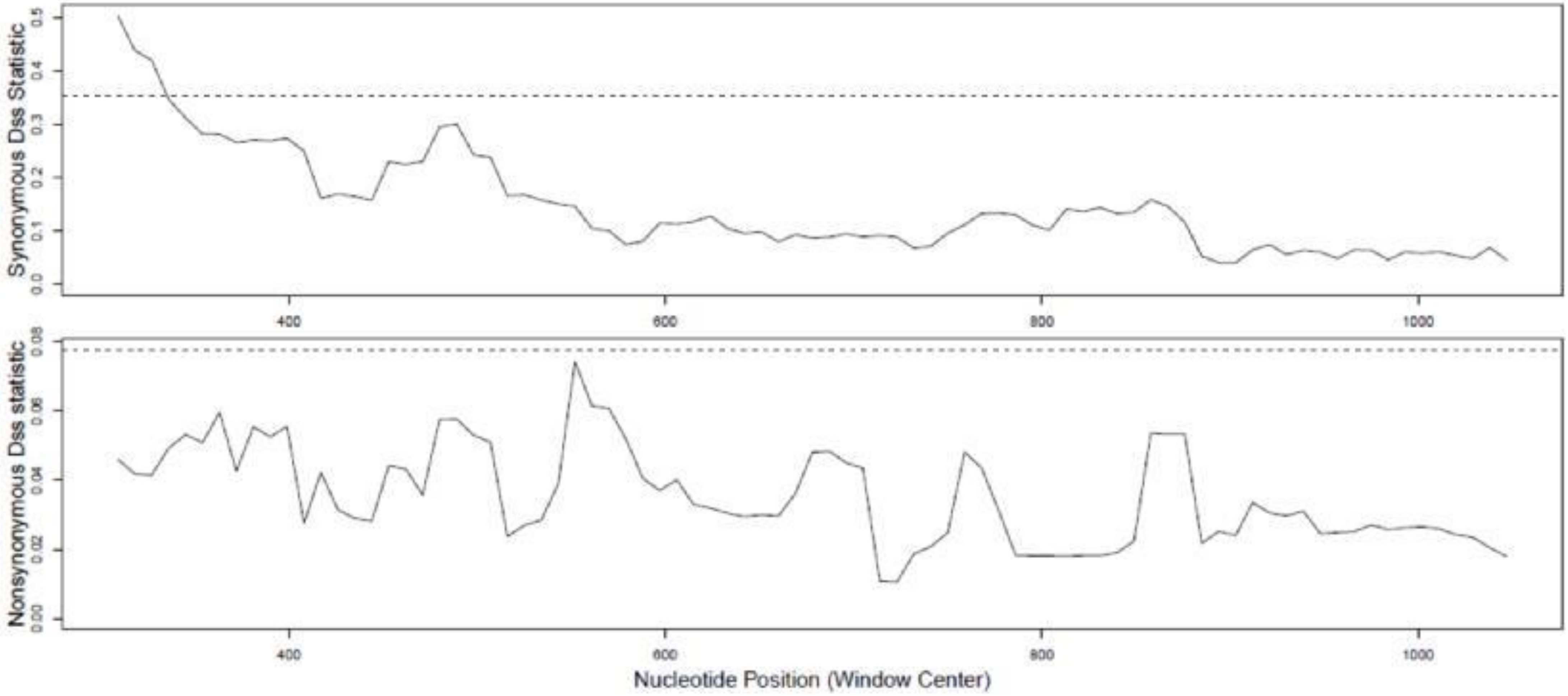

Dss statistic landscapes for synonymous and nonsynonymous changes. Dotted horizontal lines represent the 95% significance level for each test, from a parametric bootstrap with 500 replicates.

(B) 25 randomly selected core genes designated as **non-recombinants** by PhiPack (CONTD.)

HP_0420

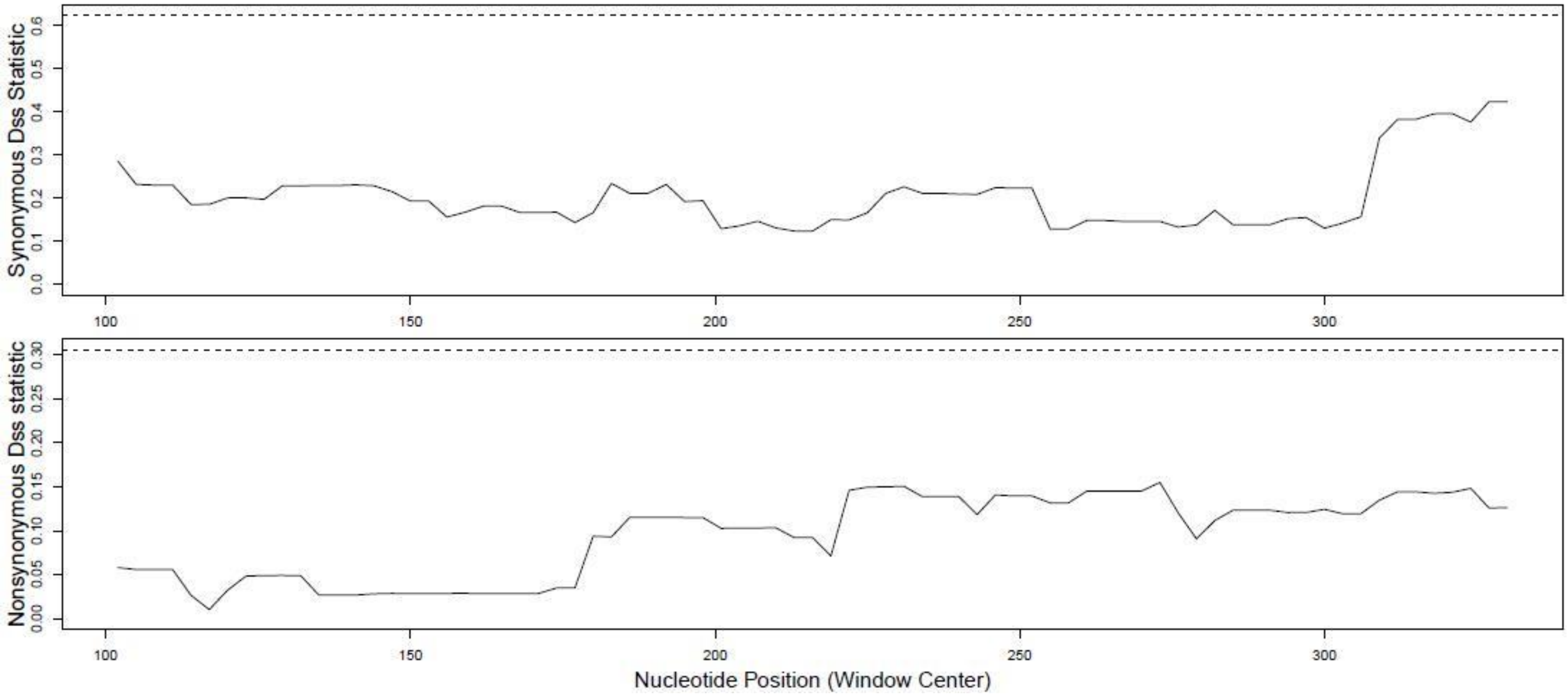

Dss statistic landscapes for synonymous and nonsynonymous changes. Dotted horizontal lines represent the 95% significance level for each test, from a parametric bootstrap with 500 replicates.

(B) 25 randomly selected core genes designated as **non-recombinants** by PhiPack (CONTD.)

HP_0736

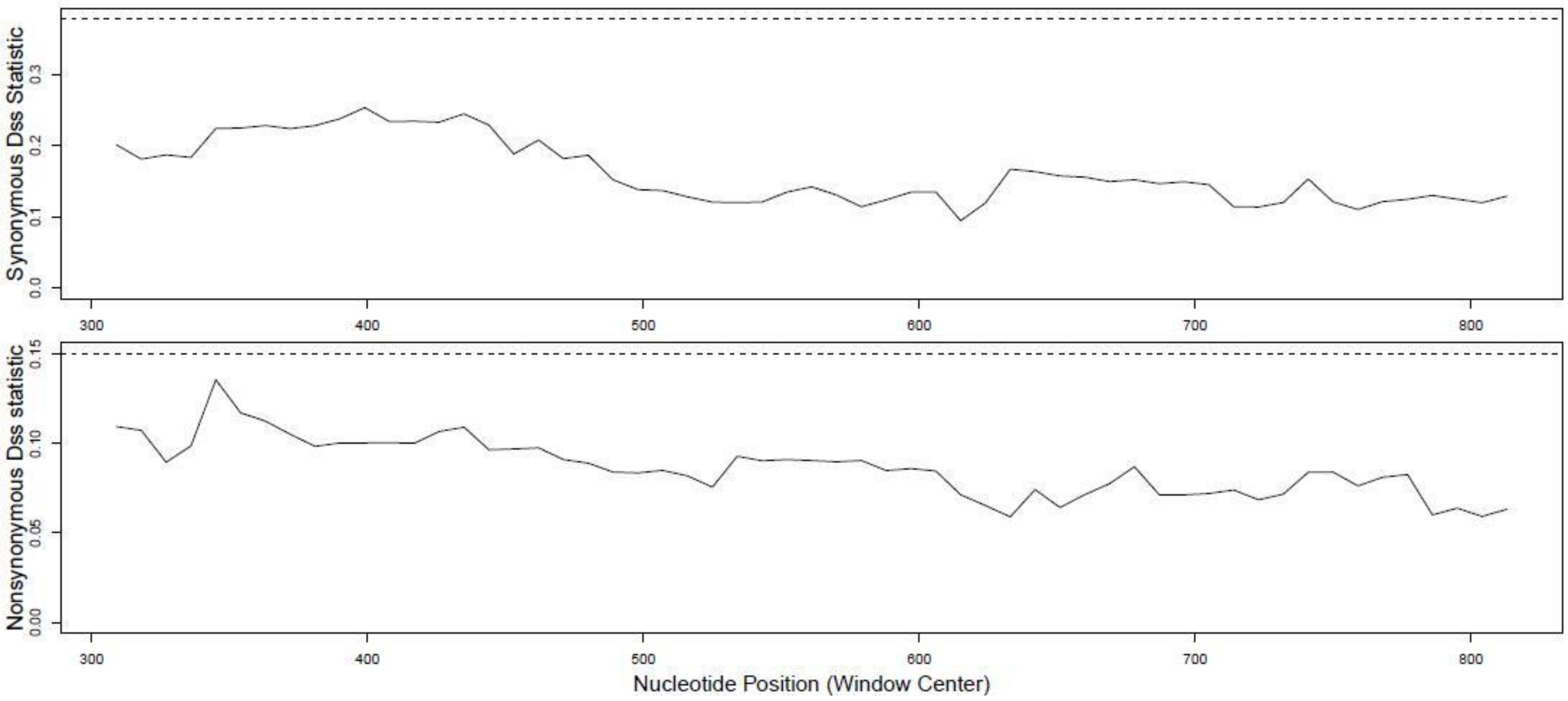

Dss statistic landscapes for synonymous and nonsynonymous changes. Dotted horizontal lines represent the 95% significance level for each test, from a parametric bootstrap with 500 replicates.

(B) 25 randomly selected core genes designated as **non-recombinants** by PhiPack (CONTD.)

HP_0742

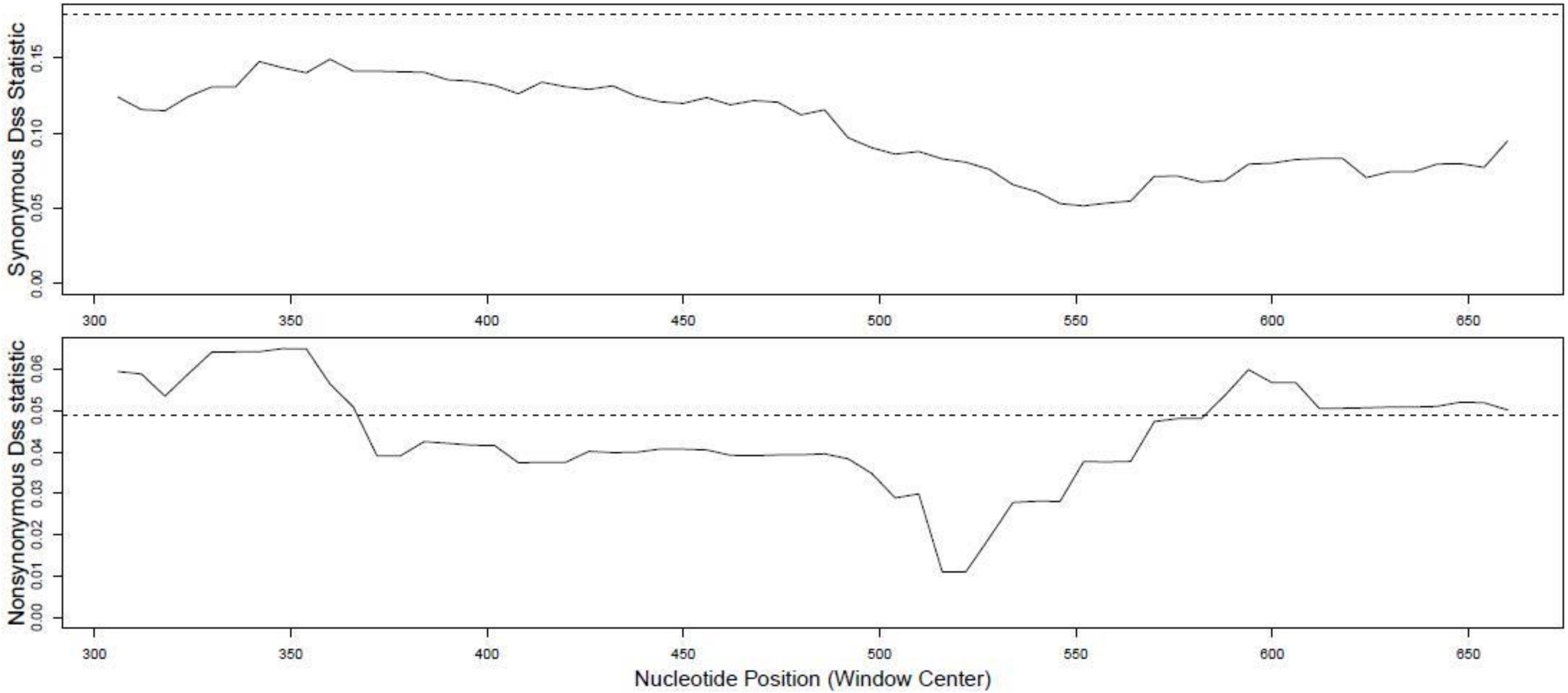

Dss statistic landscapes for synonymous and nonsynonymous changes. Dotted horizontal lines represent the 95% significance level for each test, from a parametric bootstrap with 500 replicates.

(B) 25 randomly selected core genes designated as **non-recombinants** by PhiPack (CONTD.)

HP_0771

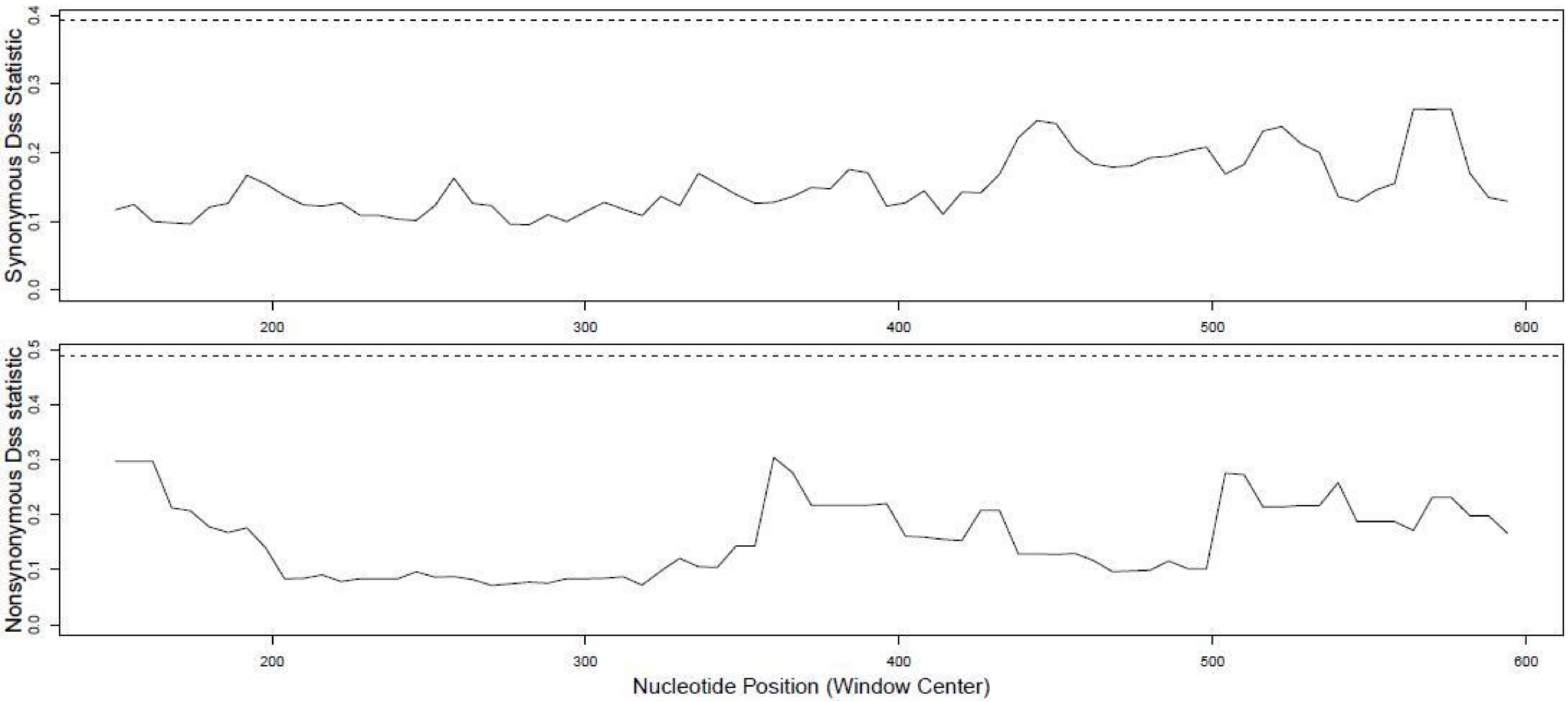

Dss statistic landscapes for synonymous and nonsynonymous changes. Dotted horizontal lines represent the 95% significance level for each test, from a parametric bootstrap with 500 replicates.

(B) 25 randomly selected core genes designated as **non-recombinants** by PhiPack (CONTD.)

HP_0884

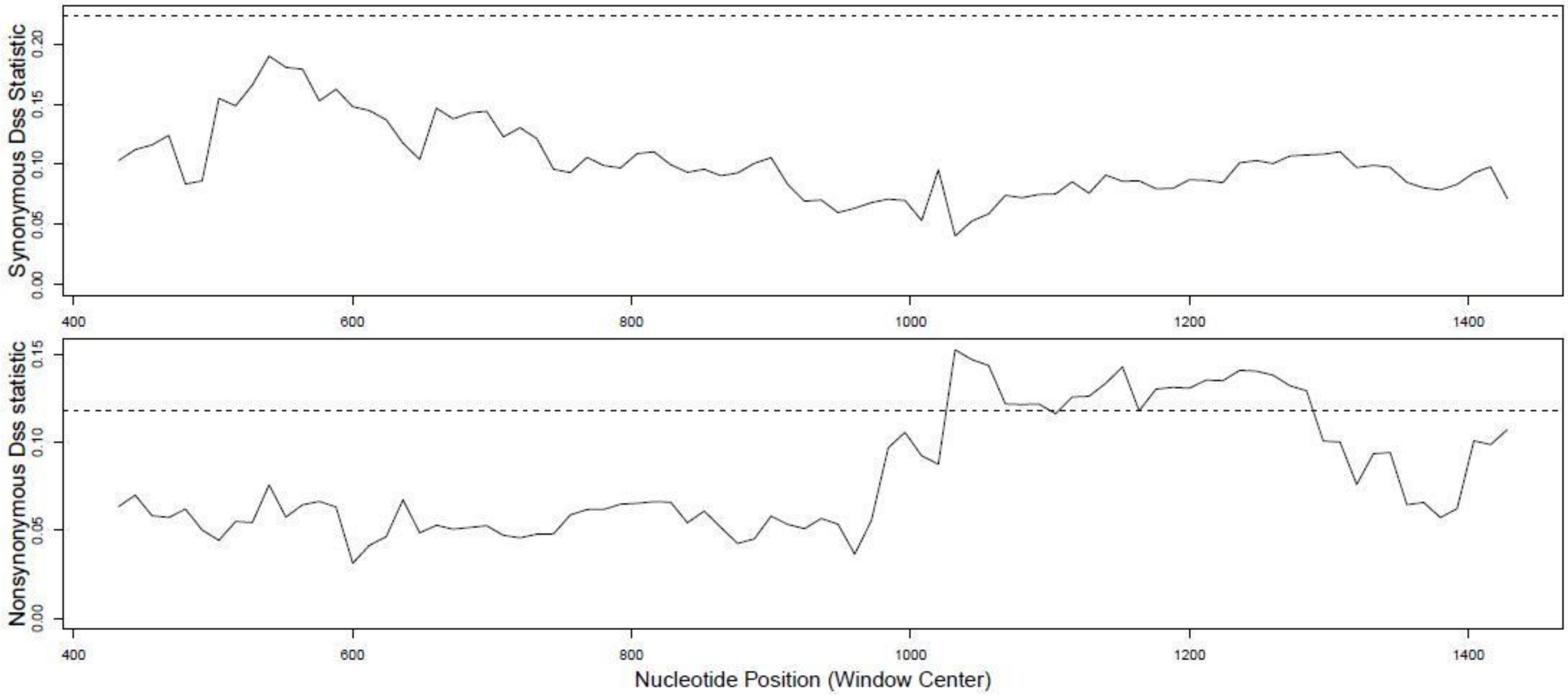

Dss statistic landscapes for synonymous and nonsynonymous changes. Dotted horizontal lines represent the 95% significance level for each test, from a parametric bootstrap with 500 replicates.

(B) 25 randomly selected core genes designated as **non-recombinants** by PhiPack (CONTD.)

HP_0908

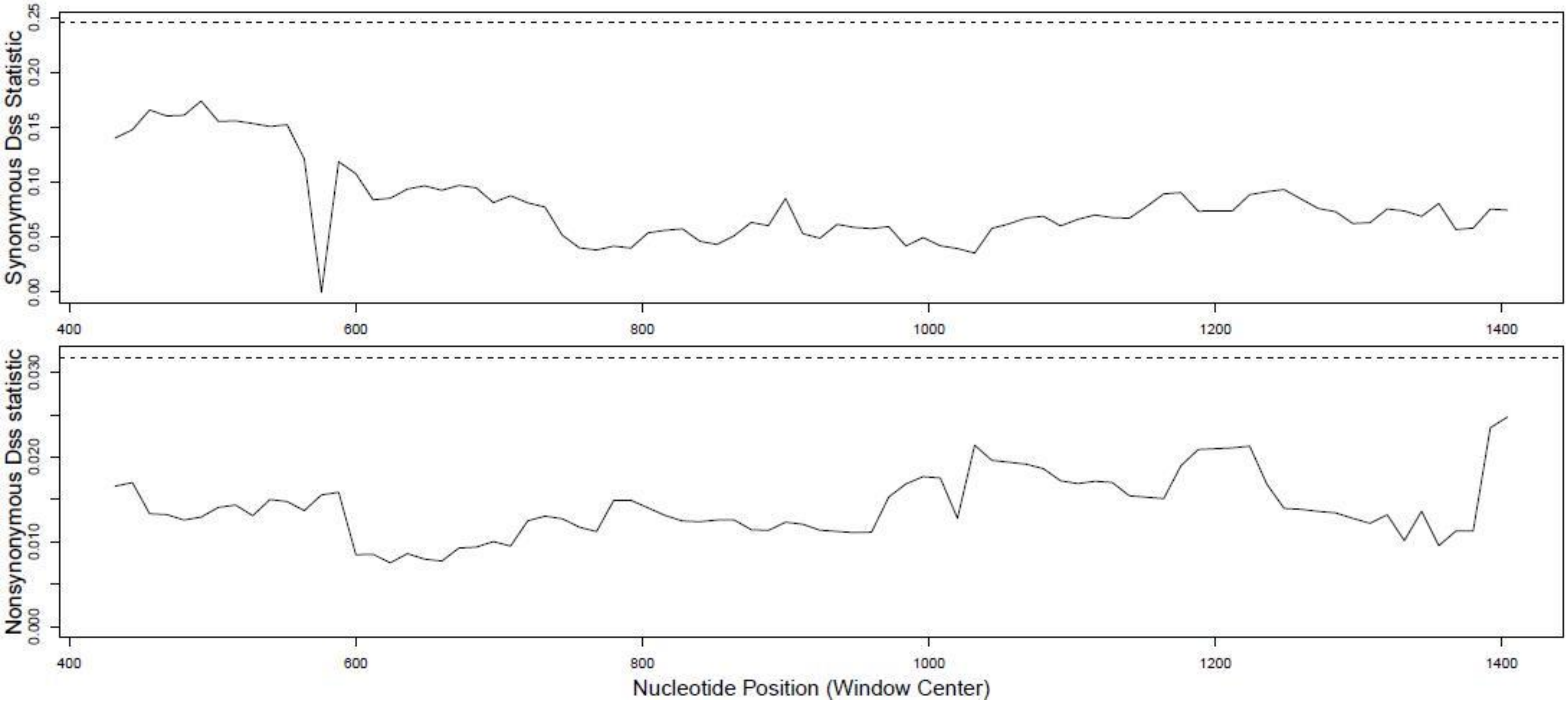

Dss statistic landscapes for synonymous and nonsynonymous changes. Dotted horizontal lines represent the 95% significance level for each test, from a parametric bootstrap with 500 replicates.

(B) 25 randomly selected core genes designated as **non-recombinants** by PhiPack (CONTD.)

HP_0933

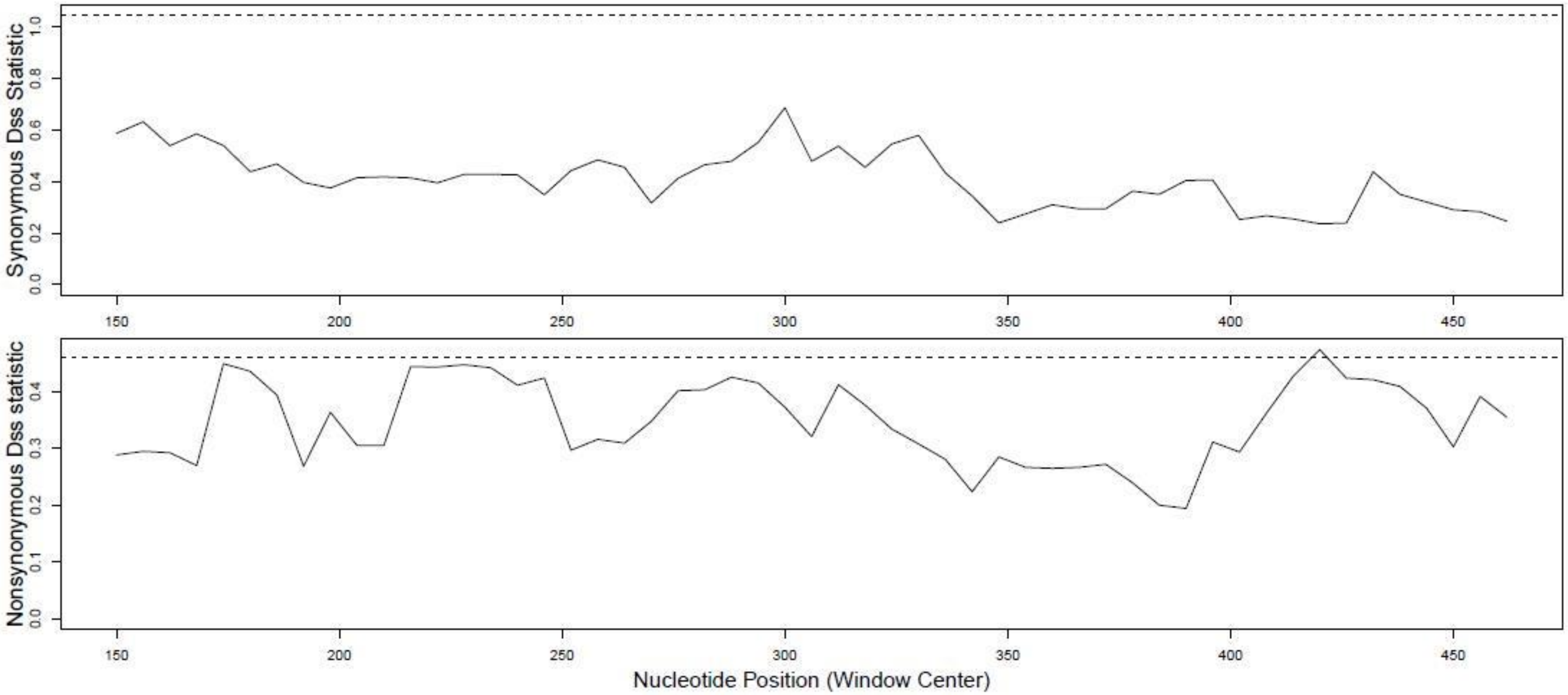

Dss statistic landscapes for synonymous and nonsynonymous changes. Dotted horizontal lines represent the 95% significance level for each test, from a parametric bootstrap with 500 replicates.

(B) 25 randomly selected core genes designated as **non-recombinants** by PhiPack (CONTD.)

HP_0950

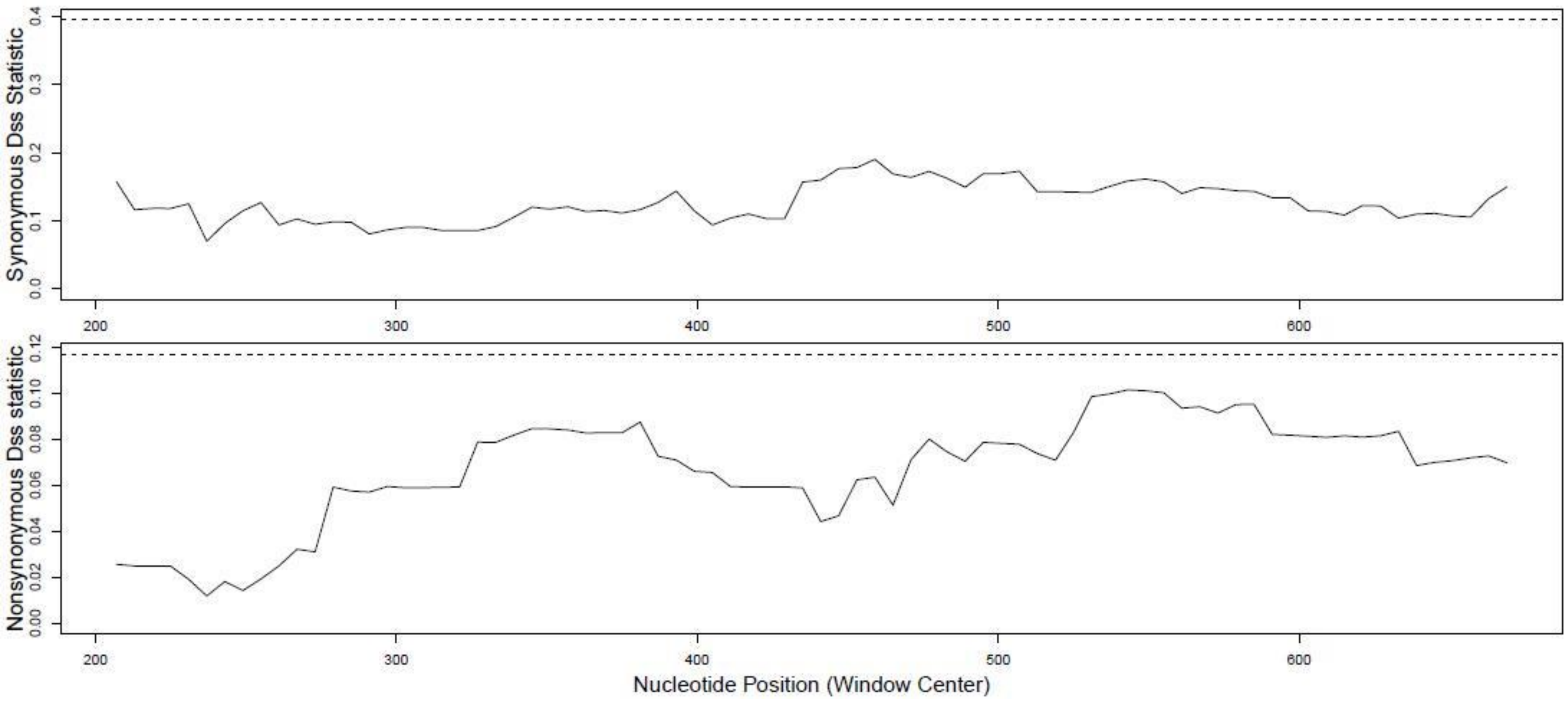

Dss statistic landscapes for synonymous and nonsynonymous changes. Dotted horizontal lines represent the 95% significance level for each test, from a parametric bootstrap with 500 replicates.

(B) 25 randomly selected core genes designated as **non-recombinants** by PhiPack (CONTD.)

HP_0958

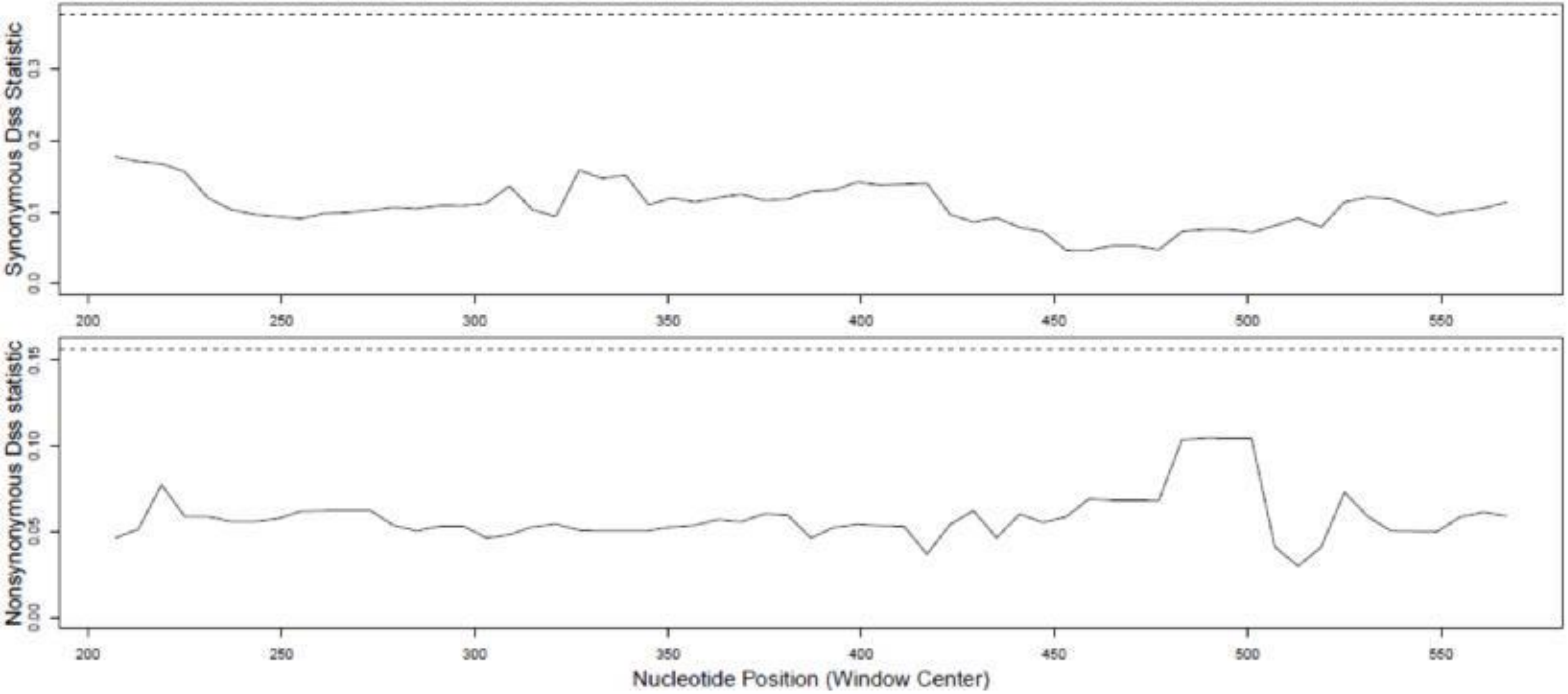

Dss statistic landscapes for synonymous and nonsynonymous changes. Dotted horizontal lines represent the 95% significance level for each test, from a parametric bootstrap with 500 replicates.

(B) 25 randomly selected core genes designated as **non-recombinants** by PhiPack (CONTD.)

HP_1016

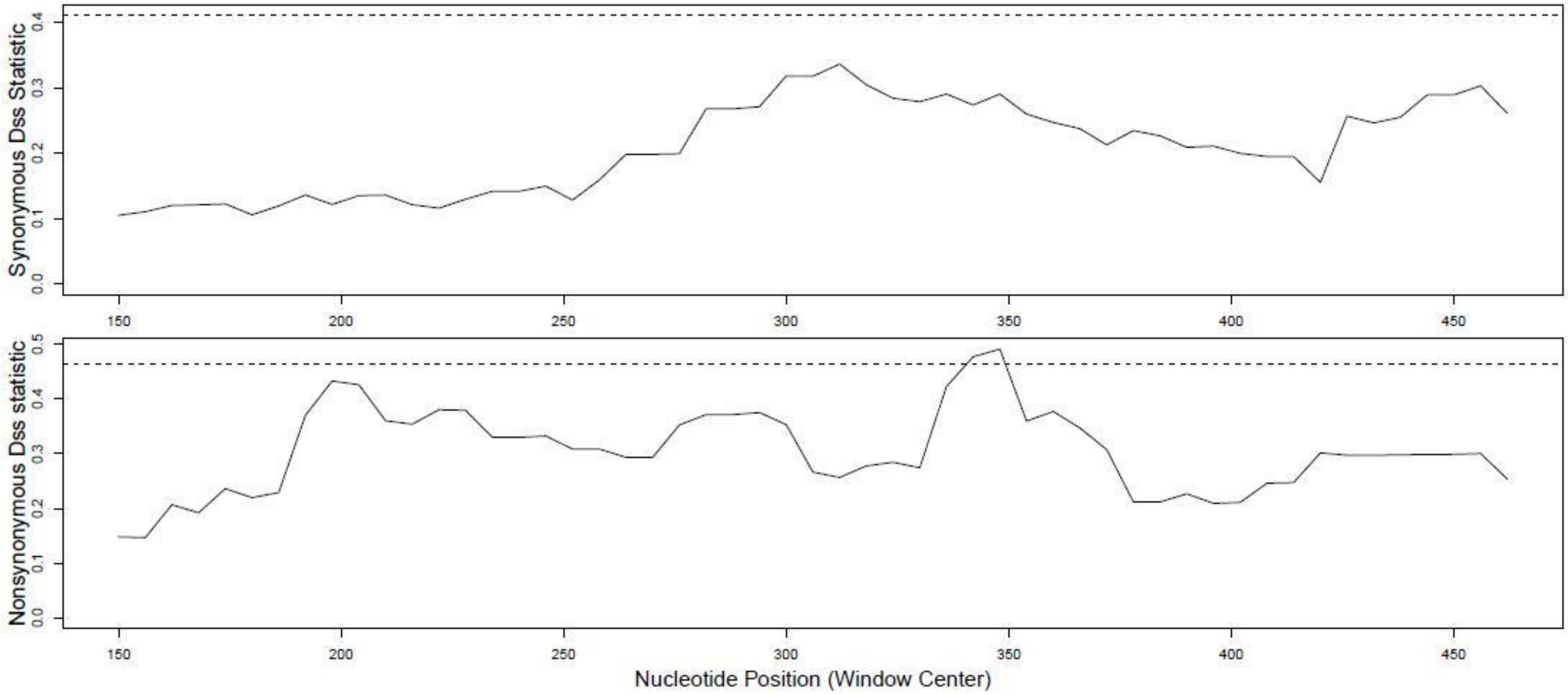

Dss statistic landscapes for synonymous and nonsynonymous changes. Dotted horizontal lines represent the 95% significance level for each test, from a parametric bootstrap with 500 replicates.

(B) 25 randomly selected core genes designated as **non-recombinants** by PhiPack (CONTD.)

HP_1050

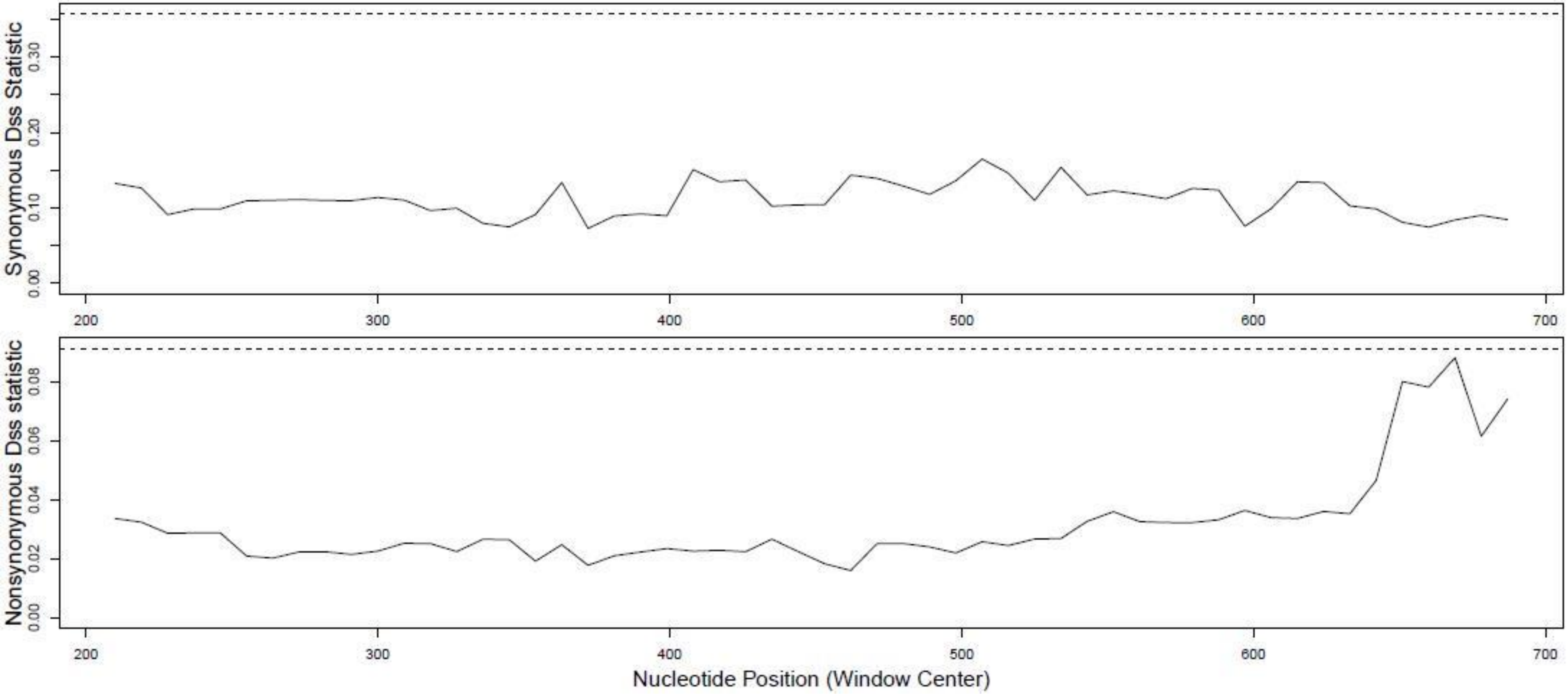

Dss statistic landscapes for synonymous and nonsynonymous changes. Dotted horizontal lines represent the 95% significance level for each test, from a parametric bootstrap with 500 replicates.

(B) 25 randomly selected core genes designated as **non-recombinants** by PhiPack (CONTD.)

HP_1190

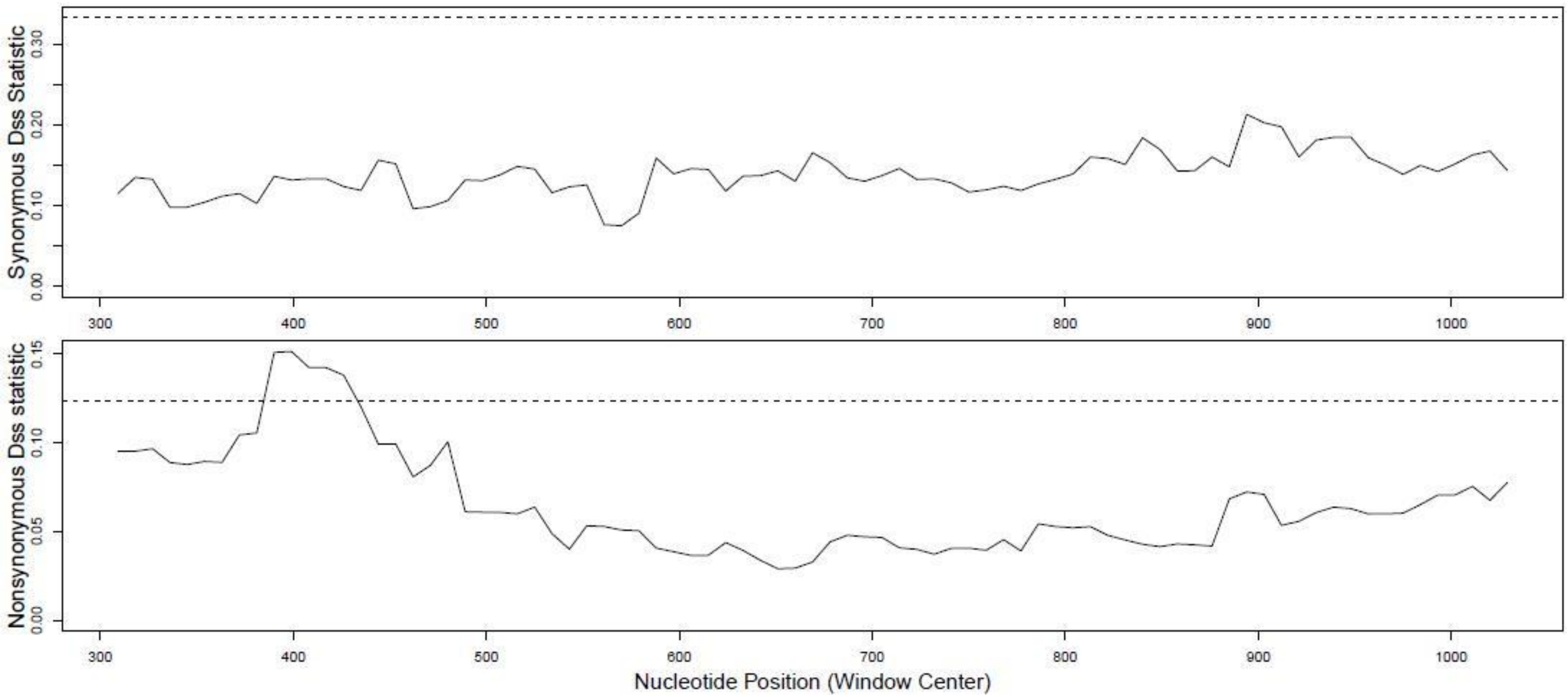

Dss statistic landscapes for synonymous and nonsynonymous changes. Dotted horizontal lines represent the 95% significance level for each test, from a parametric bootstrap with 500 replicates.

(B) 25 randomly selected core genes designated as **non-recombinants** by PhiPack (CONTD.)

HP_1203

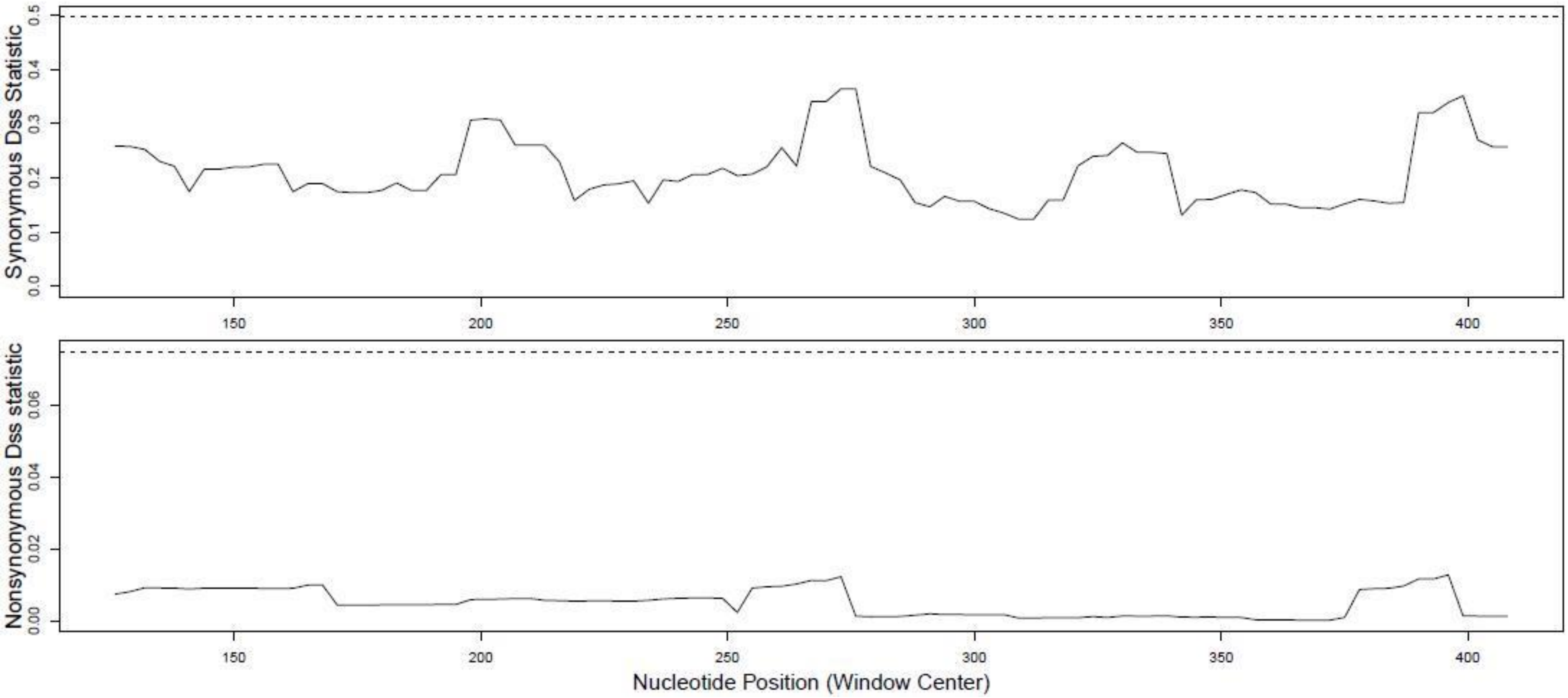

Dss statistic landscapes for synonymous and nonsynonymous changes. Dotted horizontal lines represent the 95% significance level for each test, from a parametric bootstrap with 500 replicates.

(B) 25 randomly selected core genes designated as **non-recombinants** by PhiPack (CONTD.)

HP_1269

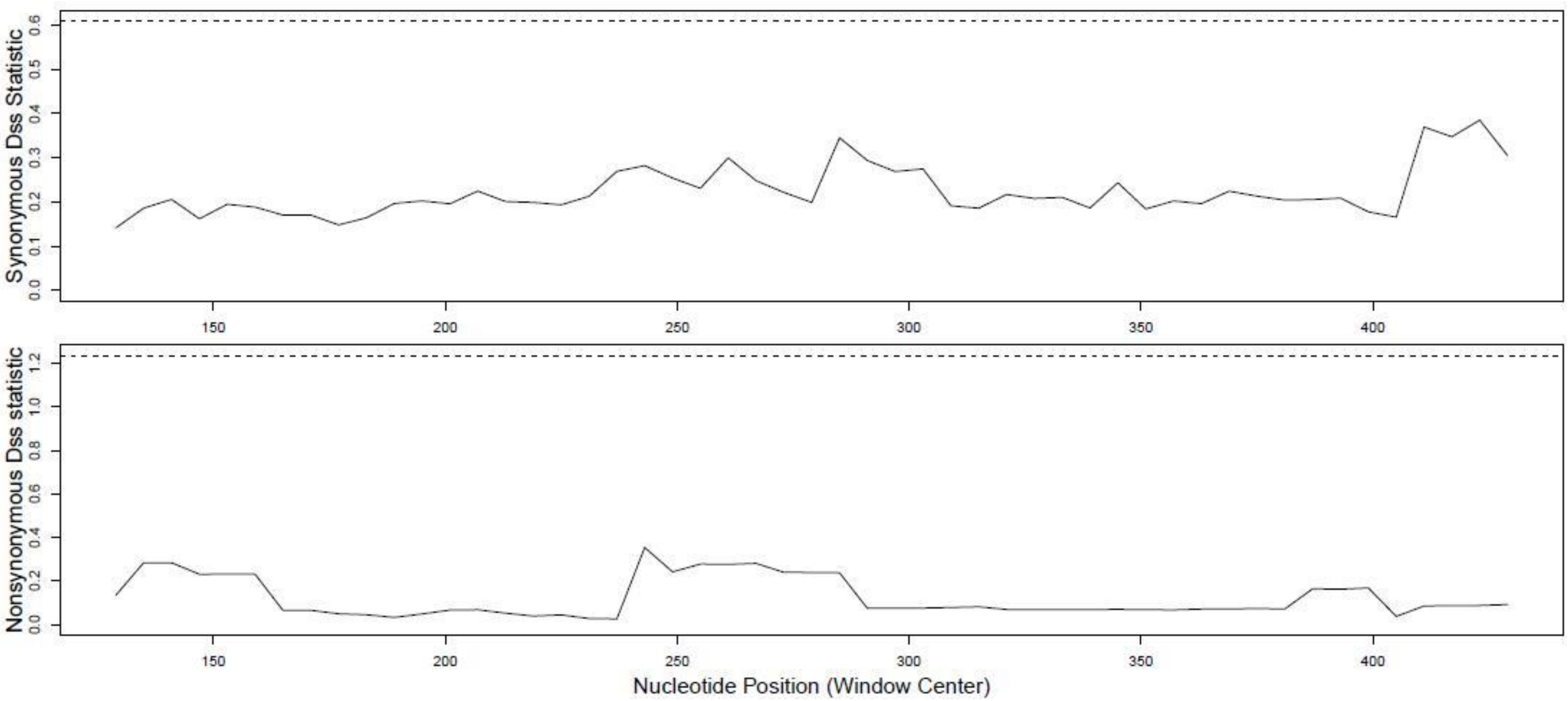

Dss statistic landscapes for synonymous and nonsynonymous changes. Dotted horizontal lines represent the 95% significance level for each test, from a parametric bootstrap with 500 replicates.

(B) 25 randomly selected core genes designated as **non-recombinants** by PhiPack (CONTD.)

HP_1299

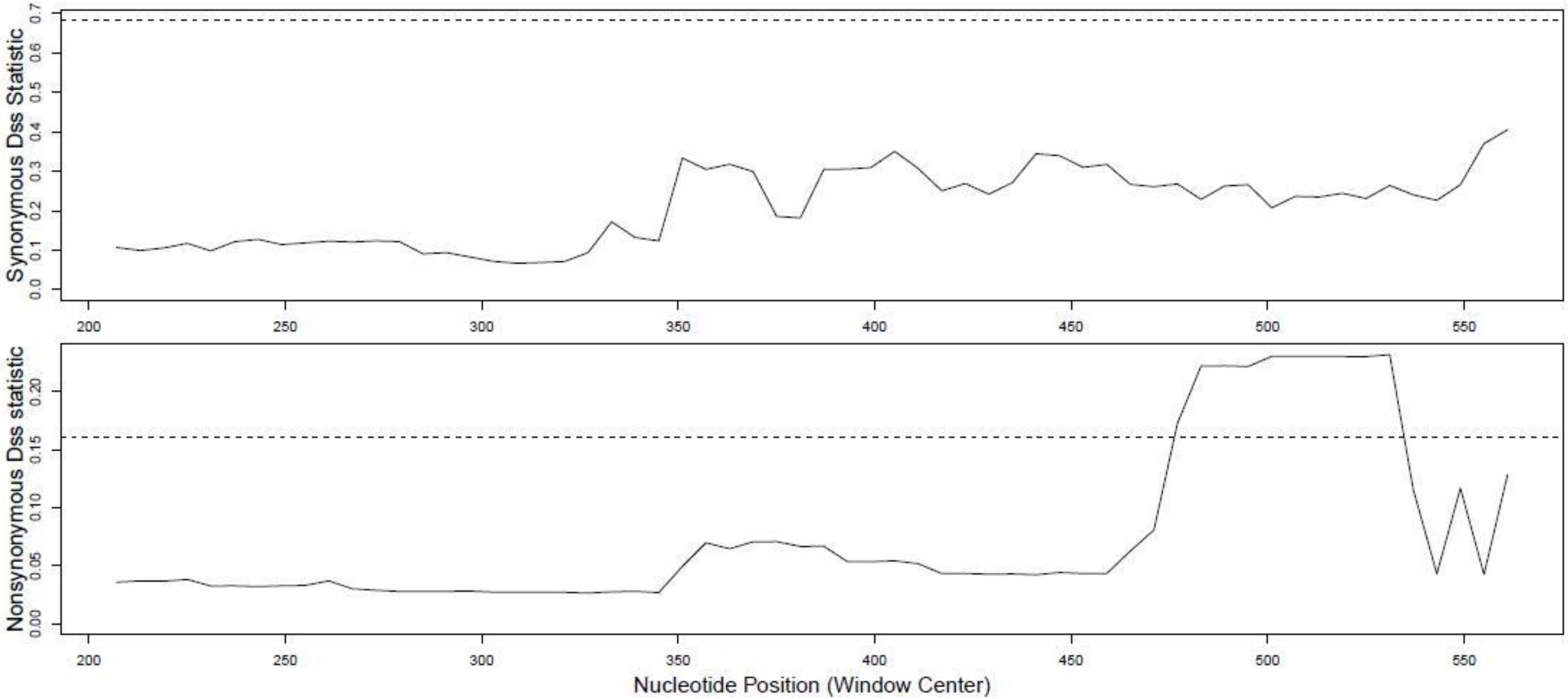

Dss statistic landscapes for synonymous and nonsynonymous changes. Dotted horizontal lines represent the 95% significance level for each test, from a parametric bootstrap with 500 replicates.

(B) 25 randomly selected core genes designated as **non-recombinants** by PhiPack (CONTD.)

HP_1305

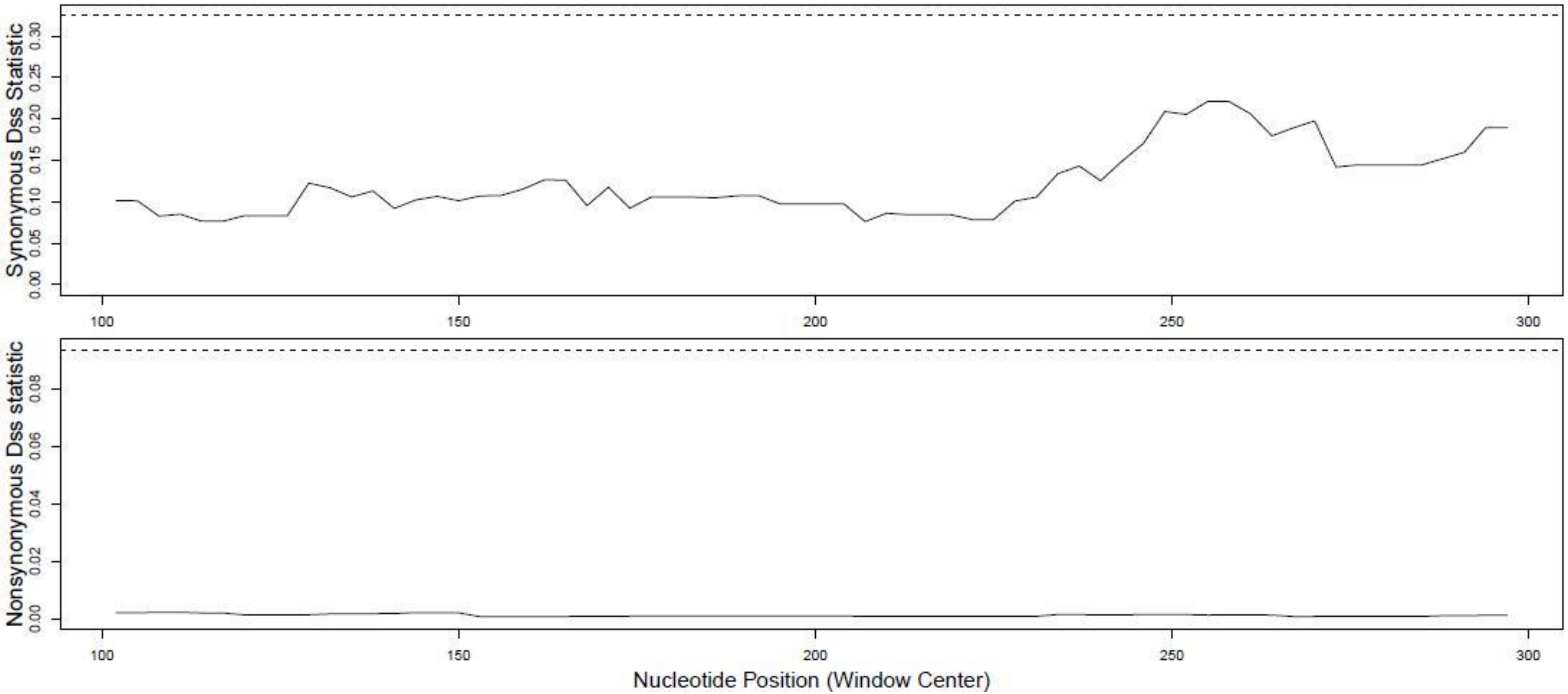

Dss statistic landscapes for synonymous and nonsynonymous changes. Dotted horizontal lines represent the 95% significance level for each test, from a parametric bootstrap with 500 replicates.

(B) 25 randomly selected core genes designated as **non-recombinants** by PhiPack (CONTD.)

HP_1559

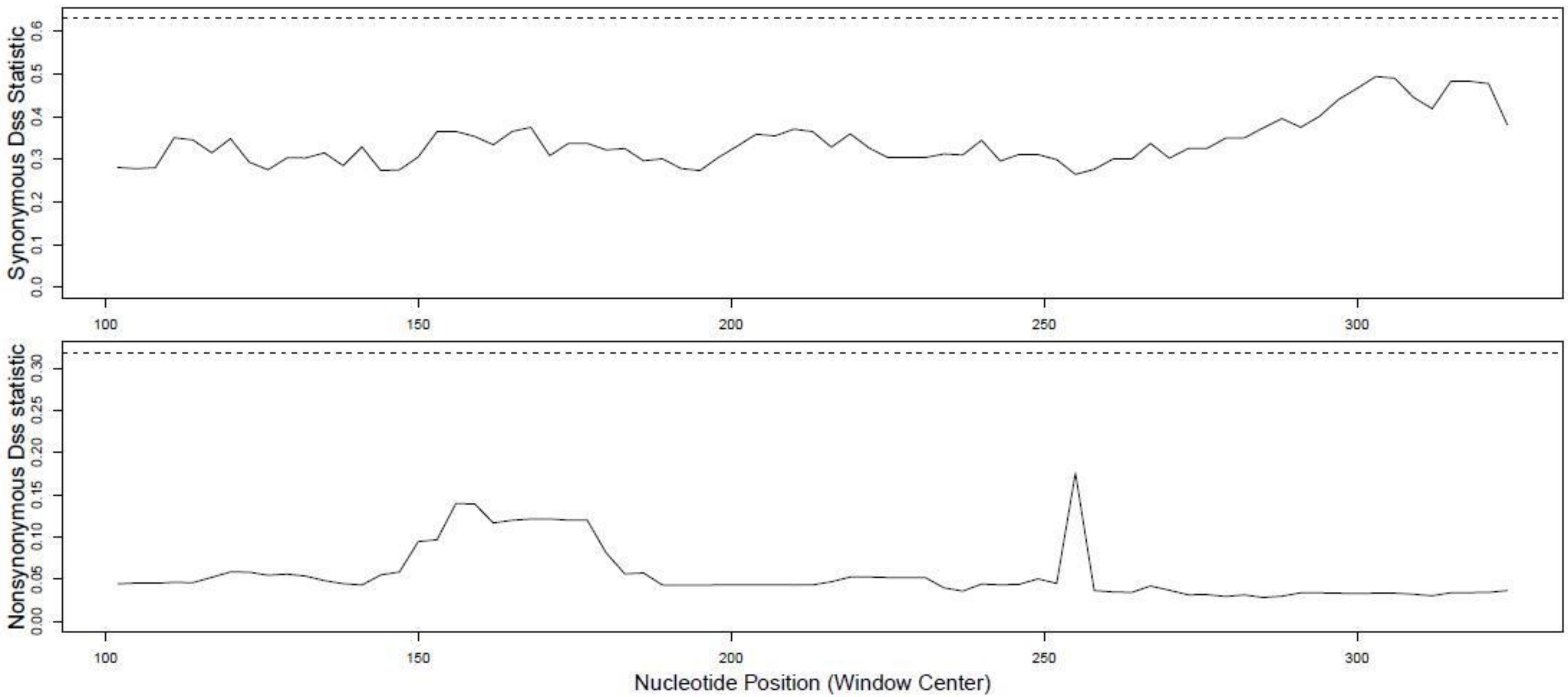

Dss statistic landscapes for synonymous and nonsynonymous changes. Dotted horizontal lines represent the 95% significance level for each test, from a parametric bootstrap with 500 replicates.

Figure S3. Average frequency distribution of mutations in encoded proteins of randomly selected subsets of *H. pylori* non-recombinant core genes with convergent mutations.

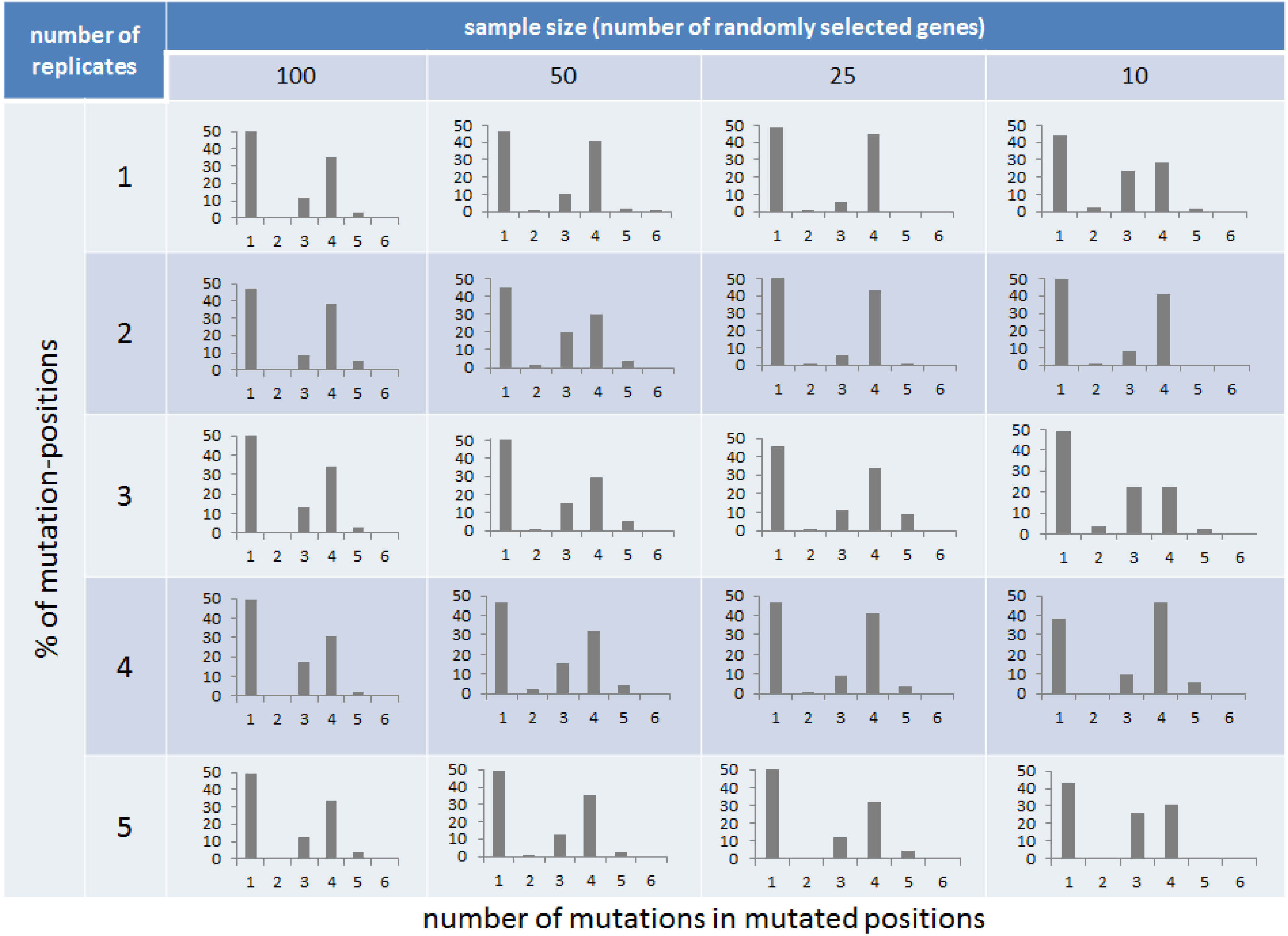

Figure S4. Average frequency distribution of mutations of encoded proteins in simulated datasets of 25 *H. pylori* genes (one of the randomly selected replicates from Figure S3) using two different mutation-rate constraints.

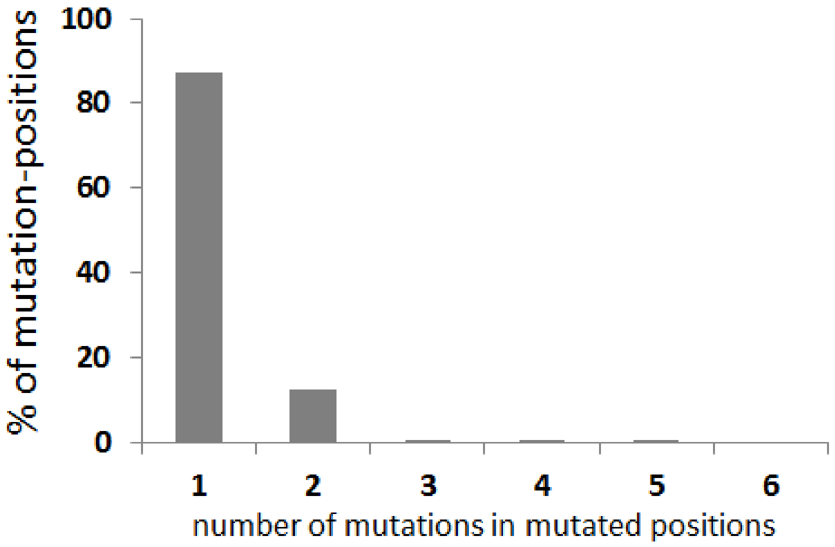

(a) Mutation rate same as of *H. pylori*

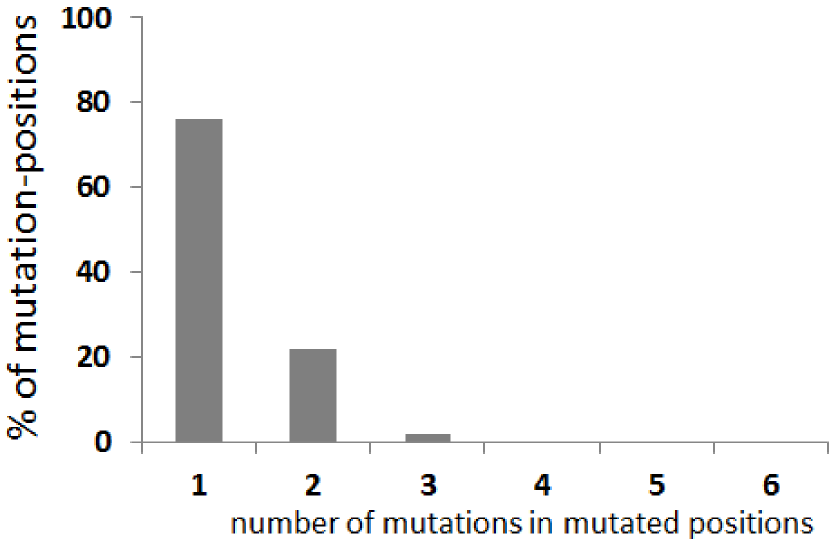

(b) Mutation rate twice of *H. pylori*
